# Ancestral State Reconstruction Reveals Rampant Homoplasy of Diagnostic Morphological Characters in Urticaceae, Conflicting with Current Classification Schemes

**DOI:** 10.1371/journal.pone.0141821

**Published:** 2015-11-03

**Authors:** Zeng-Yuan Wu, Richard I. Milne, Chia-Jui Chen, Jie Liu, Hong Wang, De-Zhu Li

**Affiliations:** 1 Key Laboratory for Plant and Biodiversity of East Asia, Kunming Institute of Botany, Chinese Academy of Sciences, Kunming, Yunnan 650201, China; 2 Germplasm Bank of Wild Species, Kunming Institute of Botany, Chinese Academy of Sciences, Kunming, Yunnan 650201, China; 3 Institute of Molecular Plant Sciences, School of Biological Sciences, University of Edinburgh, Edinburgh EH9 3JH, United Kingdom; 4 State Key Laboratory of Systematic and Evolutionary Botany, Institute of Botany, Chinese Academy of Sciences, Beijing 100093, China; 5 University of the Chinese Academy of Sciences, Beijing 100049, China; Field Museum of Natural History, UNITED STATES

## Abstract

Urticaceae is a family with more than 2000 species, which contains remarkable morphological diversity. It has undergone many taxonomic reorganizations, and is currently the subject of further systematic studies. To gain more resolution in systematic studies and to better understand the general patterns of character evolution in Urticaceae, based on our previous phylogeny including 169 accessions comprising 122 species across 47 Urticaceae genera, we examined 19 diagnostic characters, and analysed these employing both maximum-parsimony and maximum-likelihood approaches. Our results revealed that 16 characters exhibited multiple state changes within the family, with ten exhibiting >eight changes and three exhibiting between 28 and 40. Morphological synapomorphies were identified for many clades, but the diagnostic value of these was often limited due to reversals within the clade and/or homoplasies elsewhere. Recognition of the four clades comprising the family at subfamily level can be supported by a small number carefully chosen defining traits for each. Several non-monophyletic genera appear to be defined only by characters that are plesiomorphic within their clades, and more detailed work would be valuable to find defining traits for monophyletic clades within these. Some character evolution may be attributed to adaptive evolution in Urticaceae due to shifts in habitat or vegetation type. This study demonstrated the value of using phylogeny to trace character evolution, and determine the relative importance of morphological traits for classification.

## Introduction

Ancestral state reconstructions (ASR) is an increasingly popular method to map morphological or ecological traits onto a molecular phylogeny [1], which has provided plenty of novel evolutionary insights [2–6]. With it, we can examine the character evolution of traits [7–11], reveal the homology of the characters of interest, study morphological conservatism and homoplasy [12], test for variation in rates of diversification [13], detect correlated transitions between two characters in evolution [14], explore how microevolutionary processes are linked to macroevolutionary patterns in evolutionary radiations [15], and re-evaluate past classifications [6, 16].

**Fig 1:**
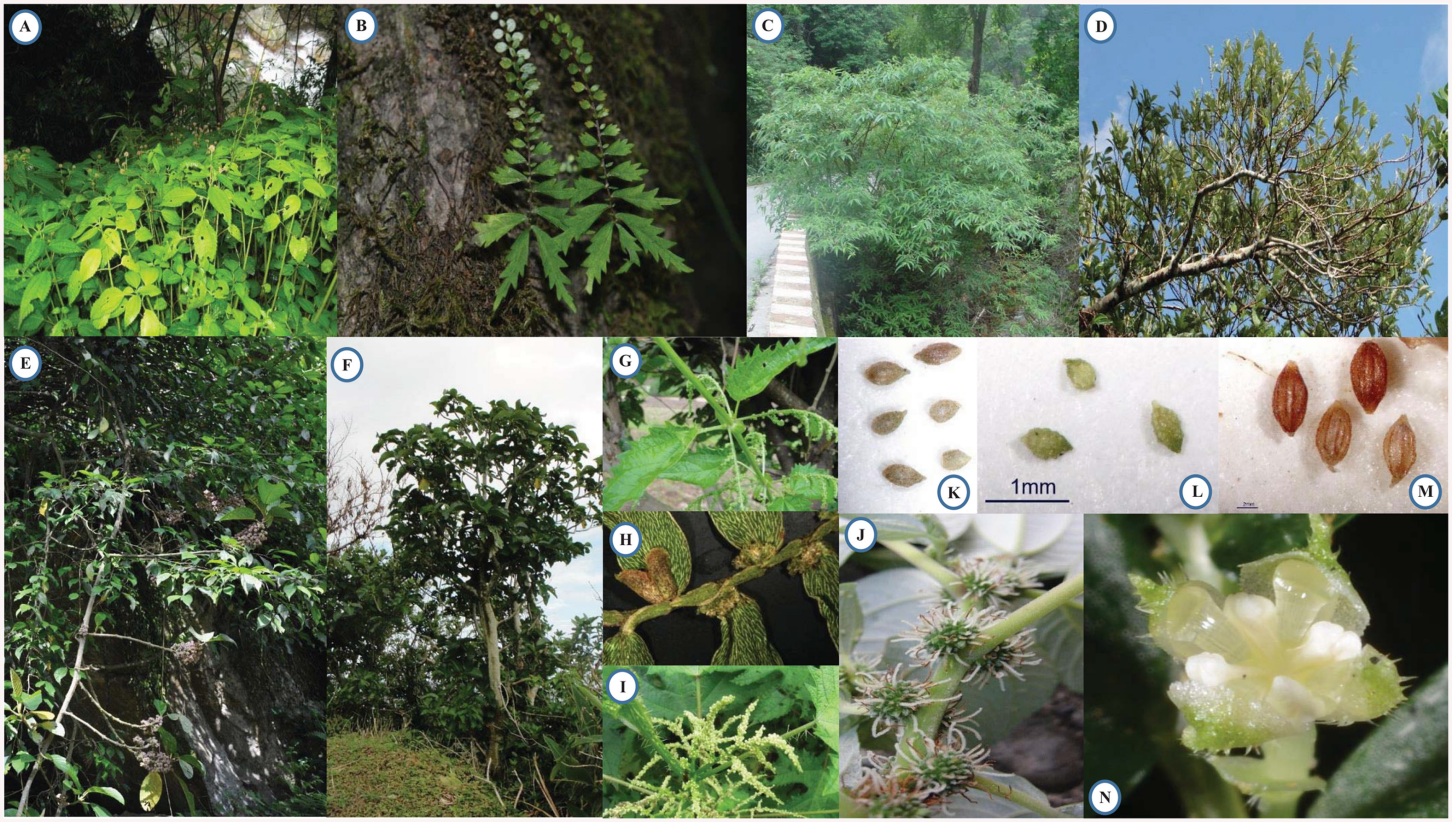
Representatives of morphological diversity in Urticaceae. A- B herbaceous habit; C- D shrubby habit; F hemi-epiphyte habit. G fused interpetiolar stipule. H linear cystolith. I stinging hair. J filiform stigma. K linolate ornamentation of achene; L tuberculate ornamentation of achene; M ribbed ornamentation of achene. N inflexed filament. All photographed by Zeng-Yuan Wu except for J by Cheng Liu. A.*Lecanthus peduncularis* (Wallich ex Royle) Weddell; B. *Elatostema monandrum* (D. Don) H. Hara; C. *Debregeasia orientalis* C. J. Chen; D. *Pipturus arborescens* (Link) C. B. Robinson; E. *Poikilospermum lanceolatum* (Trècul) Merrill; F. *Dendrocnide kotoensis* (Hayata ex Yamamoto) B. L. Shih & Yuen P. Yang; G. *Urtica thunbergiana* Siebold & Zuccarini; H. *Elatostema densistriolatum* W. T. Wang & Zeng Y. Wu; I. *Girardinia diversifolia* subsp. *Diversifolia*; J. *Pipturus arborescens* (Link) C. B. Robinson; K. *Elatostema longistipulum* Handel-Mazzetti; L. *Elatostema atroviride* W. T. Wang; M. *Elatostema imbricans* Dunn;N. *Pilea sinofasciata* C. J. Chen.

Urticaceae Juss. contains more than 2000 species encompassing a broad range of morphological diversity (Fig 1), which are distributed from tropical to temperate regions, with the largest concentration of genera and species located in tropical Asia [17–19]. It is a taxonomically difficult group, partly because many of the diagnostic characters require a microscope for accurate determination. Molecular data confirms that the family is monophyletic if Cecropiaceae is included [20, 21], but within the family many relationships that had been presumed based on morphology were contradicted, indicating homoplasy in characters previously thought to be diagnostic at genus level and above.

The last comprehensive morphological study of the family was conducted by Weddell [22], since which time knowledge of the family has increased steadily via morphological and anatomical surveys [23–30] and molecular phylogenies [20, 21]. Data from other fields such as phytochemistry, karyology, palynology, and palaeobotany are in general fragmentary [31]. There has been one morphological character study for the Urticeae, but it focused only on one tribe within the family, and the number of characters examined was very limited [32]. So there has not yet been a study that examined macromorphology of Urticaceae in the context of relationships indicated by molecular data. Characters such as habit, phyllotaxis, stipule position, stipule fusion, and pistillate perianth, have been used to divide the family into five tribes [17, 22, 33–35]. Among these characters, stigma type is highly polymorphic within most of these tribes and was usually considered useful at the between-genus level [36], whereas external morphology of achene was usually recognized as being important at the infrageneric level within Urticaceae [27].

For the same reason, scant information is available regarding major evolutionary transitions within Urticaceae. These include the origin of stinging hairs and variation among these [37]; switches in habit among herb, hemi-epiphyte, shrub and tree; inflexing of stamens until antithesis [31, 38]; and changes to stipule form, cystolith presence, and other achene characters [17, 38].

In the present study, we therefore used the existing molecular phylogeny [21] and two common analytical methods (MP and ML) to examine 19 diagnostic traits that are commonly used for taxonomic classification of Urticaceae, including all those discussed above. Our main aims were to (1) explore character evolution of these diagnostic morphological traits for Urticaceae, (2) evaluate the potential taxonomic value for these characters, and (3) identify and assess some homogeneous morphological synapomorphies, or other useful defining characters, for well-supported clades.

## Materials and Methods

### 2.1 Taxon sampling for phylogeny

We examined the same 169 accessions as for Wu *et al*. ([21]; q.v. for complete list), comprising 122 species from 47 genera of Urticaceae, representing 87% of the recognized genera [19]. The molecular data consisted of DNA sequences from two nuclear (ITS and 18S), four chloroplast *(matK*, *rbcL*, *rpll4-rps8-infA-rpl36*, *trnL-trnF*) and one mitochondrial (*matR*) loci.

### 2.2 Morphological characters and character state coding

Morphological information was mainly obtained from observations of plants in the field, and herbarium material (Studied at BM, E, K, KUN and PE); this was complemented with literature [17, 22, 27, 35, 36, 38–45]. All field work in this study was carried out on the mountains or periurban areas of China, where no specific permissions were required for these locations or activities, and no endangered or protected species were sampled. Therefore, the sampling in this study did not violate any law, rule or regulation in China and all around the world, requiring no ethical or institutional approval.

Ancestral character states were reconstructed for 19 morphological characters: habit, cystolith presence, cystolith form, stigma form, phyllotaxis, stipule presence, stipule form, stipule fusion, stipule position, pistillate perianth presence, pistillate perianth lobes fusion, achene symmetry, external morphology of achene, leaf venation apparentness, leaf venation—pinnate versus palmate, types of palmate venation, number of stamens, stinging hair presence and filament (Table 1).

**Table 1:** The coding and scoring for the characters in this study.

Characters	Character states
1 habit	0 herb 1 hemi-epiphyte 2 shrub 3 tree
2 cystolith presence	0 absent 1 present
3 cystolith form	0 punctiform 1linear 2 virgate
4 stigma form	0 penicillate 1 filiform 2 capitate 3 subulate 4 ligulate 5 peltate 6 circular 7 oblong 8 semilunar 9 spatulate
5 phyllotaxis	0 alternate 1 opposite
6 stipule presence	0 absent 1 present
7 stipule form^a^	0 lanceolate 1 broad triangle 2 narrow triangle 3 linear 4 oblong 5 ovate
8 stipule fusion	0 free 1 fused (incl partly fused) 2 amplexicaul
9 stipule position	0 intrapetiolar 1 interpetiolar
10 pistillate perianth presence	0 absent 1 present
11 pistillate perianth lobes fusion	0 free 1 connate(incl partly fused)
12 achene symmetry	0 straight 1 oblique
13 external morphology of achene	0 smooth and dull 1 ribbed 2 linolate 3 tubeculate 4 reticular 5 smooth and shiny 6 verrucose
14 leaf venation apparentness	0 inapparent 1 apparent
15 leaf venation-pinnate versus palmate	0 pinnate 1 palmate
16 types of palmate venation	0 trinerved 1 semi triplinerved^b^ 2 triplinerved 3 more than three nerves
17 number of stamens	0 more than one 1 only one
18 stinging hairspresence	0 present 1 absent
19 filament	0 inflexed 1 straight

^a)^. The five states are [46]:

**Lanceolate**: Lance-shaped; 4–5 times longer than wide; widest point between base and middle.

**Broad triangle**: Triangle-shaped; <1 times longer than wide; widest point at base.

**Narrow triangle**: Triangle-shaped; 1–2 times longer than wide; widest point at base.

**Linear**: long and narrow; >5 times longer than wide; with more or less parallel sides.

**Oblong**: oval with parallel sides for much of its length; 2–4 times longer than broad.

**Ovate**: Egg-shaped in outline, 1–2 times longer than wide, widest point between base and middle.

^b)^. **Semi triplinerved** means three-nerved, with the one lateral nerves arising from the mid-nerve above the base [47].

Regarding cystoliths, those that occur within the leaf blade or stem can be detected with a light microscope, but the character in two genera in Clade IV (*Maoutia* and *Leucosyke*) could not confidently be determined via light microscope due to their dense indumentum. Therefore we referred to spodogram-research by Bigalke [48], whose research using scanning electron microscope and stereo-light microscope determined that the cystoliths of *Leucosyke*, *Maoutia* and *Gibbsia* occur only in hairs or papillate hairs (cystolith-hairs) (Henk C. den Bakker, private communication), where they cannot be detected by light microscope. Therefore, cystoliths were coded as present only if they could be observed on the leaf blade or stem using a light microscope.

Regarding pistillate perianth lobes fusion, accessions were coded either as free (0) or connate (1), with the latter including lobes fused into a tube plus those fused only at the base. Regarding stigma, eleven types of stigma were identified by Chen [36], we detected ten types except for trifid-filiform among the taxa examined. Regarding venation, we referred to the description in the relative monographs [47, 49]. Regarding stipule form, as it is usually considered to be very important in the classification of section within Urticaceae [38], so we included this character analysis, which referred to the leaf description [46].

### 2.3 Ancestral state reconstruction

Considering that different analysis methods exhibit different advantages and limitations [50–54], we performed our analyses using both maximum parsimony (MP) and maximum likelihood (ML). Both methods were conducted in the program Mesquite v. 2.75 [55]. In the MP reconstruction, the character states were treated as unordered. We employed the Markov k-state 1 (Mk1) parameter model of evolution for the ML reconstruction, with equal probability for any particular character change. The data matrixes used for MP and ML analyses are presented in S2 and S3 Tables respectively.

## Results

**Fig 2:**
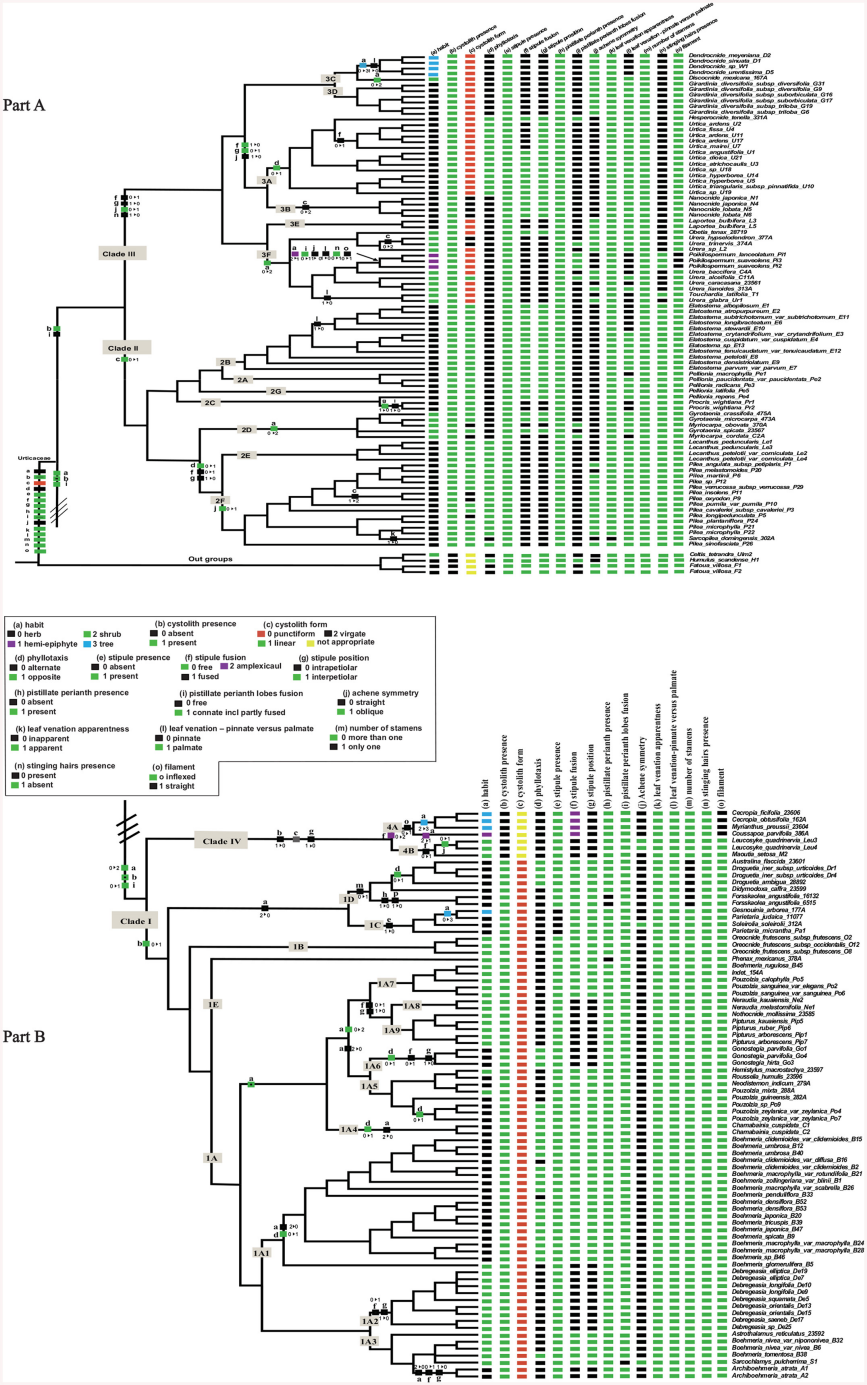
(part A+ part B). Ancestral state reconstruction of 15 selected morphological characters in Urticaceae based on the maximum parsimony analysis. The clade names and plotted numbers followed exactly the scheme of Fig 1 in [21]. The character states at the Urticaceae node indicate the ancestral states of the family. State changes are indicated as filled boxes on the branches.

Mapping the character states of 19 traits on the latest phylogenetic tree [21], overall, the reconstruction yielded no conflict between parsimony (Fig 2 and S1–S4 Figs) and likelihood analyses (S5–S23 Figs). In some cases results for ML were more equivocal for those from MP, specifically for external achene morphology. Furthermore, limited samples and sequences were available for subclade 4A (comprising four species of the genera *Cecropia*, *Myrianthus* and *Coussaspoa* from Cecropiaceae [21], hence the results of ancestral state reconstruction for this subclades are tentative. Therefore, further work include greater sampling and more DNA sequences on this subclade would be very valuable.

The following text describes the MP results; unless stated otherwise the ML results for each character were congruent from those for MP.

### 3.1 Habit

The ancestral state for Urticaceae, and also for clade II+III is herbaceous (Fig 2). There have been at least five reversions to herbaceousness in *Archiboehmeria*, *Chamabainia*, subclades 1A5+1A6, 1C+1D, and *Boehmeria* of 1A1. Hemi-epiphytes independently arose at least two times within Urticaceae, including as clear synapomorphies for *Poikilospermum* and *Coussapoa*. Shrub habit has arisen independently at least six times within Urticaceae. Tree habit independently originated at least three times, i. e. in *Dendrocnide*, *Cecropia* + *Myriocarpa* and *Gesnounia*.

### 3.2 Cystolith presence

We found that cystoliths are present in all Urticaceae except clade IV (excluding cystoliths concealed within hairs; see methods), making this a probable synapomorphy for Clade IV (Fig 2). However, the ancestral state in Urticaceae is uncertain, because cystoliths are absent in the outgroups examined, hence the ancestral state could also be absent, with presence acquired independently in Clades I and II+III. The ML analysis gave a 56% probability for absent cystoliths to be ancestral within the family (see S6 Fig).

### 3.3 Cystolith form

Three different cystolith forms occur within Urticaceae (Fig 2), and they appeared nearly stable within each of clades I, II and III. The ancestral cystolith form is punctiform, and a switch to linear is an apomorphy for Clade II that is still present in all but six of the Clade II species examined. The exceptions are four *Pilea* species, two each with punctiform and virgate cystoliths, plus both *Gyrotaenia* species also with virgate cystoliths. Outside Clade II, only *Urera alceifolia* has linear cystoliths. Clade III also has two small clades with virgate cystoliths, plus several species each in *Urtica*, *Urera* and *Poikilospermum* that can have either virgate or punctiform cystoliths. Clade I shows no change from the ancestral punctiform cystoliths except for virgate cystoliths in some specimens of *Soleirolia soleirolii*.

### 3.4 Stigma form

We determined that filiform stigmas were ancestral in Clade I (S1 Fig), whereas penicillate stigmas were ancestral in Clades II, III and IV; either of these states might be ancestral for Urticaceae, with 62% and 38% probabilities respectively, according to ML analysis (S8 Fig). Transitions to several other states occur within all four major clades, so filiform stigma is not a good diagnostic trait for Clade I.

Capitate stigmas arose at least once in each of Clades I, II and III, although shifts to other states often seem to follow. Subulate stigmas evolved independently at least 3 times within Clade III, and appeared to be a synapomorphy of two genera: *Girardinia* and *Laportea*. The ligulate stigma has occurred at least six times across clades I, III and IV, and appears to be a synapomorphy of each of *Archiboehmeria*, *Dendrocnide*, and *Poikilospermum*. The peltate stigma independently originated at least three times within Urticaceae, and it can be considered a synapomorphy of each of *Oreocnide* and *Cecropia*. Both circular and oblong stigmas arose only once, in *Sarcochlamys* and *Obetia* respectively. The semilunar stigma was unique to the two *Myriocarpa* species within subclade (2D), whereas the spatulate stigma was unique to *Touchardia*.

### 3.5 Phyllotaxis

Only two states occur in Urticaceae, of which alternate is ancestral, and opposite arose at least seven times, most commonly within Clade I (Fig 2). In addition, there have been at least three reversions to alternate phyllotaxis in *Sarcopilea*, and some species of *Boehmeria*. Opposite leaves can be considered a synapomorphy of each of 3A (*Urtica* + *Hesperocnide*), *Droguetia* +*Australina*, *Chamabainia* and *Gonostegia*. This character therefore needs to be used cautiously; however there was only one origin of opposite phyllotaxy each within Clades II (clade 2F) and III (Urtica), so it can be a useful character within those clades.

### 3.6 Stipule presence

Stipules are present in all taxa except those of subclade 1C (Fig 2). Therefore stipule presence is ancestral in the family, and their loss is a unique and diagnostic synapomorphy for subclade 1C, i.e. a reduced Parietariaeae (*Gesnouinia*, *Soleirolia*, and *Parietaria*).

### 3.7 Stipule form

All six possible states arose more than once and hence show homoplasy (S2 Fig), but a few genera do have synapomorphic stipule forms without subsequent changes, making it a useful diagnostic character for these if used with others; such genera include *Forsskaolea*, *Gonostegia*, *Dendrocnide*, *Lecanthus* and *Procris*.

Lanceolate was inferred as the ancestral state for stipules within the family (S2 Fig), but numerous transitions to other states occur in Clades I and especially II and III, including within several genera. Only a few clades lack stipule form variation, notably clades IV and 1A1+2+3, both of which retain the plesiomorphic lanceolate state throughout.

Broad triangle stipules independently evolved at least seven times, and this state can be considered a synapomorphy of each of *Nanocnide*, *Sarcopilea*, *Gonostegia*, *Forsskaolea*, and *Hemistylus*. The narrow triangle was gained at least four times throughout the family: in the common ancestors of *Pilea* and of clades 1A4+5+6+7+8+9, (both with many subsequent changes and reversions) and twice within *Elatostema*. Linear stipules arose three times, but only in Clades II and III. It appears to be a synapomorphy of *Obetia* and *Urtica*, although with two subsequent changes to oblong stipules in *Urtica*. It also arose in clade 2A+2B, but with four subsequent changes of state within this clade. Oblong stipules likewise occur only in Clades II and III, where they originated at least five times: once within *Pilea* and twice within *Urtica*, and as apparent synapomorphies for *Lecanthus* and for *Girardinia*. Ovate stipules arose at least four times, but again only in Clades II and III. This character appears synapomorphic for *Procris* and *Dendrocnide*, and otherwise arose within *Elatostema* and *Pilea*.

Overall, stipules are far more plastic within clades II+III (21 changes inferred, six possible states) that I+IV (7 changes inferred, three possible states).

### 3.8 Stipule fusion

The ancestor of Urticaceae is indicated to have had unfused (free) stipules (Fig 2) by MP analysis; this is congruent with the ML analysis (S12 Fig). We detected at least 18 switches within Urticaceae, including the common ancestor of Clades III, and those of many small clades and several genera. These included at least 12 switches to the fused stipule state, plus one clear independent origin in Clade IV, with at least five incidences of revision to free stipules. The fused state appears synapomorhic for *Procris*, Clade 2E+2F (*Lecanthus*+ *Pilea+Sarcochlarmys*), Clade IV, *Debregeasia*, and *Archiboehmeria*. Only one genus, *Urtica*, was variable for this character. Furthermore, the amplexicaul stipule was unique to subclade 4A, (Fig 2), making it a clear synapomorphy for this subclade.

### 3.9 Stipule position

Stipule position is very closely related to stipule fusion, with almost all taxa that have free stipules also having interpetiolar stipules (including the inferred common ancestor of the family), whereas fused stipules are normally intrapetiolar (Fig 2). The sole exception is *Urtica*, wherein all stipules are interpetiolar but both fused and free stipules occur, making fused interpetiolar stipules a trait unique to one clade within *Urtica*. Hence these two characters are tightly linked in their evolution, and should be treated together.

### 3.10 Pistillate perianth presence

Pistillate perianths are present almost throughout Urticaceae, and were clearly ancestral (Fig 2). Loss of pistillate perianth occurred only twice, and appears therefore to be a synapomorphy for each of *Phenax* and *Forsskaolea*.

### 3.11 Pistillate perianth lobes fusion

Free and connate tepals are ancestral to Clades II+III and I+IV respectively, and are almost diagnostic for these clades (Fig 2). The MP analysis could not determine that ancestral state for the family, but ML analysis gives a 80% likelihood for connate to be the ancestral state (S15 Fig). The lobes are free almost throughout clades II and III; although six switches to connate have occurred they affect few species: the two *Myriocarpa* species in Clade II, and in Clade III the monotypic *Hesperocnide*, two *Urera* spp, and the genus *Poikilospermum*. Only a single change has occurred in Clade I, wherein *Sarcochlamys* is unique in having free tepals.

Despite the exceptions noted, this character is the closest we have found to a diagnostic trait distinguishing the two sister clades that comprise Urticaceae.

### 3.12 Achene symmetry

Straight achenes are ancestral in Urticaceae, and in Clades I, II and IV. These clades contain respectively one (*Pilea*), two (*Sarcochlamys* and *Soleirolia*) and one (*Leucosyke*) genera in which a change to the oblique achene state appears to be a synapomorphy (Fig 2). Two reversions have also occurred within *Pilea*.

Oblique achenes are also a synapomorphy for Clade III, within which three reversals to the straight state have occurred, i.e. *Touchardia*, *Poikilospermum*, and the common ancestor of *Nanocnide* and *Urtica*.

### 3.13 External morphology of achene

The ancestral state for external achene morphology in Urticaceae was smooth-and-dull or tuberculate (S3 Fig); these two states occurred across all four major clades I-IV. ML analysis gives a higher probability for smooth-and-dull (80%) as the ancestral state (S17 Fig). Throughout the family, at least 40 changes of state (including reversals) were detected, the highest for any character examined. Three other achene states were widespread within the family: verrucose, smooth-and-shiny, and reticulate. Transititions between smooth-and-dull and verrucose were particularly common, with large clades containing a mixture of these states occuring within each of Clades I, II and III. Smooth-and-shiny achenes were gained once in Clade III (*Touchardia*) and two or three times in Clade I. Reticulate achenes arose once in each of Clades I (*Oreocnide*), II (*Gyrotaenia*) and IV (*Coussapoa*) and are hence diagnostic for those genera within those clades.

The remaining states, ribbed and linolate, occurred only in Clade II. Ribbed achenes appear synapomorphic to *Elatostema*, although switches to three different states then occurred within this genus (linolate, smooth, tuberculate). Otherwise, linolate achenes occur only in *Procris* (a likely synapomorohy) and a single species of *Pellionia*.

Nine genera exhibited within-genus variation for external achene morphology, most notably *Elatostema*, in which four of the eight different states observed by [27] were detected in our more limited sample.

### 3.14 Leaf venation apparentness

Inapparent leaf venation evolved only once within the family (Fig 2), so it can be considered as an unique synapomorphy of *Sarcopilea*.

### 3.15 Leaf venation—pinnate versus palmate

The ancestral state is palmate venation (Fig 2). Pinnate venation independently originated at least seven times, once in Clade IV and several times each in Clades II and III, often within genera. Pinnate venation is completely absent from Clade I, but can be considered as synapomorphies of *Procris*, *Dendrocnide* and *Coussapoa*. Genera that contain both states are *Urera*, *Elatostema*, *Myriocarpa* and *Pellionia*.

### 3.16 Types of palmate venation

Trinerved palmate venation is clearly ancestral within Urticaceae, and the only state present in Clade I (S4 Fig). Semi triplinerved venation arose only once, as an apomorphy for Clade 2A+2B+2C, although reversions and switches within *Elatostema* limit the diagnostic value of this character for the clade. Three *Elatostema* species were plastic for palmate venation type, with two containing both trinerved and semi triplinerved venation, whereas *Elatostema tenuicaudatum* var. *tenuicaudatum* contained both trinerved and triplinerved venation, the only instance of the latter character detected in the whole family.

Outside of clade 2A+2B+2C, all palmate-nerved species are trinerved except for a handful of transitions to >3 nerves, in a subclade of *Urtica*, *Obetia* (a possible synapomorphy), and most of Clade IV. This could be a synapomorphy for Clade IV with reversal, or it could have arisen twice within that clade. Overall, this character exhibits more changes within Clade 2A+2B+2C than among the rest of the family put together.

### 3.17 Number of stamens

The ancestral stamen number of more than one has been retained in most Urticaceae, the sole exception being subclade 1D (tribe Forsskaoleae), for which a single stamen is hence a unique synapormophy (Fig 2).

### 3.18 Stinging hairs presence

Stinging hairs were absent in the common ancestor of Urticaeae, and occur only in Clade III, for which they are clearly a synapomorphy (Fig 2). However, there were also two clear losses of this character (reversions), in *Poikilospermum* and *Touchardia*.

### 3.19 Filament

Inflexed filament was reconstructed as ancestral for Urticaceae, and occurred in all but four genera of the family (Fig 2). The exceptions were those formerly placed in Cecropiaceae, i.e. *Poikilospermum* (Clade III) plus the three genera comprising sublcade 4A of Clade IV. These had straight filaments, which hence evolved at least twice within Urticaceae.

## Discussion

The common ancestor of Urticaceae had alternate leaves with lanceolate stipules that were probably free and interpetiolar. Its cystoliths were punctiform. Its pistillate flowers had a perianth, and produced achenes that were straight and probably smooth and dull but possibly tuberculate. Its leaf venation was apparent, palmate and trinerved. Its male flowers had more than one stamen, with inflexed filaments. Hairs on its leaf blade were congruent with axis. Its habit, cystolith presence, stigma type (either filiform or penicellate) and state of pistillate perianth lobe fusion are uncertain. Notably, it had no stinging hairs.

### 4.1 Patterns of character evolution within Urticaceae

Morphological classification of Urticaceae was traditionally based on a series of key diagnostic characters including habit, cystolith form and presence, stigma, phyllotaxis, stipule presence, form and position, pistillate perianth, achene, leaf venation, stamens, stinging hairs, filament, hook hairs and so on [17, 22, 27, 33–35, 38, 56], A few other characters, like achene symmetry, have been largely overlooked. Our ancestral state reconstructions indicated that most of these traits were not good tools to classify the investigated taxa at higher levels, because most were highly homoplastic; e.g. stigma and stipule form exhibited 29 and 28 transitions respectively (S1 and S2 Figs), whereas achene surface underwent 40 transitions (S3 Fig). Hence it is unsurprising that morphology-based classifications of the genus have disagreed, both with each other and with molecular phylogenies, about the placement of certain genera.

Most characters varied independently of one another, with one exception: stipules were almost always either interpetiolar and free, or intrapetiolar and fused, despite at least nine switches for each state (Fig 2). The only exception to this pattern is a clade of four *Urtica* species (*ardens*, *fissa*, *mairei* and *zayuensis*), which uniquely have fused interpetiolar stipules. Perhaps surprisingly, these characters show no obvious correlation with phyllotaxis.

Nine of the 19 characters examined exhibited more than 10 state changes, and of these four were much more plastic within Clades II+III than in I+IV (S1 Table), whereas only habit and phyllotaxis had more transitions in Clades I and IV than II and III (Fig 2, S1 Table). Furthermore, 12 and 15 different characters were variable within Clades II and III respectively, whereas only nine were variable within Clade 1A, which though a subclade of Clade I is larger than II or III (S1 Table). This could indicate that morphological change has been slower in Clade 1A than elsewhere, that it is younger, and/or that speciation within it has been more rapid than elsewhere, in such a way that morphological change has not kept pace.

### 4.2 Taxonomic implications for tribes and subfamilies within Urticaceae

#### Clades I, II, III and IV

As previously discussed [21], Clades I, II and III broadly match existing tribes or groups thereof [35], or subfamilies of Kravtsova [23], whereas Clade IV contains one subclade of former Cecropiaceae plus two genera formerly in Boehmerieae. Here, we find that absent cystoliths (excluding those present in hairs; see methods) is a unique and consistent character for Clade IV within Urticaceae, whereas previously there were no known uniting characters for that clade. This strengthens the case for Clade IV to be taxonomically recognized at the same level as Clades I-III. Given the strong phylogenetic support for each of Clades I-IV, it seems appropriate to recognize them at subfamily level, and then apply tribal recognition to major subgroups within Clade I.

Homoplasy and reversals mean that defining characters for large clades within Urticaceae are scarce, and even the few really useful ones at higher levels have exceptions. Members of Clade II+III can normally be distinguished from those in Clade I+IV by free not connate pistillate perianth lobes (Fig 2), but there are rare exceptions.

Lecantheae (Clade II) and Urticeae (Clade III) each have one useful synapomorphy, respectively linear cystoliths and stinging hairs (Fig 2). Though both exhibit reversals in a very few species, and transitions to virgate cystoliths occur occasionally in both clades, these remain relatively useful characters given the prevalence of homoplasy and reversals in Urticaceae as a whole, and can be used with caveats as defining traits for those tribes or subfamilies. Other synapomorphies for Clades II or III, such as fused stipules and oblique achenes, exhibit too much homoplasy and reversal within these two clades to have classification value at this level.

Clade I has no unequivocal synapomorphies; filiform stigmas could be a synapomorphy for the clade but could also be ancestral to Urticaceae and lost in other clades, because they also occur in *Fatour villosa* outside of Urticaceae. This character is diagnostic for Clade I where it occurs, but due to changes within the clade not all members have it.

Therefore, with few exceptions, subfamilies based on Clades I-IV would have the following defining traits: Clade I members usually have filiform stigmas, and all except *Sarcochlamys* can be identified by a combination of connate perianth lobes with all of present cystoliths, inflexed filaments, and no stinging hairs. Most Clade II members can be identified by linear cystoliths and sometimes triplinerved/semi triplinerved leaves; however the most reliable way to identify them is free pistillate lobes but no stinging hairs; *Myriocarpa* however defies both rules. Clade III is defined by stinging hairs except for the aberrant *Touchardia* and *Poikilospermum* which must be assigned based on their particular unique trait combinations (e.g. spatulate stigma for *Touchardia*; straight anthers plus verucose achenes for *Poikilospermum*). Clade IV is defined by absent cystoliths.

#### Subgroups within Clade I

Clade I is traditionally divided into three tribes: Boehmerieae, Forsskaoleae, Parietarieae (see Table 1 in [21]). Forsskaoleae corresponds to Clade 1D and are united by a unique apomorphy of only one stamen. Parietarieae, which forms the monophyletic Clade 1C if *Rousselia* and *Pouzolzia* are moved to Boehmerieae following Kravtsova [23], are unique within the family in lacking stipules (Fig 2).

With *Leucosyke* and *Mauotia* removed, and leaving aside *Phenax* (discussed below), Boehmerieae corresponds to Clade 1A+1B+1E. However, despite of the presence of Boehmerieae in most previous classifications, we found no synapomorphies for Clade 1A+1B+1E. Instead, Boehmerieae as previously recognized appears to have contained those members of Clades I and IV that lack the distinctive traits of Forsskaoleae, Parietarieae, or Cecropiaceae. Shared characters for Boehmerieae hence seem to be those that are plesiomorphic within Clade I+IV; this explains why the phylogenetically distant *Leucosyke* and *Mauotia* were previously placed in Boehmerieae.

Therefore, recognition of Clade 1A+1B+1E as Boehmerieae fits phylogenetic data but is not supported by synapomorphies among the characters we examined. Furthermore, this clade does not have strong phylogenetic support [21], hence Boehmerieae might not be a useful subgroup to retain. Conversely clades 1B, 1E, 1A1+2+3 and 1A4+5+6+7+8+9 are all strongly supported [21], and can be consistently distinguished by achene morphology, with the sole exception of two species in clade 1A4+5+6+7+8+9 (S3 Fig). Additionally, Clade 1B (*Oreocnide*) is unique within Clade I in having peltate stigmas, whereas narrow triangle stipules are an apomorphy for 1A4+5+6+7+8+9, although reversals occur within the clade (S2 Fig). Whether such a subdivision of Boehmerieae should go ahead will depend in part on whether linking characters can be found for Clade 1A+B+1E.

The exact relationship of *Phenax* (Clade 1E) to Clades 1C+1D, 1A and 1B is unclear, with weak support [21]. Hence it could be retained within Boehmerieae or, if Boehmerieae is broken up, should become a tribe of its own. This tribe could be defined by an apomorphy of absent pistillate perianth; *Phenax* is also the only member of Clade I to retain the plesiomorphic state of tuberculate achenes, though this state has been reacquired in *Australina* of Forsskaoleae.

### 4.3 Large, paraphyletic or problematic genera within Urticaceae

Looking at the larger genera within Urticaceae, taxonomic difficulty in Clade I might reflect relatively few useful characters, whereas in Clades II and III difficulties might reflect too much homoplasy and reversals. Of those in Clade 1A, only two characters (habit and phyllotaxis) were variable within *Boehmeria*, whereas external achene morphology was the only variable character within *Debregeasia*. By comparison, *Elatostema* (Clade II) has four variable characters, whereas *Pilea* (Clade II, 2F) and *Urtica* (Clade III, 3A) have four and five respectively even if derived genera *Sarcopilea* and *Hesperocnide* are excluded. Lastly, *Urera* (Clade III) is highly paraphyletic and the clade containing it (3F) is variable for 12 different characters.

*Pilea* and *Urtica* become monophyletic if *Sarcopilea* and *Hesperocnide* are included, respectively. *Sarcopilea* has three apomorphies—alternate phyllotaxy, inapparent venation and straight achene, of which only the last has arisen independently in other *Pilea*; it also has a dramatically different distribution (Caribbean, as opposed to Old and New world tropics for *Pilea*; see Table 1 in [21]). However making all genera in Clade 2F monophyletic would divide *Pilea* into at least four genera if *Sarcopilea* isn’t included, so we suggest that *Sarcopilea domingensis* is best treated as an aberrant species of *Pilea*, whose novel traits likely result from founder effect following long dispersal. By contrast, *Hesperocnide* differs from *Urtica* only in connate perianth lobes, which given the other variation within *Urtica* noted above makes uniting these genera less problematic.

A polyphyletic *Boehmeria* is spread across subclades 1A1 and 1A3 with a single species in 1A8. Only two of the nineteen characters examined (habit and phyllotaxy) distinguish any of these species from one another, yet both of these vary within subclades and even within species. Conversely, a homoplasy for smooth achenes (S3 Fig) and a reversal to lanceolate stipules (S2 Fig) make *B*. *rugulosa* (Clade 1A8) resemble other *Boehmeria* species. Hence none of the characters we examined are at all useful in separating *Boehmeria* into segregate monophyletic genera, explaining why this has never been done before. Therefore, *Boehmeria* calls for much more detailed investigations in the future.

*Pouzolzia* Gaudich. is even more polyphyletic/paraphyletic: its members form four distinct lineages, two each within Clades 1A5 and 1A7, meaning their common ancestor is that of Clades 1A5+6+7+8+9, shared with eight other genera. Among characters examined, it appears that *Pouzolzia* is defined by two that are plesiomorphic within this clade, i.e. narrow triangle stipules (S2 Fig), and free interpetiolar stipules (Fig 2); species or clades that have altered states for one of both characters are placed in other genera. That said, the characters examined provide only limited material for a revision of this clade. Clades 1A7 and 1A8 can be separated by stipule fusion and position; stipule form would be similarly useful but for homoplasy. However clade 1A5 completely lacks detected apomorphies, and this makes a taxonomic overhaul of this group difficult. Possibly a further study similar to this one but focused only on this clade, looking at more characters, might be useful.

Relationships among *Elatostema*, *Pellionia* and *Procris* (clade 2A+B+C here) have been controversial for over a century [20, 21, 27, 47, 57–60]. *Elatostema* and *Procris* as currently subscribed are monophyletic [21], whereas *Pellionia* is paraphyletic with respect to them both, becoming monophyletic only if *P*. *repens* and *P*. *tsoongii* are moved to new, segregate genera. Among these, *Procris* can be distinguished by fused, intrapetiolar stipules. Four other characters vary within this group, i.e. stipule form, achene exterior and two venation characters (S2, S3, and S4 Figs), but all of these vary at least as much within *Elatostema* as they do outside it. Hence our results provide little data to resolve the taxonomy of this clade. A further more detailed morphological survey focused on this clade, and including additional characters such as morphology of bract and bracteoles [61], might have more success.

A third highly paraphyletic genus is *Urera*, which forms three distinct subclades of Clade 3F, each from a different biogeographic region (Africa, the Americas, Hawaii; see Fig 1 and Table 1 in [21]). The other three genera in this clade all have apomorphies unique within this clade: shiny smooth achenes and spatulate stigma for *Touchardia*, straight filament for *Poikilospermum*, and circular stigma, >3-nerved leaves and linear, fused interpetiolar stipules for *Obetia*. However, although six characters are variable within *Urera*, not one of these distinguishes the three subclades of the genus, whereas all six vary within the clade that is sister to *Poikilospermum*. Hence *Urera* appears to be a genus somewhat defined by characters that are plesiomorphic within Clade 3F, but other than biogeography we could not find defining characters that would be useful for breaking up *Urera* into monophyletic segregates.

One further pair of non-monophyletic genera are *Myriocarpa* and *Gyrotaenia*, whose species are mixed together in Clade 2D. However, these genera are separated by five of the traits examined, the two *Myriocarpa* species examined having apomorphies for virgate cystoliths, fused intrapetiolar stipules, connate female perianth lobes (unique in Clade II), and smooth-and-dull achenes. The only other character that varies within Clade 2D is leaf venation type, which distinguishes *Myriocarpa cordata* from all others. It is very unlikely that four out of five apomorphies (treating the stipule characters as one) within this clade should have occurred independently in two distinct lineages. Instead, the apparent non-monophyly of *Myriocarpa* might be a signature of some of reticulate evolution, possibly indicating a role for hybridization in this clade.

### 4.4 Adaptive evolution of characters in Urticaceae

Many of the characters examined here are likely adaptive, and hence might have responded to shifts in habitat or vegetation type [31]. Within Urticaceae, most species occur either in forests or at their margins [17], with many preferring wet places, along streams or under shade in the tropics. It is well known that convergent evolution may produce similar phenotypes in different geographically isolated regions [62], with similar conditions selecting for the same traits [63], and this could explain some of the homoplasy seen within this family. Competitive interactions might further drive switches to both homoplasious and novel character states [64]. Furthermore, some Urticaceae are pioneer species, and morphological changes could accompany switches between such habitats.

Two apparently adaptive traits that have been lost in certain lineages are stinging hairs and inflexed stamens. Stinging hairs occur in four Eudicot families (Urticaceae, Euphorbiaceae, Loasaceae, and Hydrophyllaceae [65]. Most research on stinging hairs has focused on their toxicology [37, 38, 45], ontogeny and morphology [37], whereas there has been relatively little on their evolution (but see [66]). They are affected by grazing pressure [66], and possibly also biogeographic context and competition [62]. Both instances of stinging hair loss in Urticaceae are associated with long dispersal: into SW China for *Poikilospermum* and into Hawaii for *Urera glabra* + *Touchardia*. For *Touchardia*, the loss is likely an adaptive response to the absence of native herbivorous mammals in Hawaii, but for *Poikilospermum* it could reflect a chance loss of key genes due to a founder effect, or period of low herbivore numbers at the time.

Stamens that are inflexed in the bud can explosively expel pollen by bending outwards elastically and suddenly during anthesis [31]. This is likely adaptive for wind-pollination, especially in understorey environments with limited air movement, where most Urticaceae grow. This trait is shared with the majority of Moraceae and apparently also *Celtis tetrandra* outside of Urticaceae, so the inflexed filament character may be older than the family Urticaceae. We detected independent losses in the two clades comprising former Cecropiaceae: *Poikilospermum* and *Cecropia*+*Myrianthus*+*Coussapoa*. A possible explanation for this loss is that all these three genera are tall lianas or trees, wherein elevated flowers can get more wind, which might remove the need for explosive pollen expulsion.

Overall, character evolution in Urticaceae must have been affected by a variety of ecological, genetical, and developmental factors as well as biogeography and chance events like dispersal [31, 67]. Research in all these areas will help untangle the complex causes involved, whereas a dated phylogeny might uncover instances of rapid adaptive radiation.

## Conclusion

Using ancestral state reconstructions (ASR), we found that 15 of 19 characters examined for Urticaceae exhibited at least some reversals and/or homoplasy within the family, with half exhibiting nine or more state changes. In spite of variation for some of these within some of the larger genera, such characters often had utility at distinguishing genera or small groups thereof, whereas synapomorphies for larger groupings tended to be affected by subsequent changes or reversals. Nonetheless, with the exceptions of a few atypical genera like *Myriocarpa* and *Sarcochlamys*, we determined that the four major clades that make up Urticaceae can be distinguished by carefully chosen sets of characters. This supports their recognition at subfamily level. In particular, cystolith absence from leaves and stems defines Clade IV and hence supports both its recognition as a new subfamily and the break-up of former Cecropiaceae. Within Clade I, there are clear apomorphies for Parietarieae and Forsskaoleae, but the larger Boehmerieae (Clades 1A, 1B, 1E), share only plesiomorphic characters and are also poorly supported as a monophyletic group [21]. Subdivision of Boehmerieae into four tribes would give each strong support and defining apomorphic characters.

For the polyphyletic genera *Boehmeria*, *Pouzolzia*, *Urera* and *Pellionia* we could not find useful characters to support their subdivision into monophyletic segregates. Such a task is likely to require morphological work that is focused upon each genus and its immediate relatives. Further investigation of the *Gyrotaena*/*Myriocarpa* clade is also required; we recommend the widest possible sampling of both taxa and molecular markers to investigate why *Myriocarpa* has many apomorphic traits but is not monophyletic.

Much remains to be learned about the biogeographic, ecologic and genetic factors that have affected the characters examined. *Touchardia* appears to be a classic example of dispersal to an oceanic island lacking large herbivores (Hawaii) followed by loss of defenses (stinging hairs), yet even this example is complicated by a sister species on the same archipelago whose stinging hairs remain.

Therefore, higher level investigations and especially detailed surveys of problem genera are necessary to further improve our understanding of this family, and give further support to a revision of generic and tribal classifications within the family.

## Supporting Information

S1 Fig Ancestral state reconstruction for Urticaceae based on parsimony of stigma form. (PDF)

S2 Fig Ancestral state reconstruction for Urticaceae based on parsimony of stipule form. (PDF)

S3 Fig Ancestral state reconstruction for Urticaceae based on parsimony of external morphology of achene. (PDF)

S4 Fig Ancestral state reconstruction for Urticaceae based on parsimony of types of palmate vernation. (PDF)

S5 Fig Ancestral state reconstruction for Urticaceae based on Maximum likelihood optimisation of habit. (PDF)

S6 Fig Ancestral state reconstruction for Urticaceae based on Maximum likelihood optimisation of cystolith presence. (PDF)

S7 Fig Ancestral state reconstruction for Urticaceae based on Maximum likelihood optimisation of cystolith form. (PDF)

S8 Fig Ancestral state reconstruction for Urticaceae based on Maximum likelihood optimisation of stigma form. (PDF)

S9 Fig Ancestral state reconstruction for Urticaceae based on Maximum likelihood optimisation of phyllotaxis. (PDF)

S10 Fig Ancestral state reconstruction for Urticaceae based on Maximum likelihood optimisation of stipule presence. (PDF)

S11 Fig Ancestral state reconstruction for Urticaceae based on Maximum likelihood optimisation of stipule form. (PDF)

S12 Fig Ancestral state reconstruction for Urticaceae based on Maximum likelihood optimisation of stipule fusion. (PDF)

S13 Fig Ancestral state reconstruction for Urticaceae based on Maximum likelihood optimisation of stipule position. (PDF)

S14 Fig Ancestral state reconstruction for Urticaceae based on Maximum likelihood optimisation of pistillate perianth presence. (PDF)

S15 Fig Ancestral state reconstruction for Urticaceae based on Maximum likelihood optimisation of pistillate perianth lobes fusion. (PDF)

S16 Fig Ancestral state reconstruction for Urticaceae based on Maximum likelihood optimisation of achene symmetry. (PDF)

S17 Fig Ancestral state reconstruction for Urticaceae based on Maximum likelihood optimisation of external morphology of achene. (PDF)

S18 Fig Ancestral state reconstruction for Urticaceae based on Maximum likelihood optimisation of leaf venation apparentness. (PDF)

S19 Fig Ancestral state reconstruction for Urticaceae based on Maximum likelihood optimisation of leaf venation-pinnate versus palmate. (PDF)

S20 Fig Ancestral state reconstruction for Urticaceae based on Maximum likelihood optimisation of types of palmate vernation. (PDF)

S21 Fig Ancestral state reconstruction for Urticaceae based on Maximum likelihood optimisation of number of stamens. (PDF)

S22 Fig Ancestral state reconstruction for Urticaceae based on Maximum likelihood optimisation of stinging hairs presence. (PDF)

S23 Fig Ancestral state reconstruction for Urticaceae based on Maximum likelihood optimisation of filament. (PDF)

S1 Table Minimum number of origins for characters states examined, within Urticaceae. (DOCX)

S2 Table Showing for each taxon examined where the information for each character came from in this study (literature/field work/herbarium). (DOCX)

S3 Table The data matrixes of MP. (DOCX)

S4 Table The data matrixes of ML. (DOCX)
