# Supplementary figures and images for: Ancestral State Reconstruction Reveals Rampant Homoplasy of Diagnostic Morphological Characters in Urticaceae, Conflicting with Current Classification Schemes
Source: PLoS One. 2015 Nov 3;10(11):e0141821. doi: 10.1371/journal.pone.0141821 (PMC4631448; doi:10.1371/journal.pone.0141821)

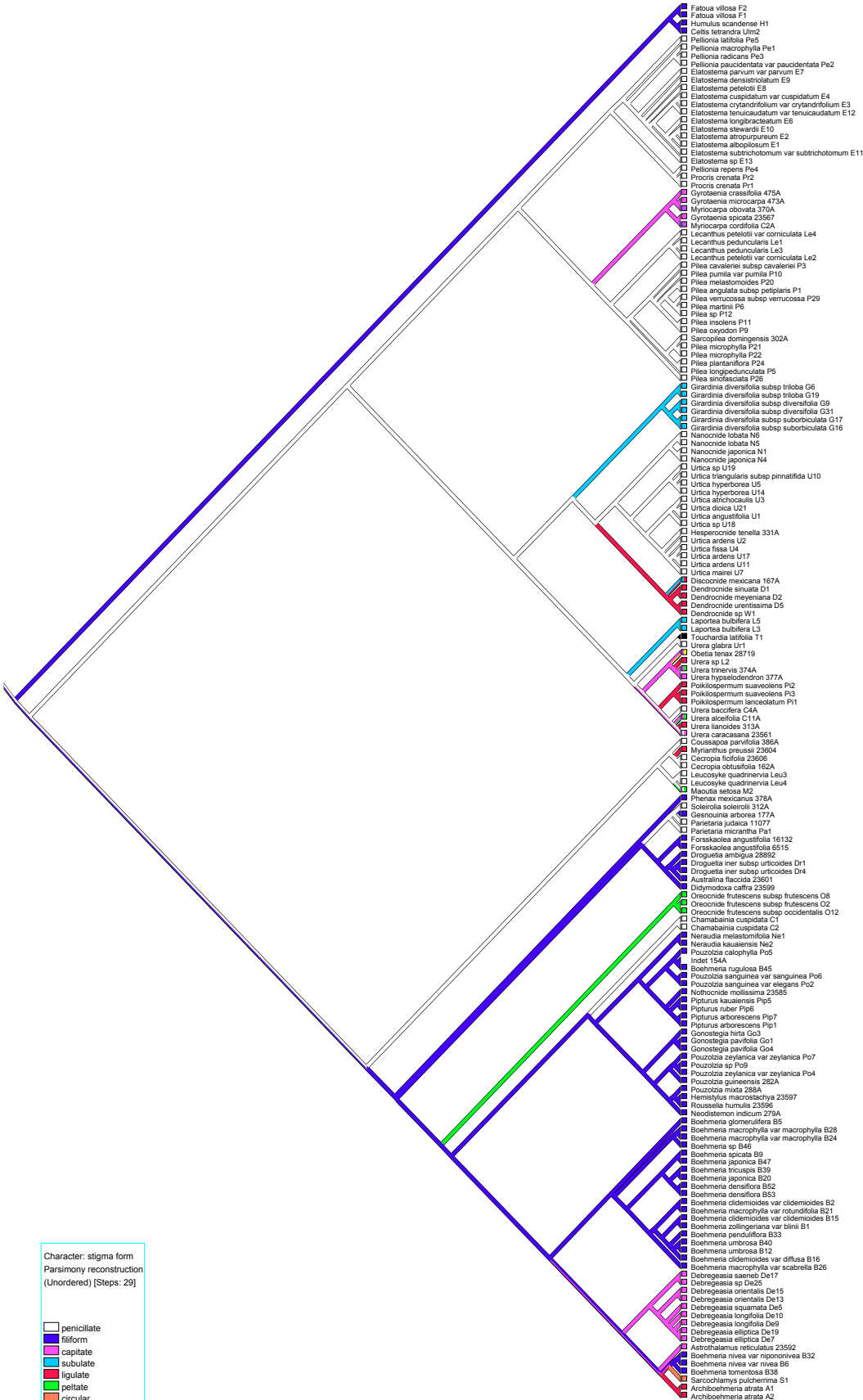

Supplement: S1 Fig — (PDF) [file pone.0141821.s001.pdf]

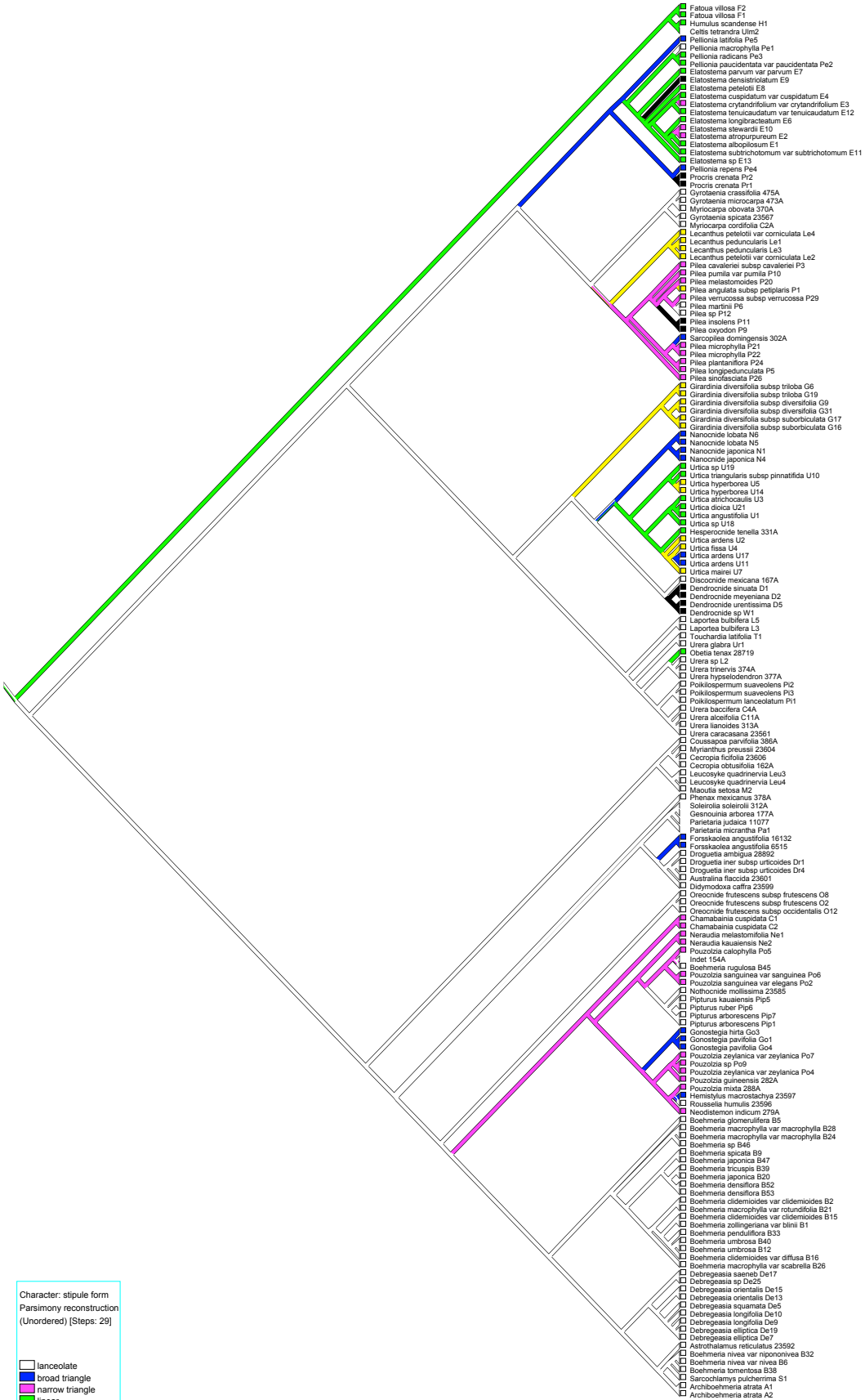

Supplement: S2 Fig — (PDF) [file pone.0141821.s002.pdf]

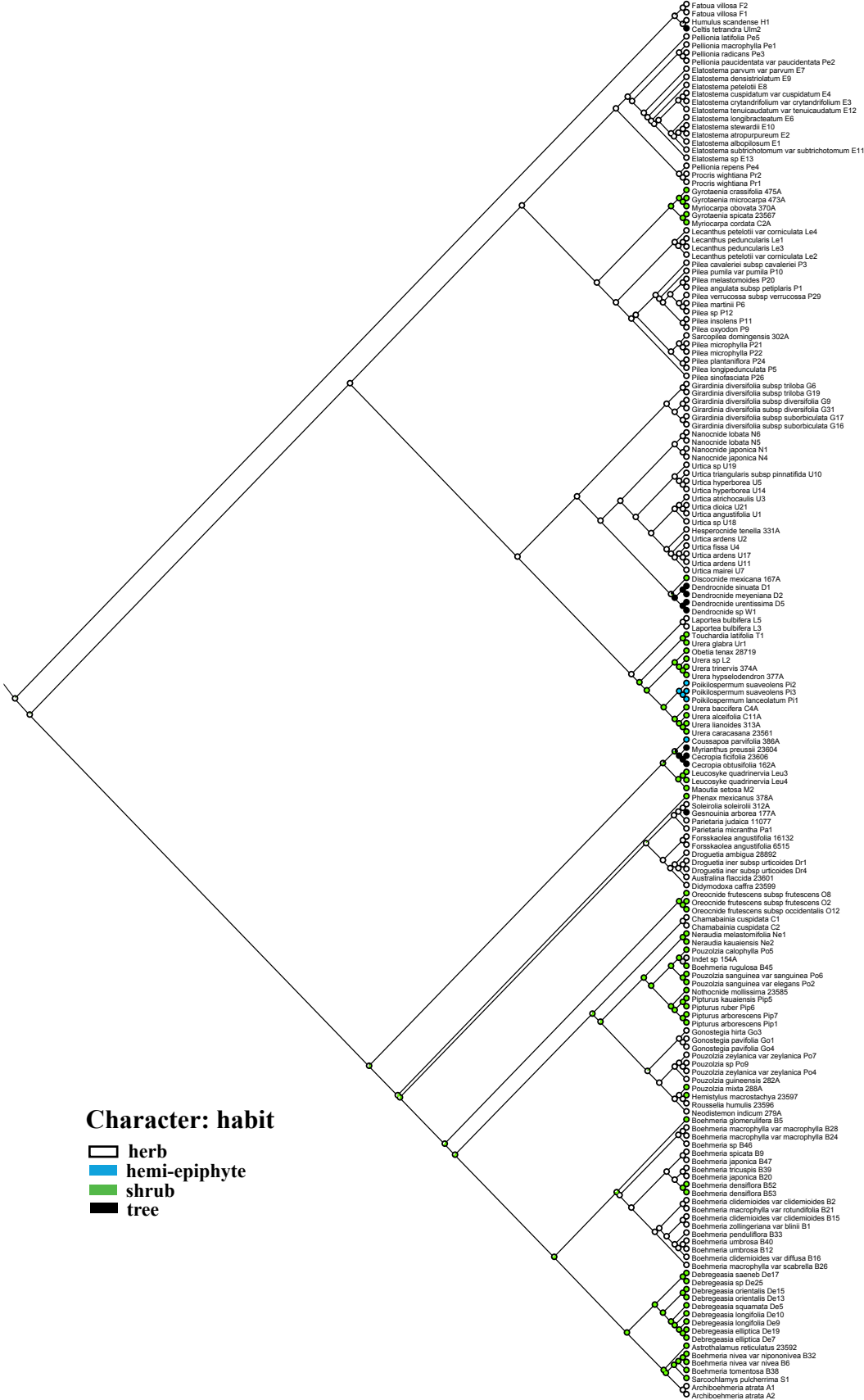

Supplement: S5 Fig — (PDF) [file pone.0141821.s005.pdf]

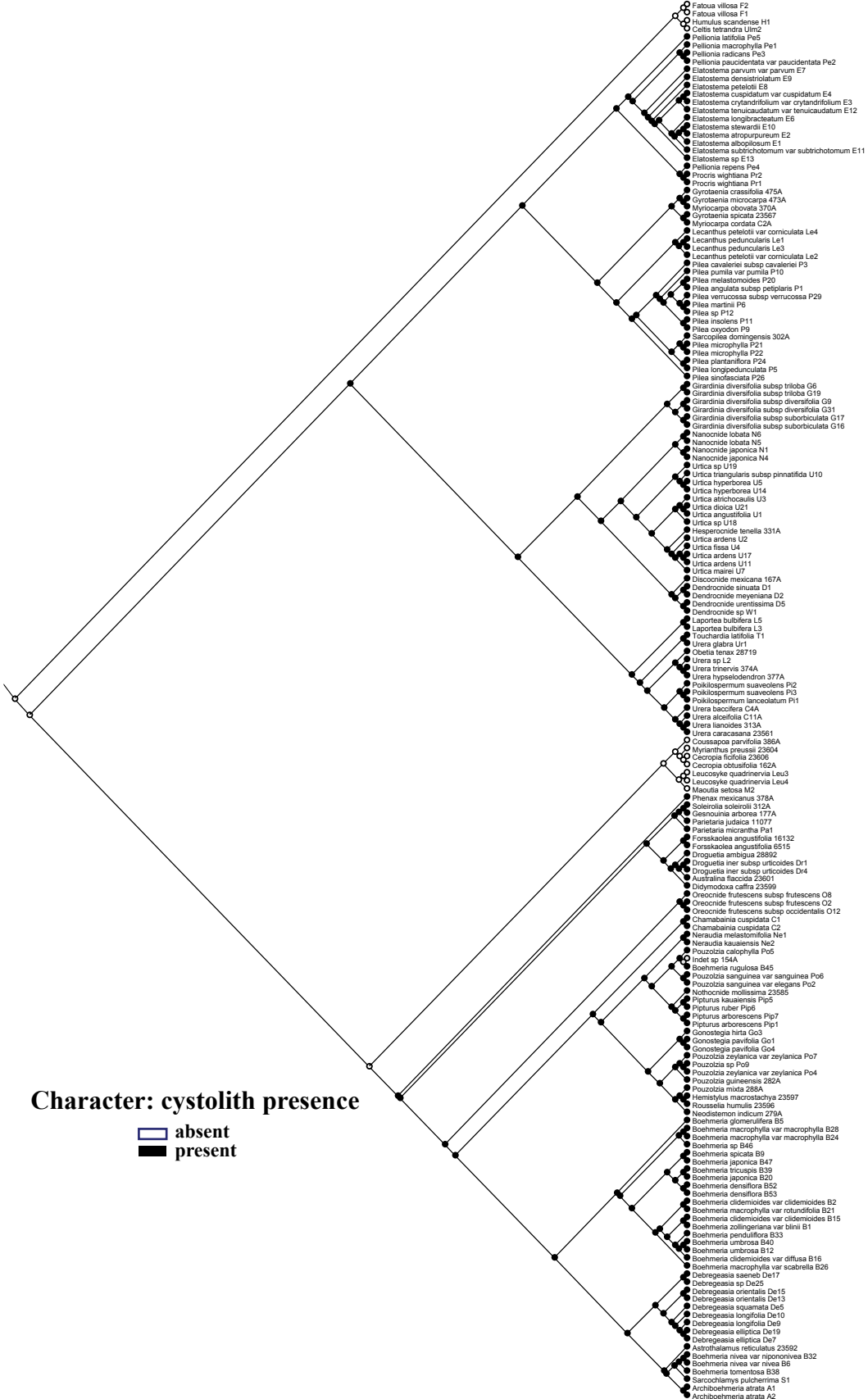

Supplement: S6 Fig — (PDF) [file pone.0141821.s006.pdf]

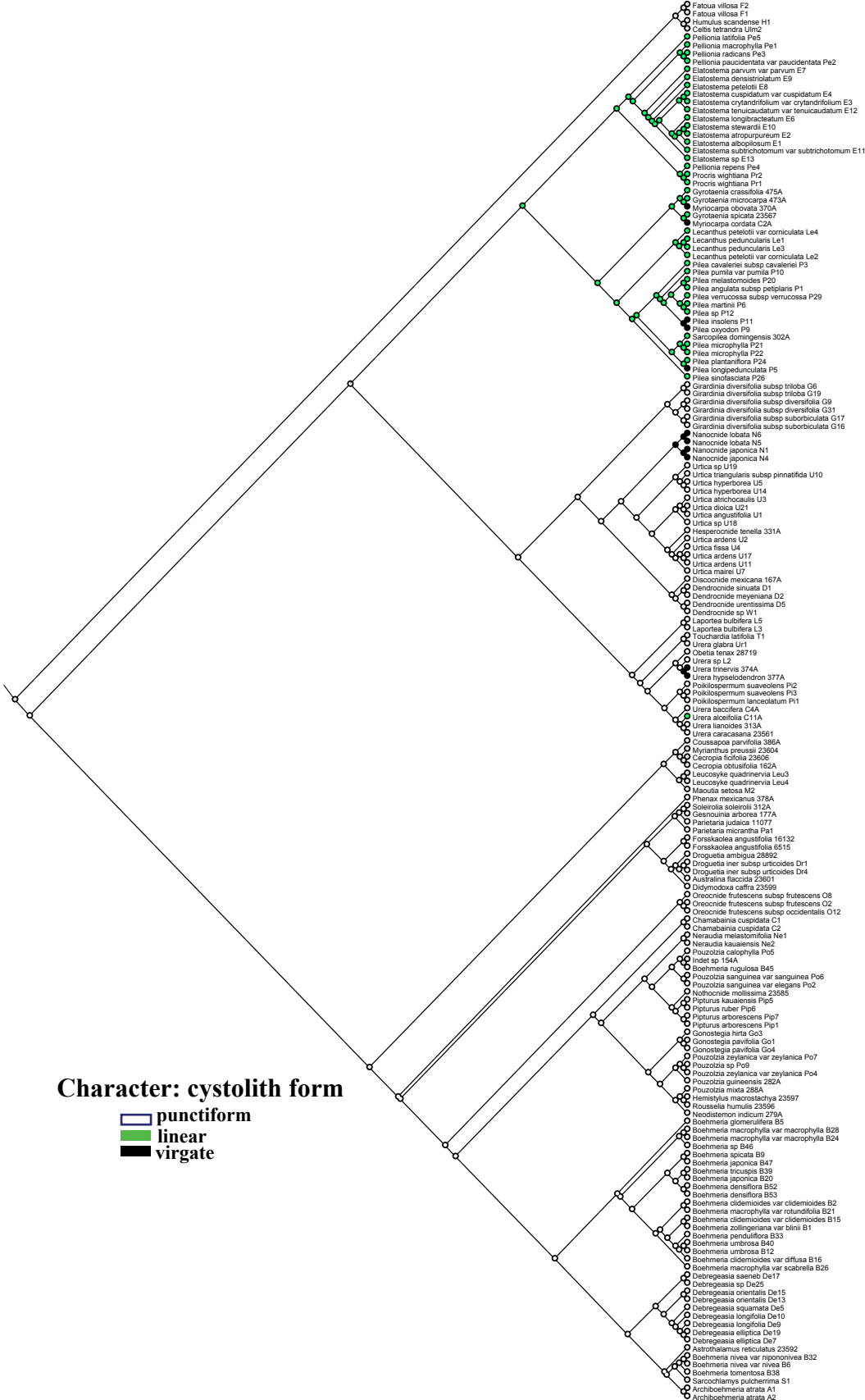

Supplement: S7 Fig — (PDF) [file pone.0141821.s007.pdf]

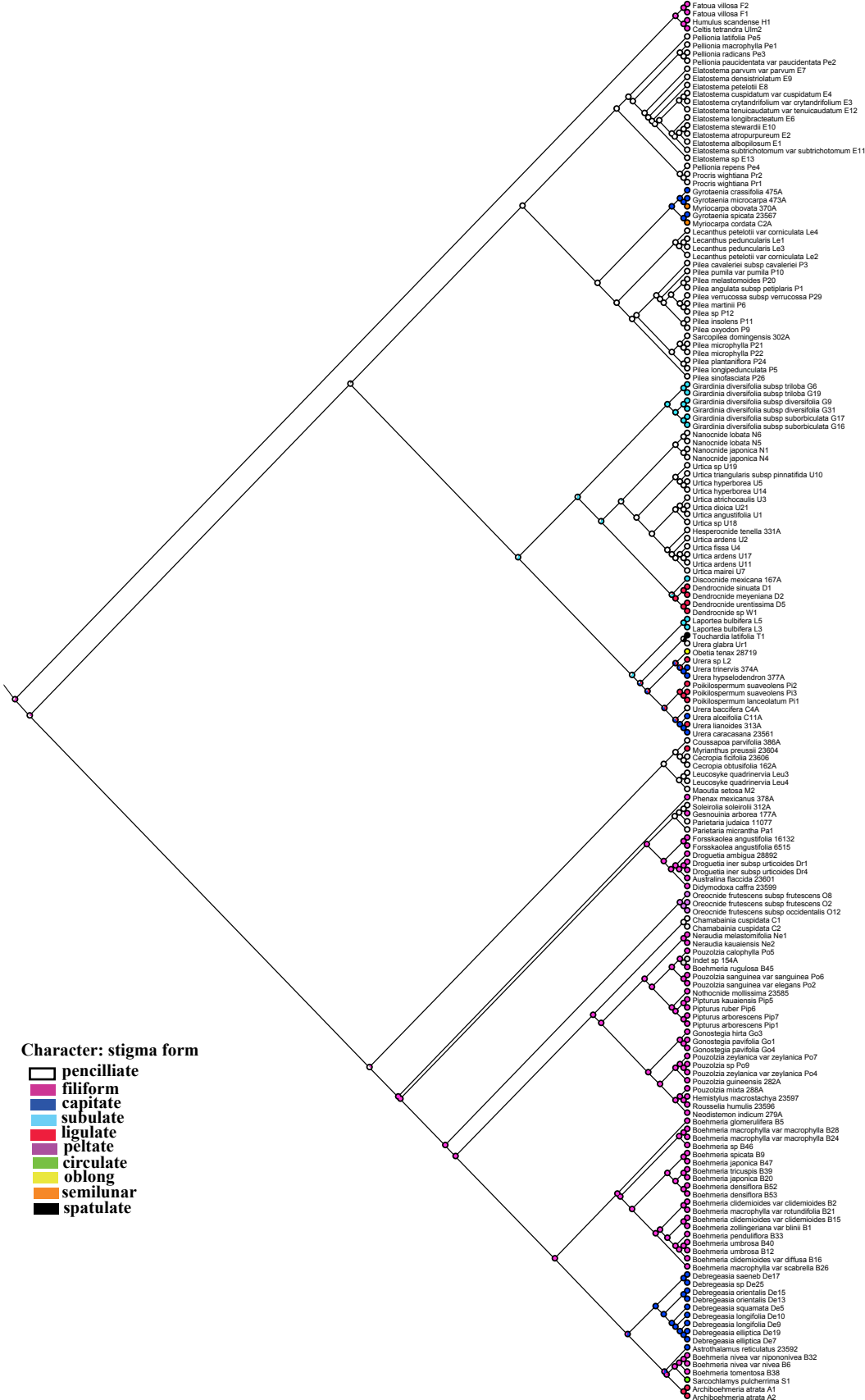

Supplement: S8 Fig — (PDF) [file pone.0141821.s008.pdf]

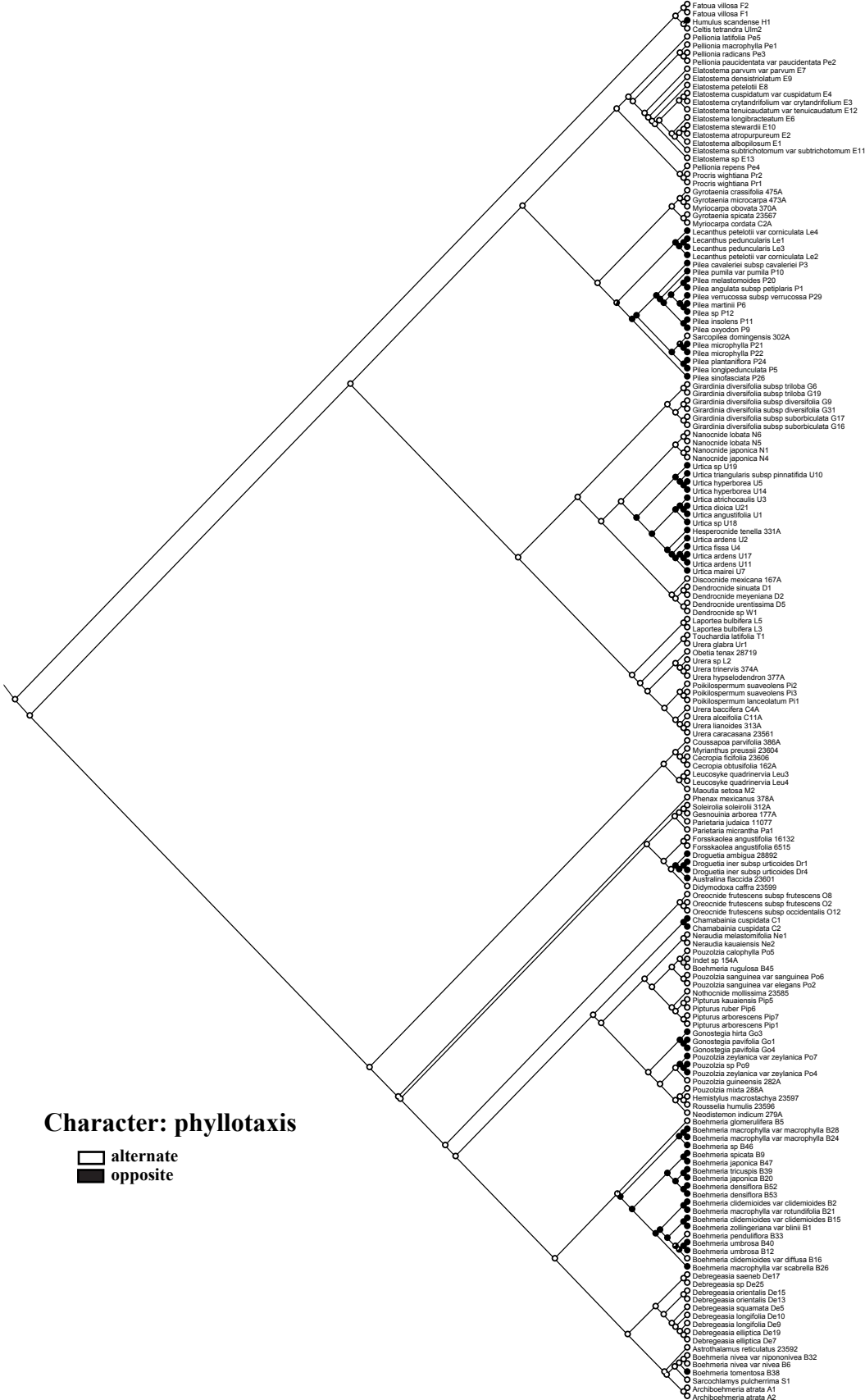

Supplement: S9 Fig — (PDF) [file pone.0141821.s009.pdf]

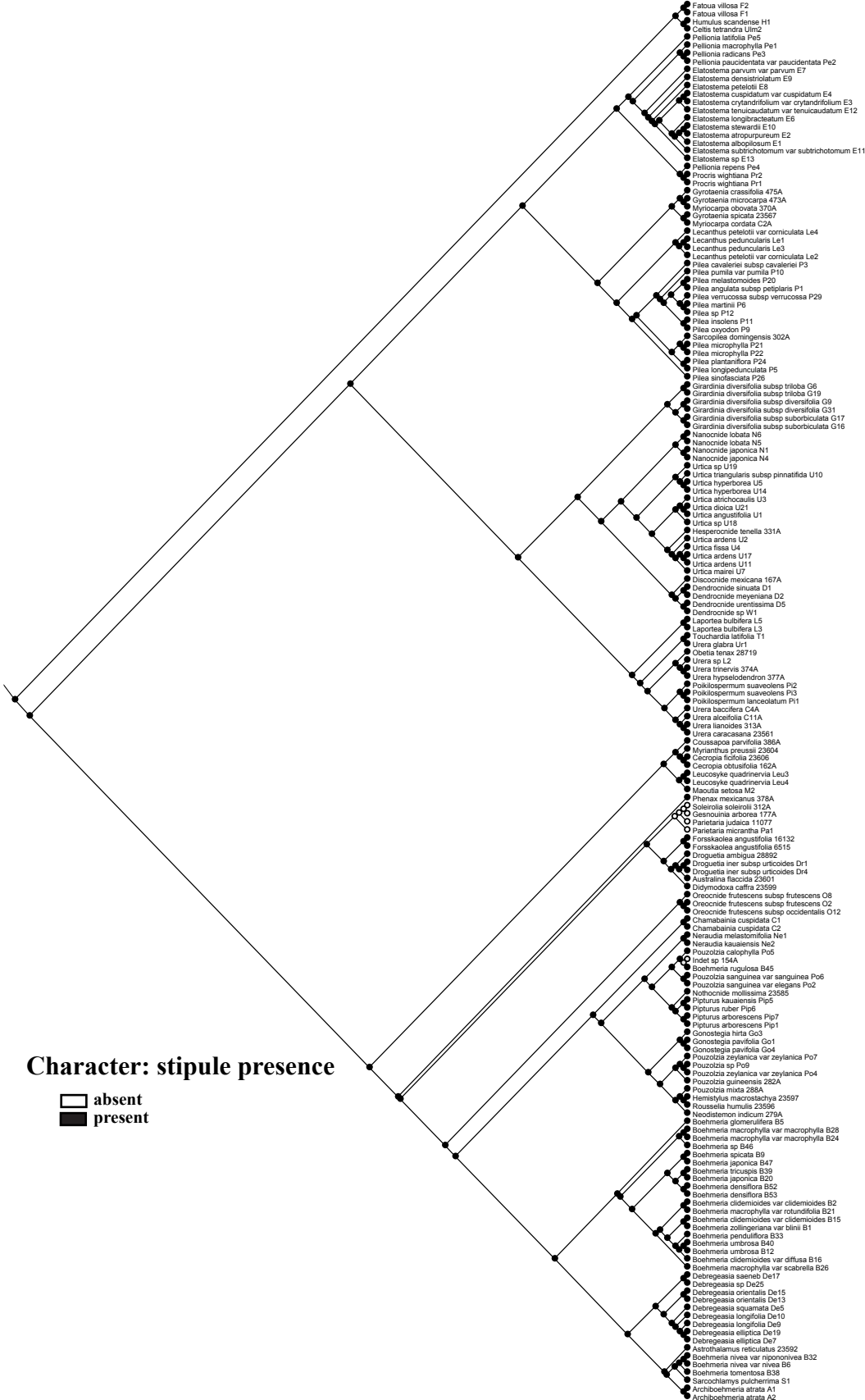

Supplement: S10 Fig — (PDF) [file pone.0141821.s010.pdf]

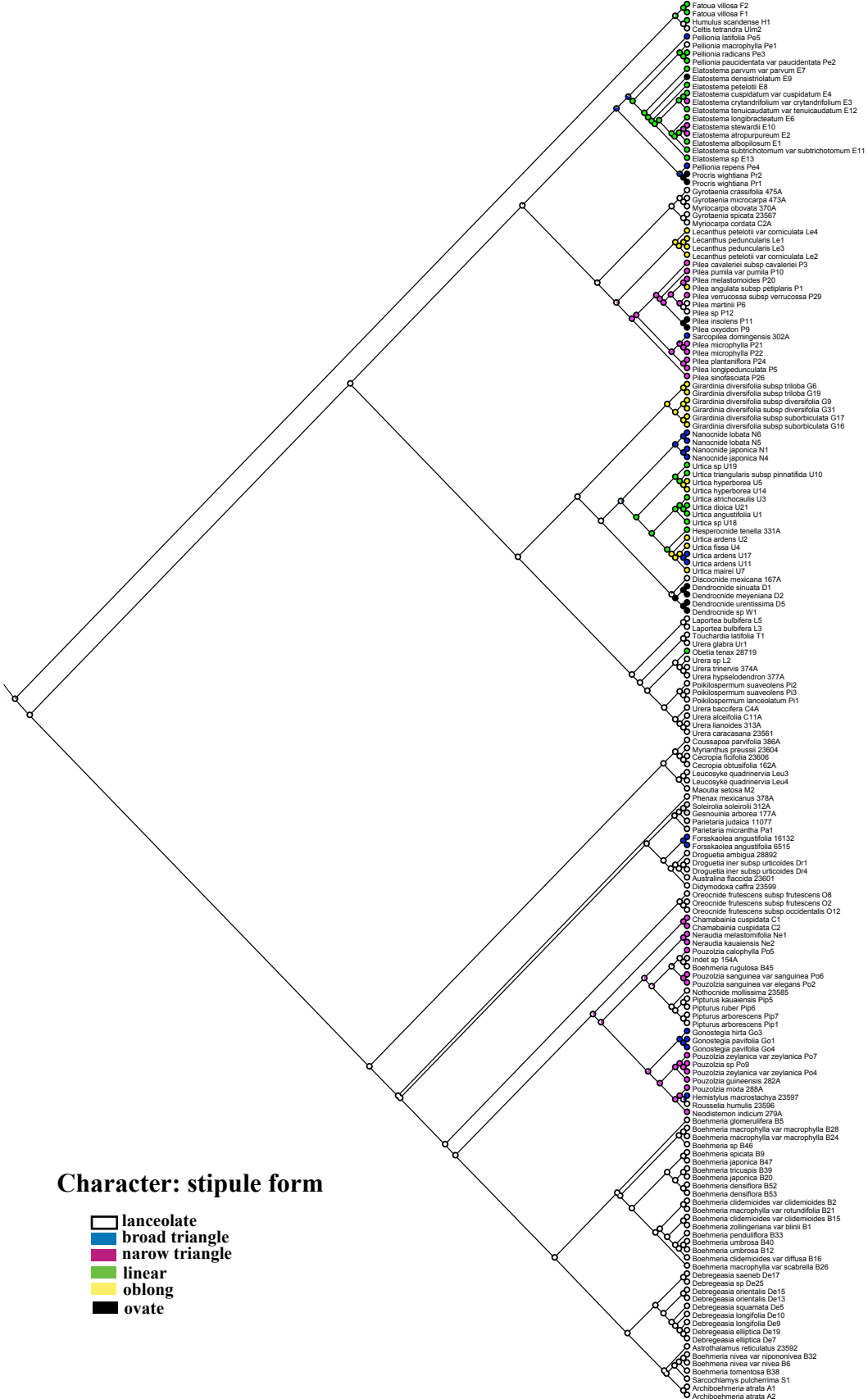

Supplement: S11 Fig — (PDF) [file pone.0141821.s011.pdf]

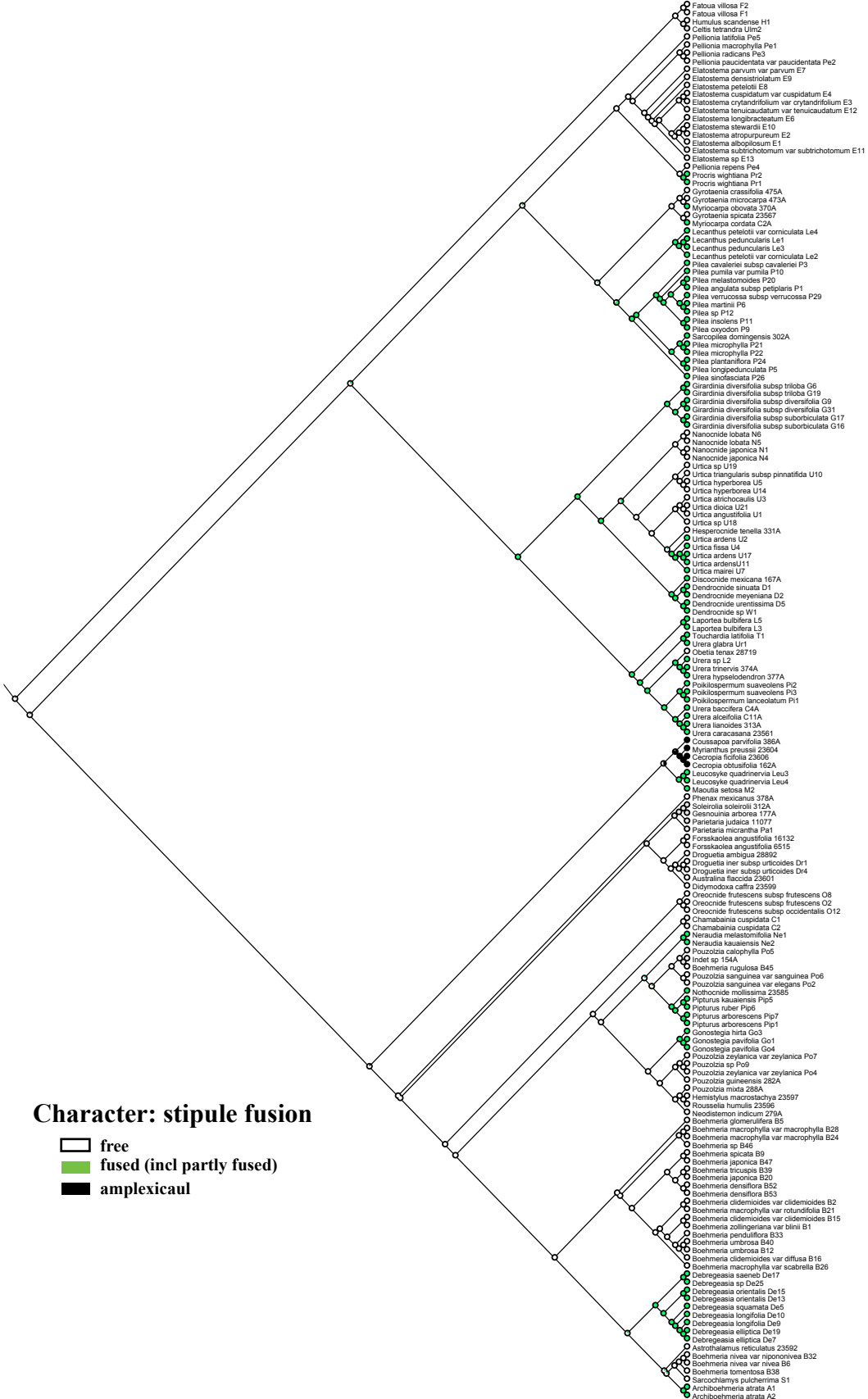

Supplement: S12 Fig — (PDF) [file pone.0141821.s012.pdf]

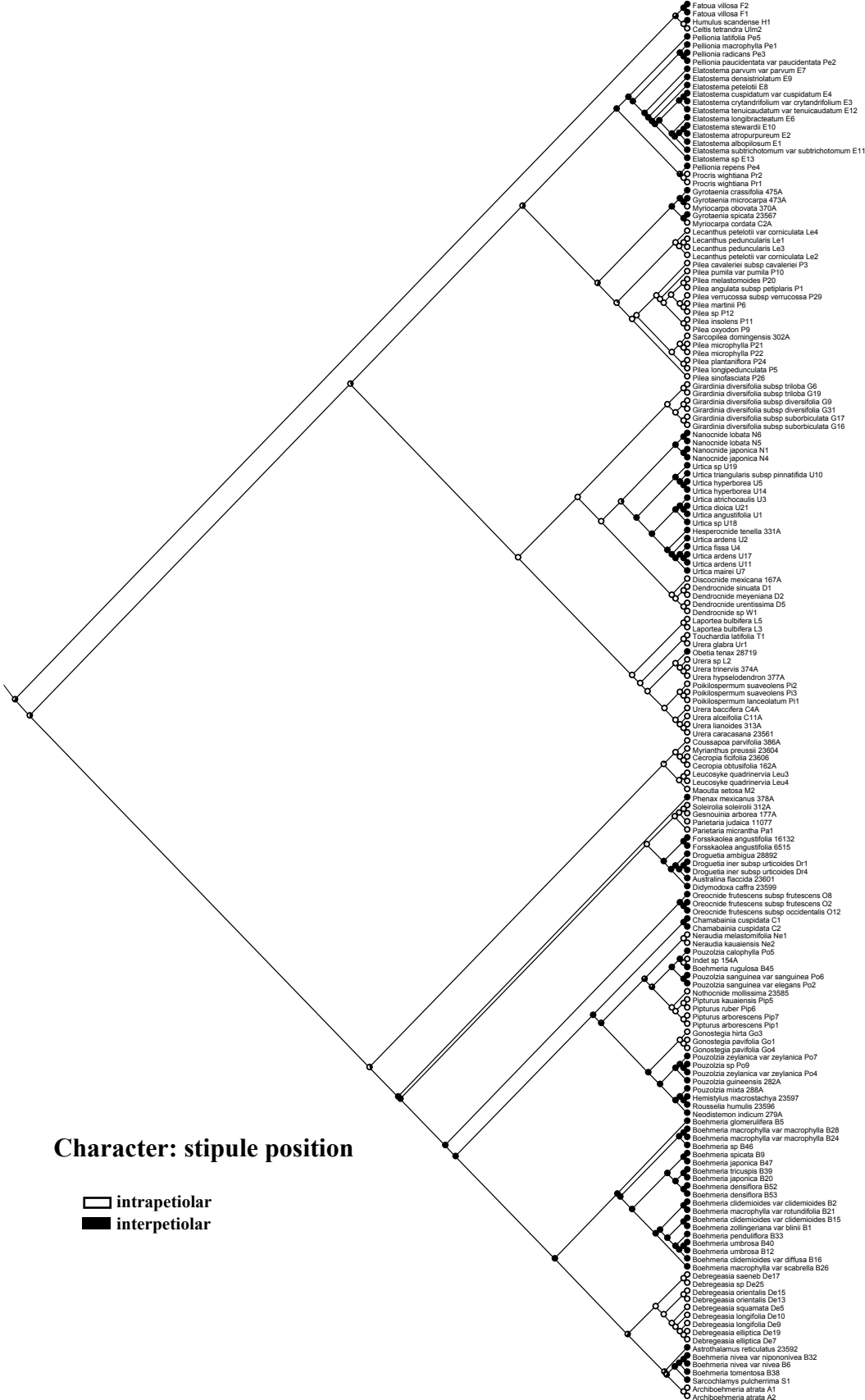

Supplement: S13 Fig — (PDF) [file pone.0141821.s013.pdf]

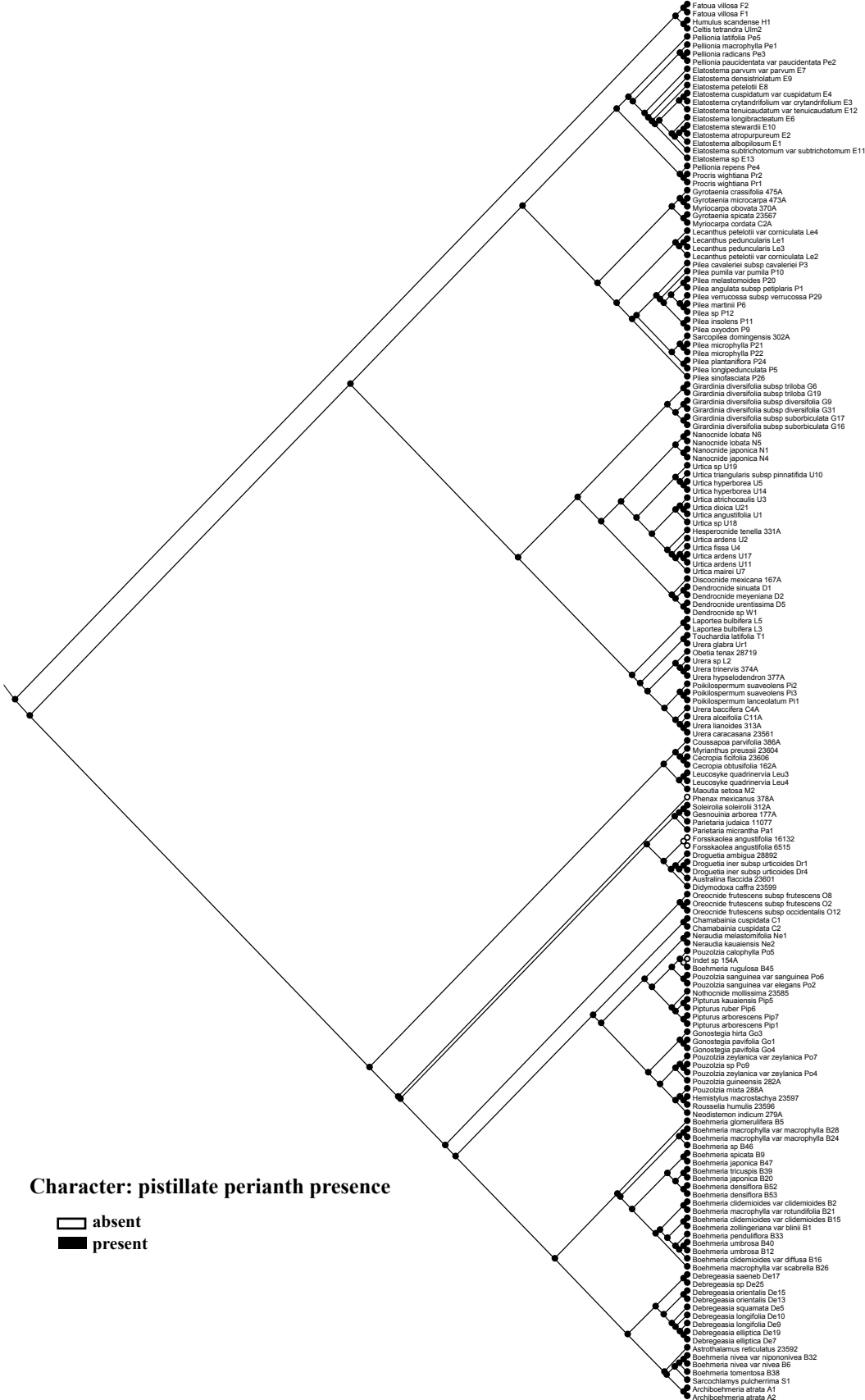

Supplement: S14 Fig — (PDF) [file pone.0141821.s014.pdf]

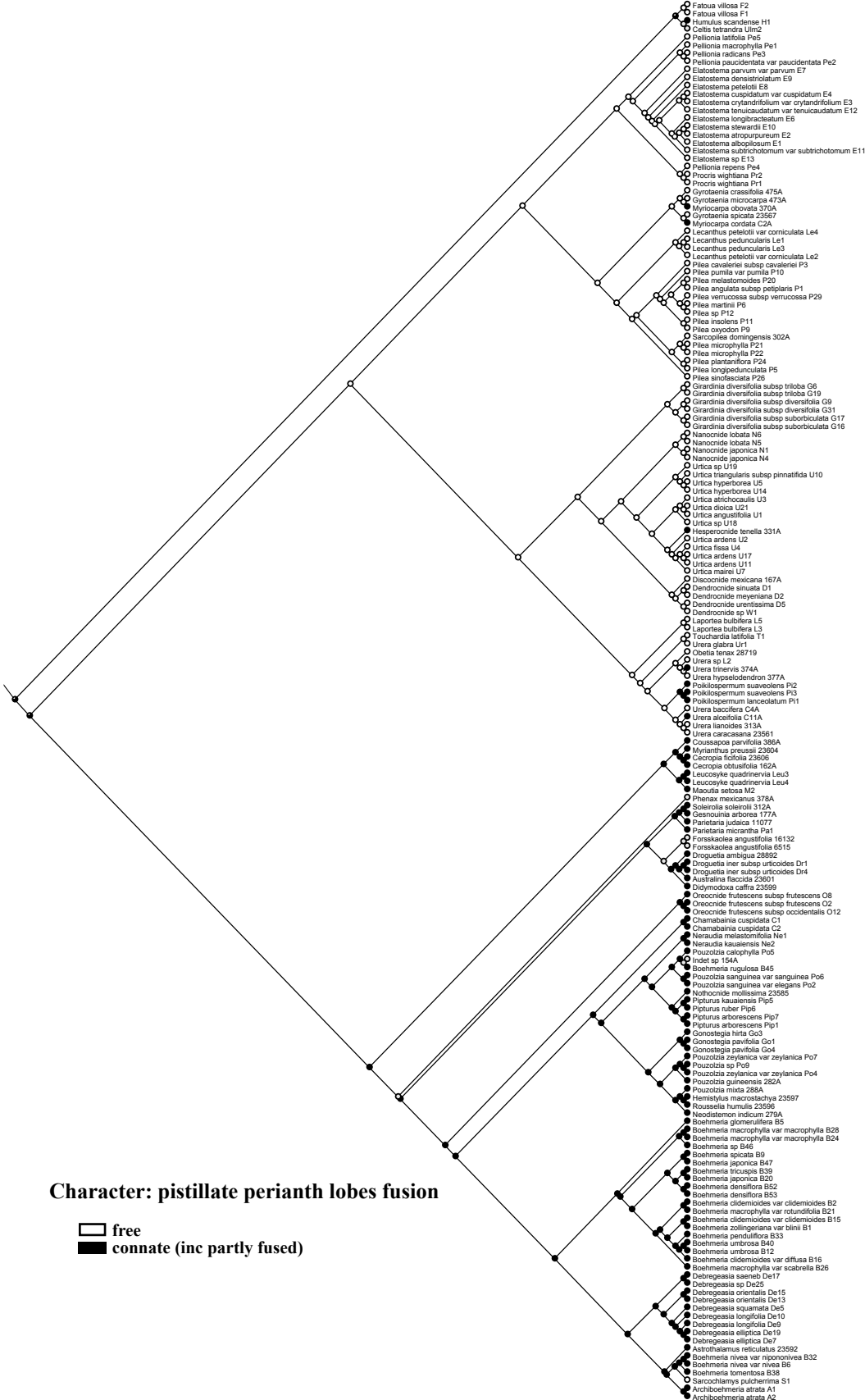

Supplement: S15 Fig — (PDF) [file pone.0141821.s015.pdf]

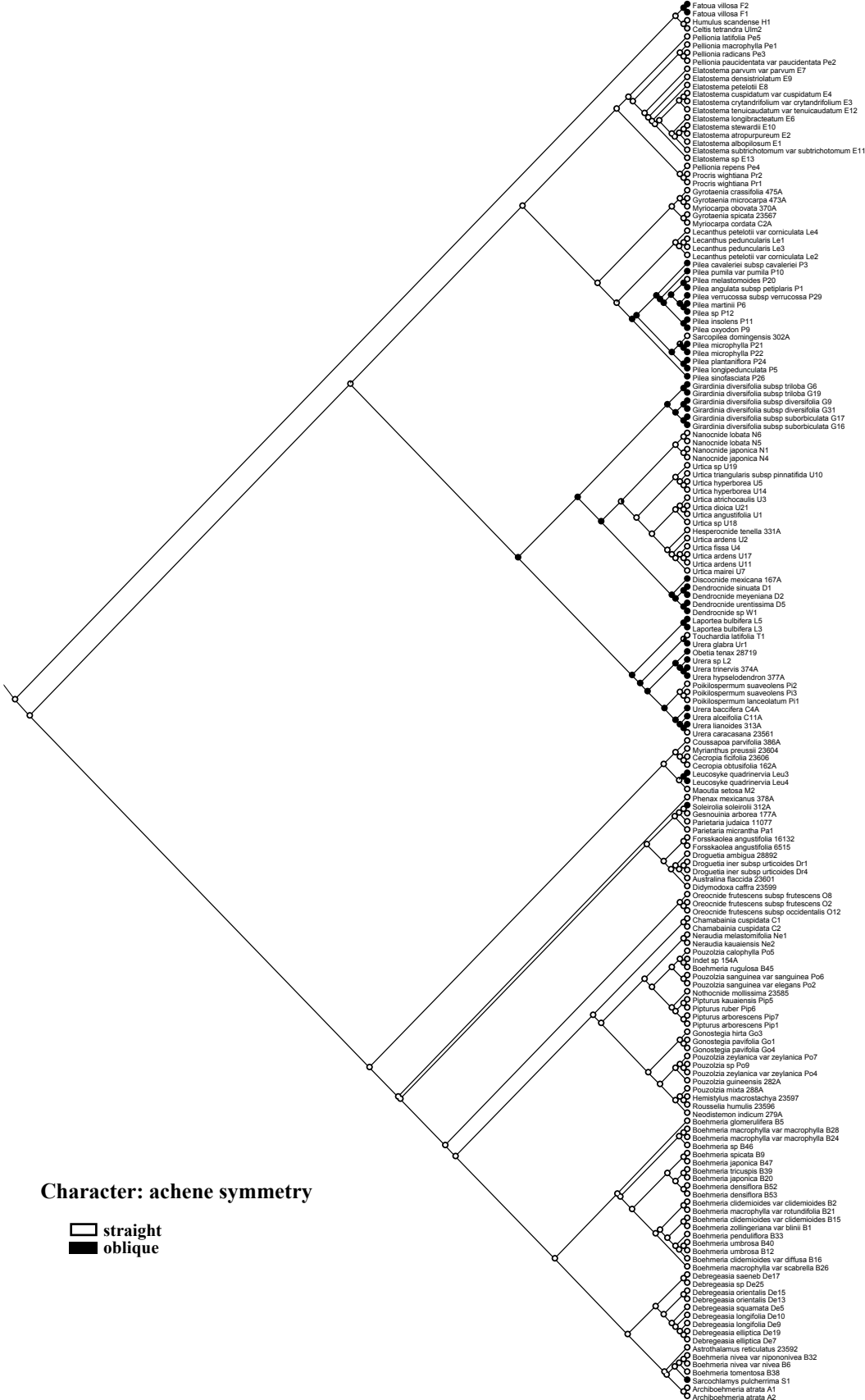

Supplement: S16 Fig — (PDF) [file pone.0141821.s016.pdf]

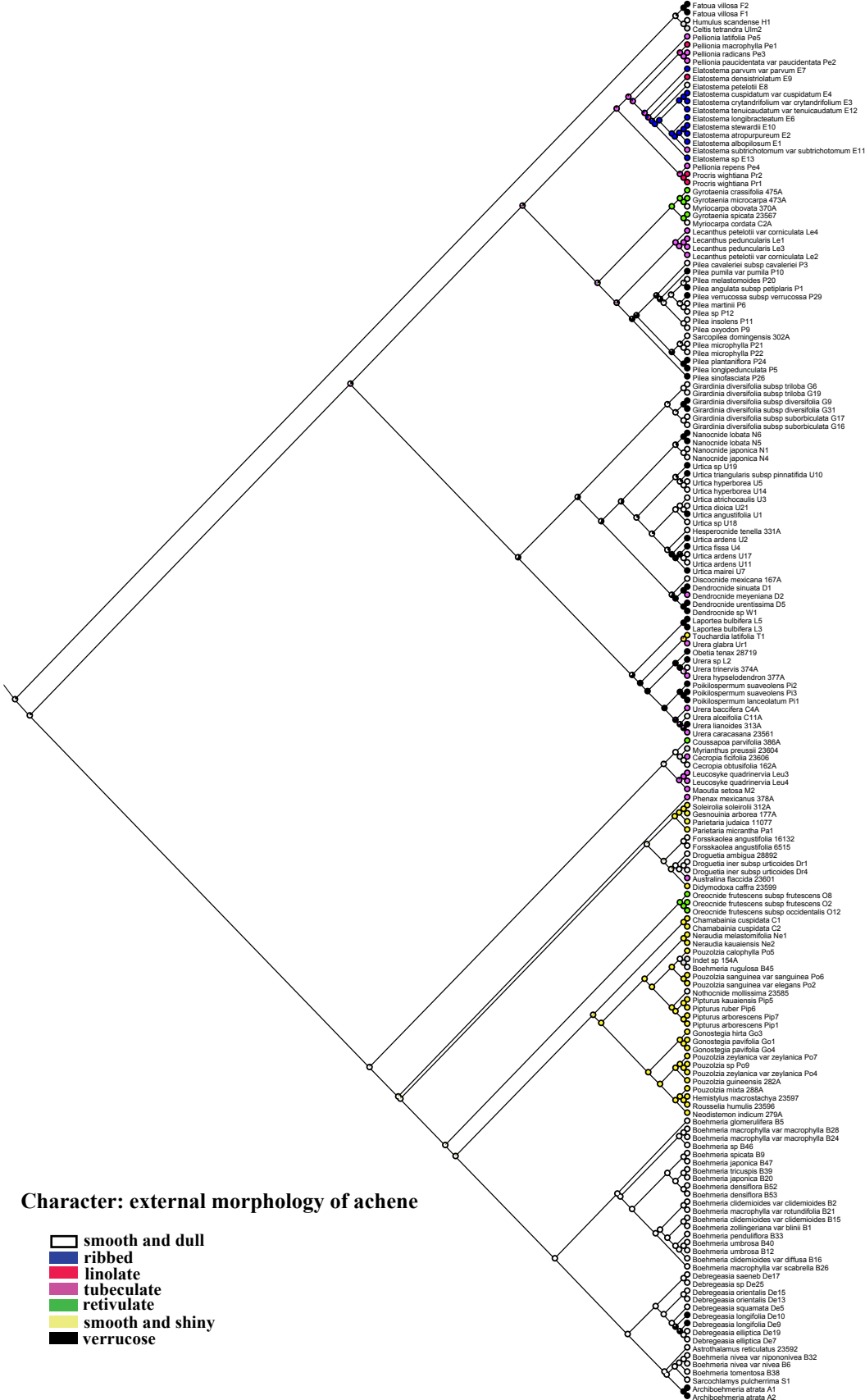

Supplement: S17 Fig — (PDF) [file pone.0141821.s017.pdf]

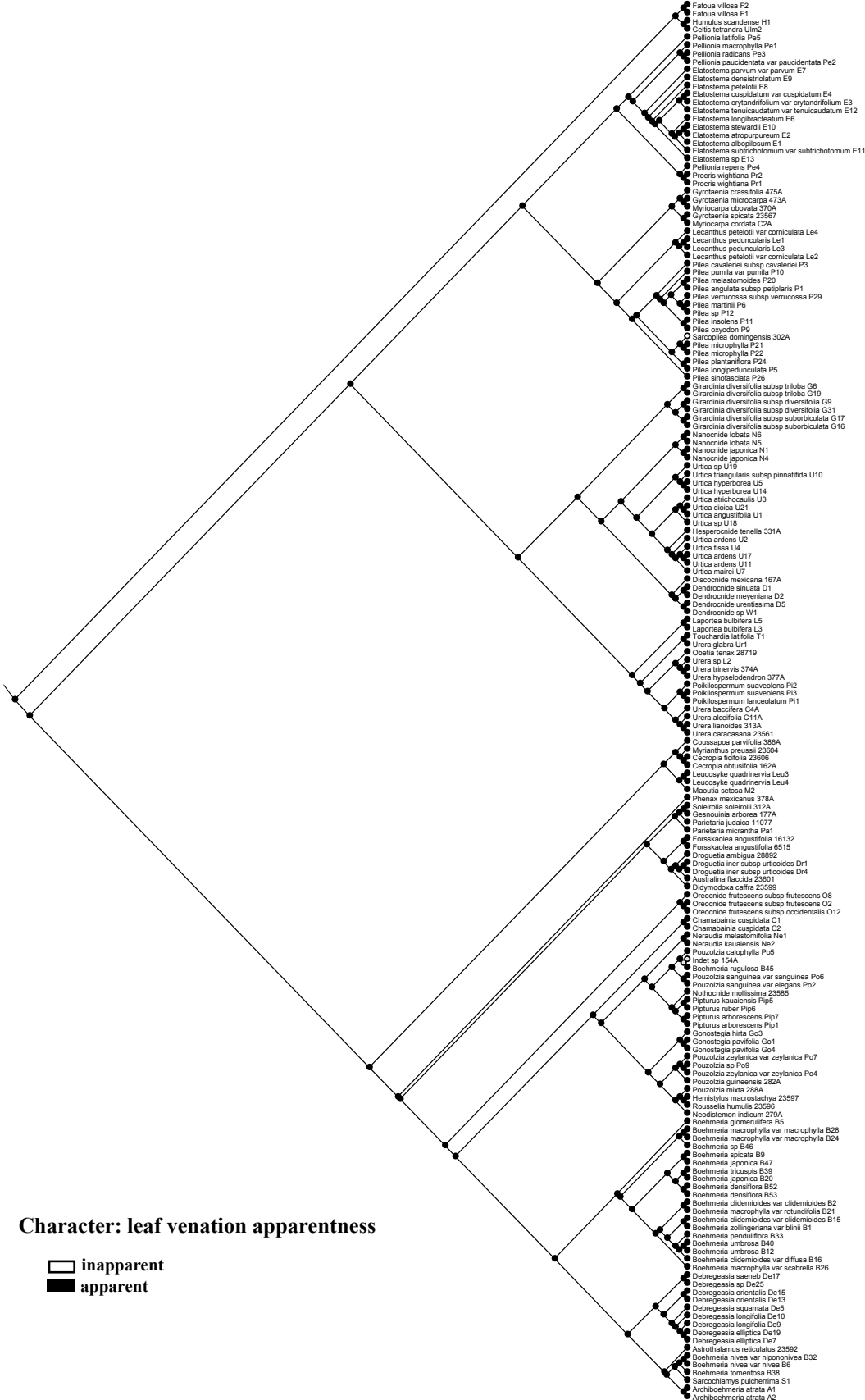

Supplement: S18 Fig — (PDF) [file pone.0141821.s018.pdf]

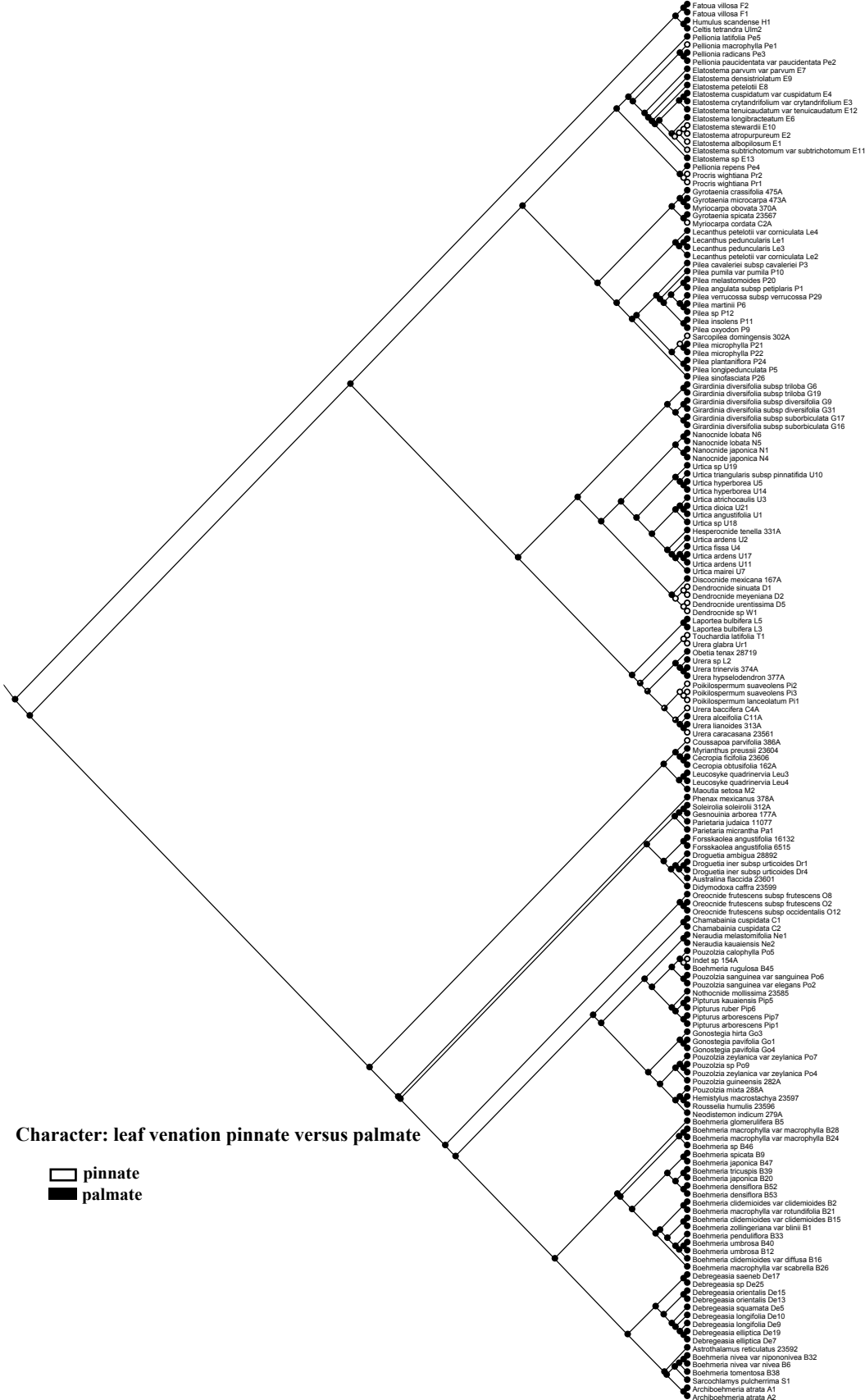

Supplement: S19 Fig — (PDF) [file pone.0141821.s019.pdf]

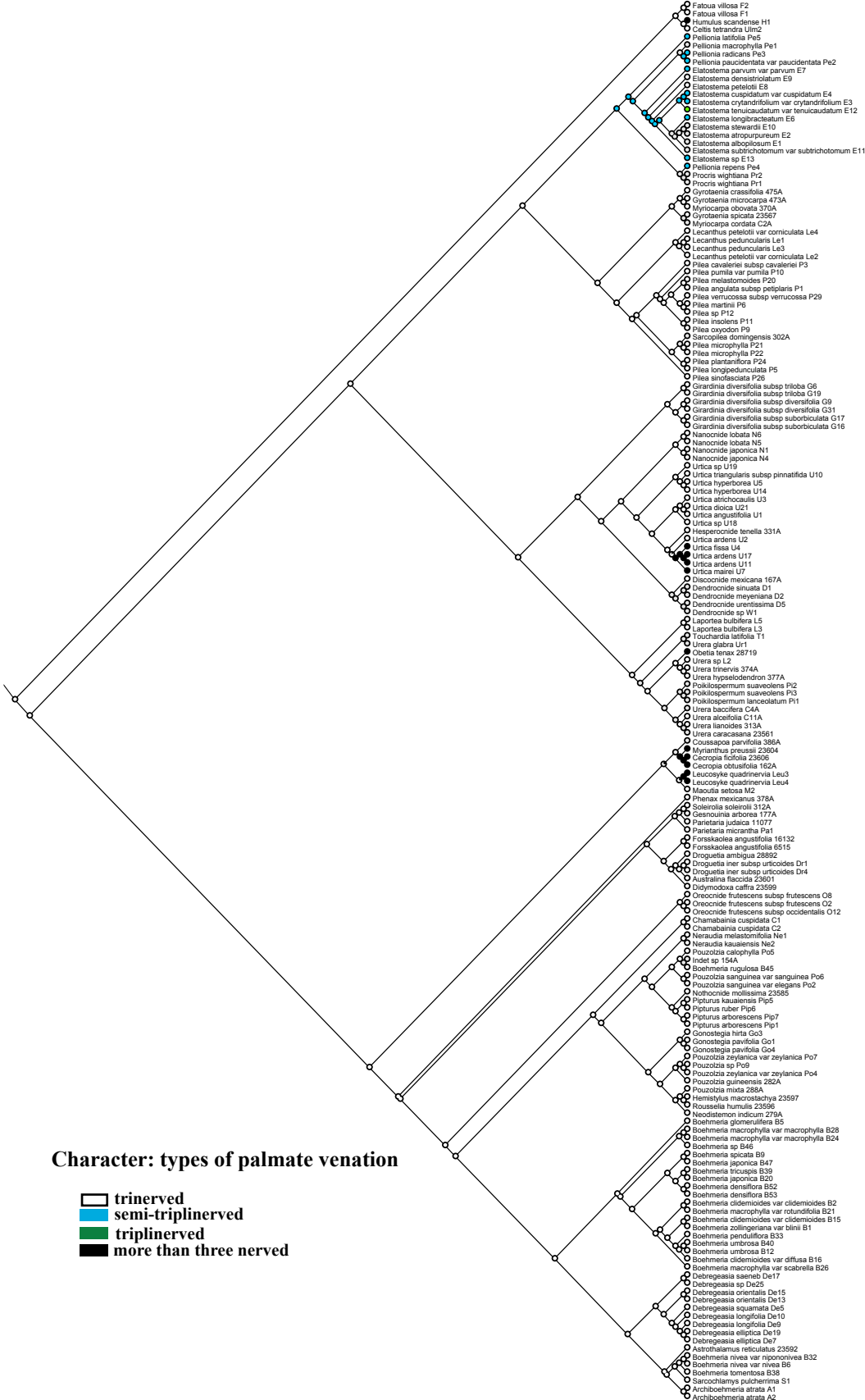

Supplement: S20 Fig — (PDF) [file pone.0141821.s020.pdf]

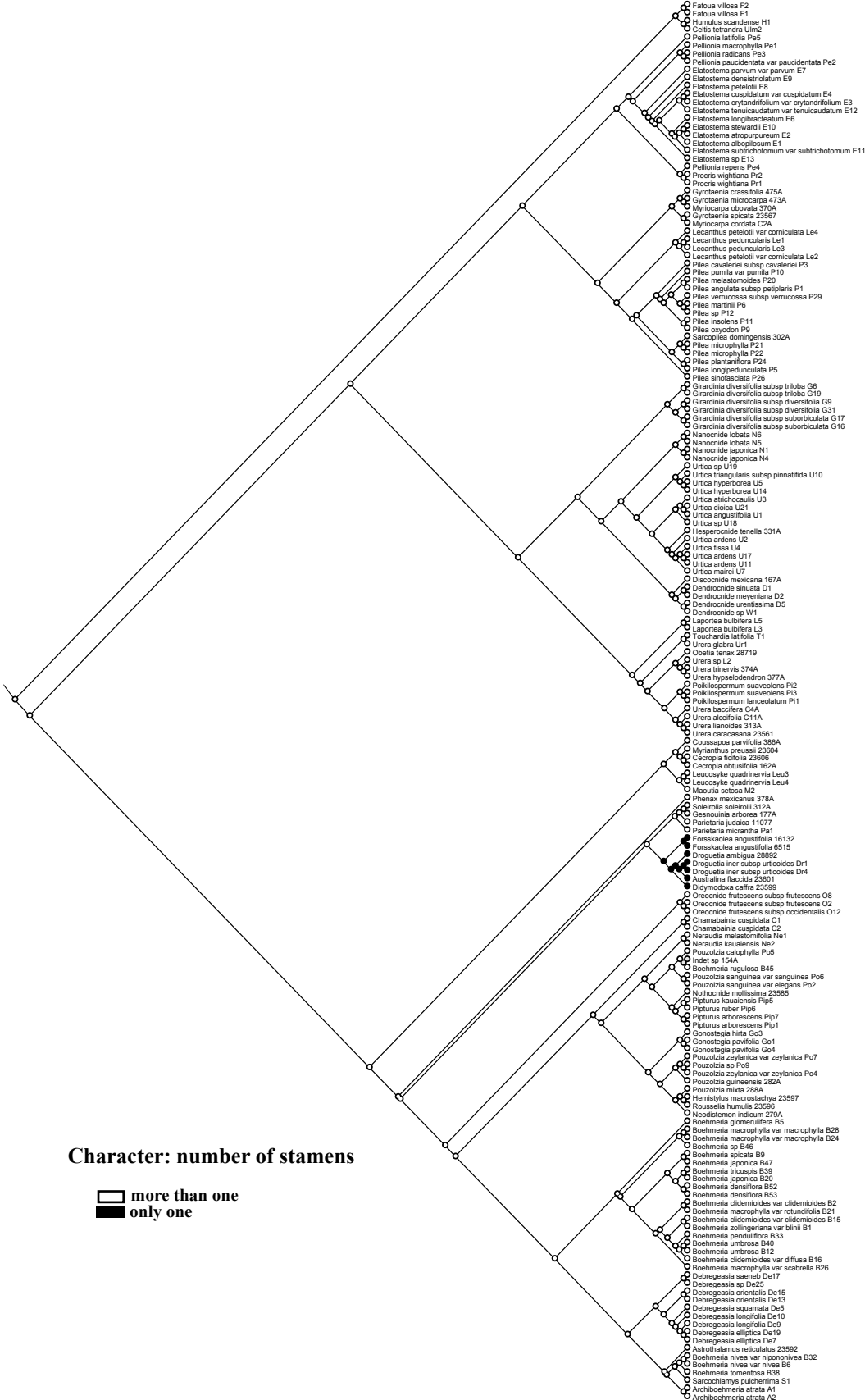

Supplement: S21 Fig — (PDF) [file pone.0141821.s021.pdf]

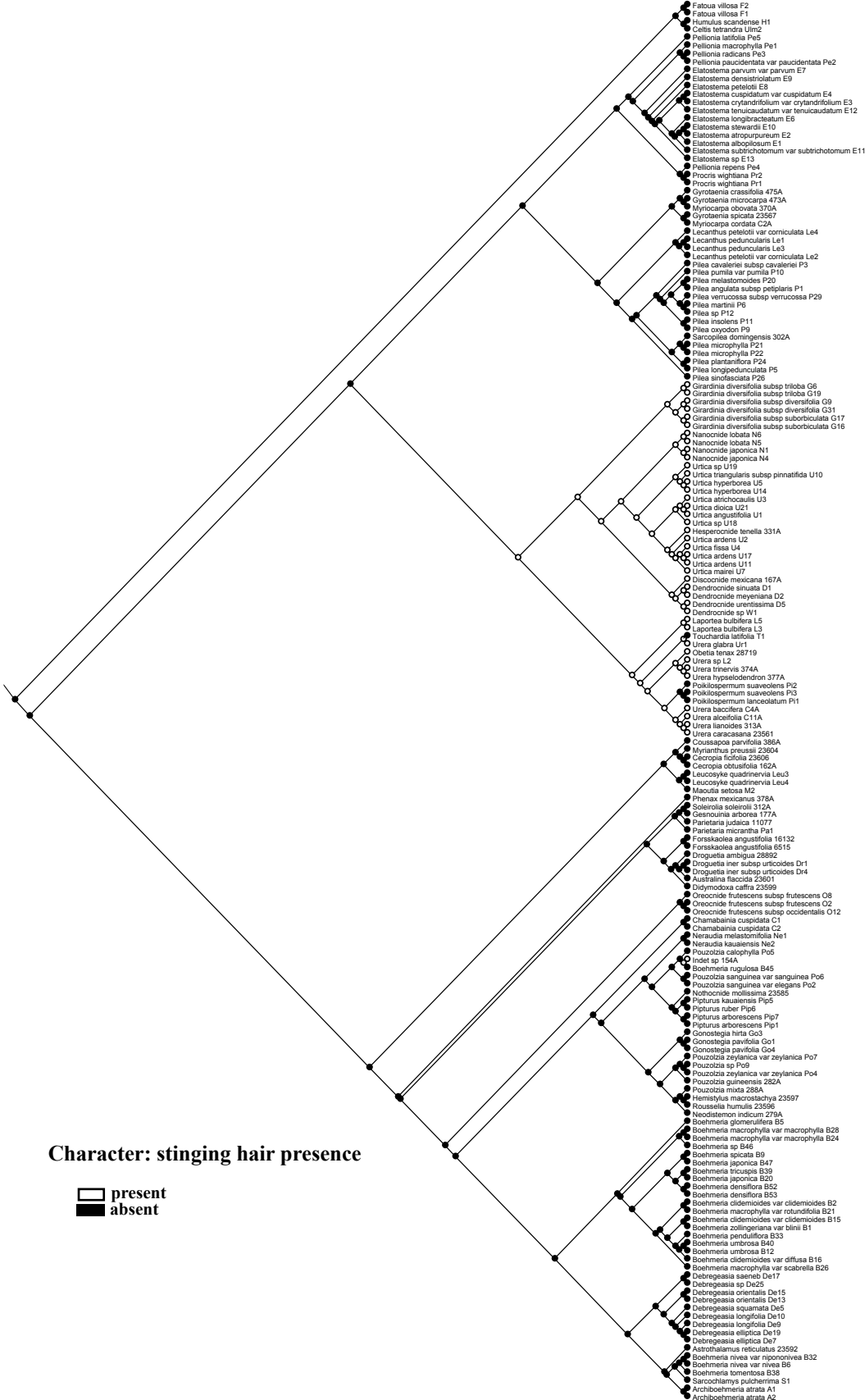

Supplement: S22 Fig — (PDF) [file pone.0141821.s022.pdf]

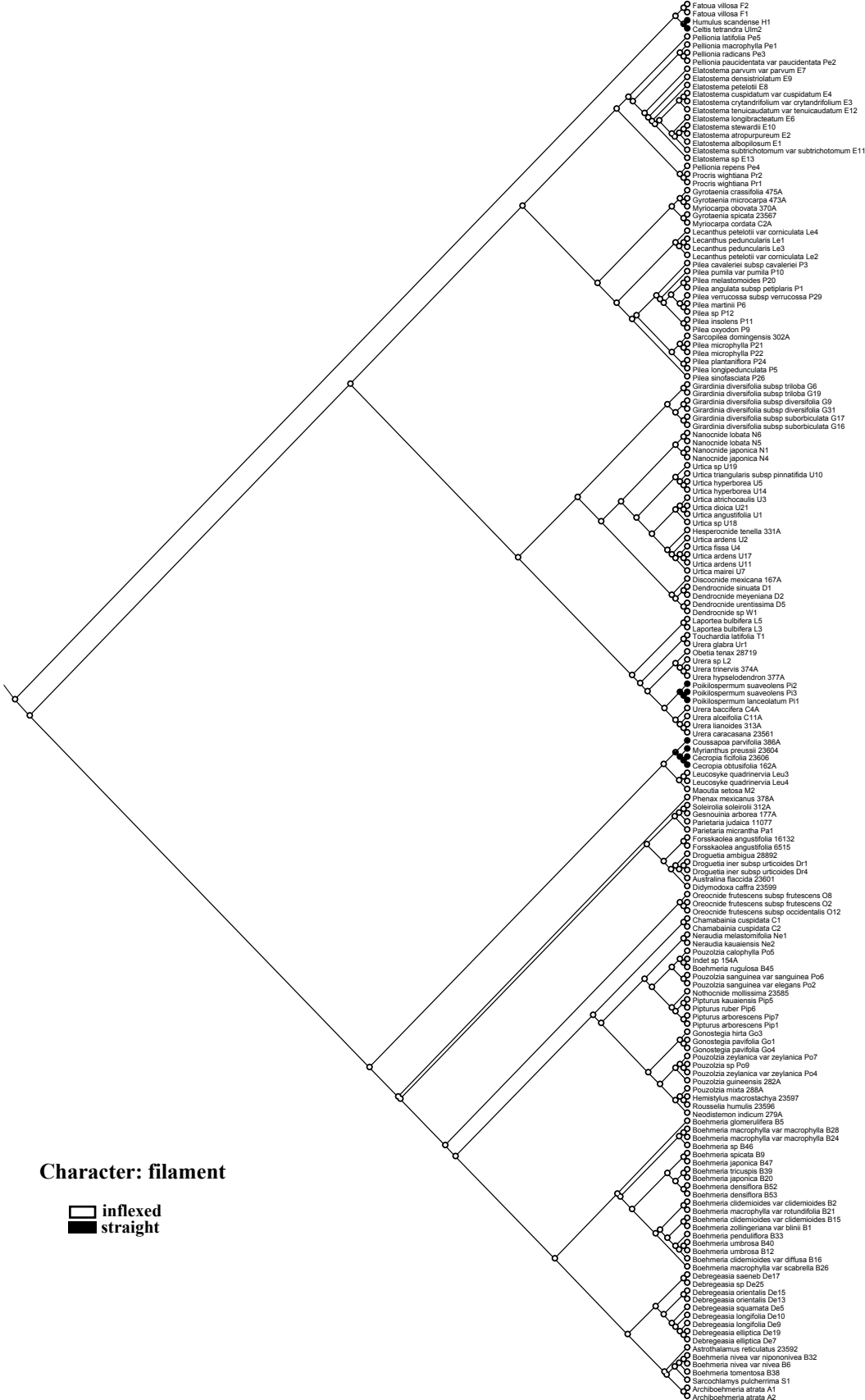

Supplement: S23 Fig — (PDF) [file pone.0141821.s023.pdf]
