# Supplementary material for: Ancestral State Reconstruction Reveals Rampant Homoplasy of Diagnostic Morphological Characters in Urticaceae, Conflicting with Current Classification Schemes
Source: PLoS One. 2015 Nov 3;10(11):e0141821. doi: 10.1371/journal.pone.0141821 (PMC4631448; doi:10.1371/journal.pone.0141821)
Supplement: S4 Fig — (PDF) [file pone.0141821.s004.pdf]

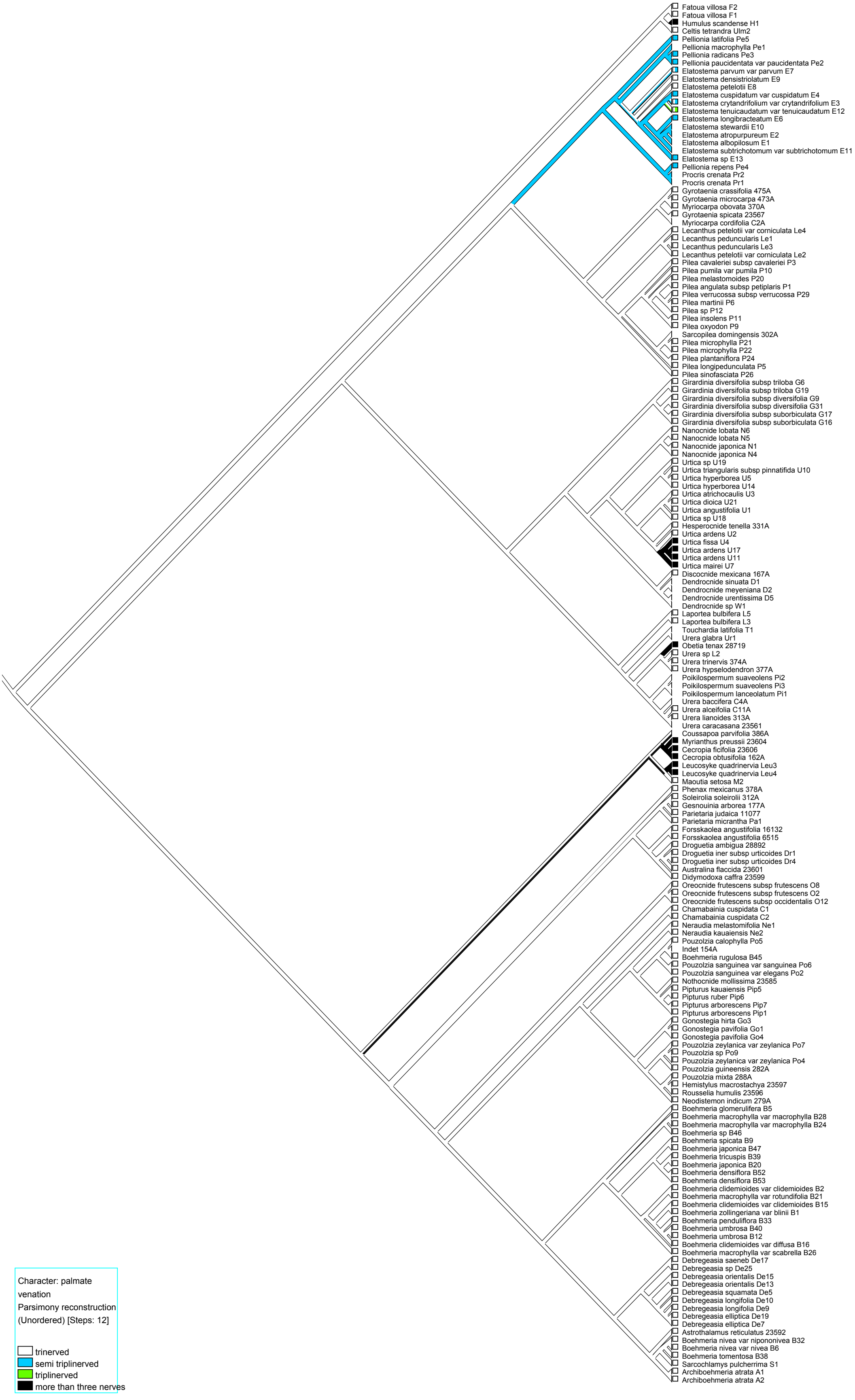
Fatoua villosa F2
Fatoua villosa F1
Humulus scandense H1
Celtis tetrandra Ulm2
Pellionia latifolia Pe5
Pellionia macrophylla Pe1
Pellionia radicans Pe3
Pellionia paucidentata var paucidentata Pe2
Elatostema parvum var parvum E7
Elatostema densistriolatum E9
Elatostema petelotii E8
Elatostema cuspidatum var cuspidatum E4
Elatostema crytandrifolium var crytandrifolium E3
Elatostema tenuicaudatum var tenuicaudatum E12
Elatostema longibracteatum E6
Elatostema stewardii E10
Elatostema atropurpureum E2
Elatostema albopilosum E1
Elatostema subtrichotomum var subtrichotomum E11
Elatostema sp E13
Pellionia repens Pe4
Procris crenata Pr2
Procris crenata Pr1
Gyrotaenia crassifolia 475A
Gyrotaenia microcarpa 473A
Myriocarpa obovata 370A
Gyrotaenia spicata 23567
Myriocarpa cordifolia C2A
Lecanthus petelotii var corniculata Le4
Lecanthus peduncularis Le1
Lecanthus peduncularis Le3
Lecanthus petelotii var corniculata Le2
Pilea cavaleriei subsp cavaleriei P3
Pilea pumila var pumila P10
Pilea melastomoides P20
Pilea angulata subsp petiplaris P1
Pilea verrucossa subsp verrucossa P29
Pilea martinii P6
Pilea sp P12
Pilea insolens P11
Pilea oxyodon P9
Sarcopilea domingensis 302A
Pilea microphylla P21
Pilea microphylla P22
Pilea plantaniflora P24
Pilea longipedunculata P5
Pilea sinofasciata P26
Girardinia diversifolia subsp triloba G6
Girardinia diversifolia subsp triloba G19
Girardinia diversifolia subsp diversifolia G9
Girardinia diversifolia subsp diversifolia G31
Girardinia diversifolia subsp suborbiculata G17
Girardinia diversifolia subsp suborbiculata G16
Nanocnide lobata N6
Nanocnide lobata N5
Nanocnide japonica N1
Nanocnide japonica N4
Urtica sp U19
Urtica triangularis subsp pinnatifida U10
Urtica hyperborea U5
Urtica hyperborea U14
Urtica atrichocaulis U3
Urtica dioica U21
Urtica angustifolia U1
Urtica sp U18
Hesperocnide tenella 331A
Urtica ardens U2
Urtica fissa U4
Urtica ardens U17
Urtica ardens U11
Urtica mairei U7
Discocnide mexicana 167A
Dendrocnide sinuata D1
Dendrocnide meyeniana D2
Dendrocnide urentissima D5
Dendrocnide sp W1
Laportea bulbifera L5
Laportea bulbifera L3
Touchardia latifolia T1
Urera glabra Ur1
Obetia tenax 28719
Urera sp L2
Urera trinervis 374A
Urera hypselodendron 377A
Poikilospermum suaveolens Pi2
Poikilospermum suaveolens Pi3
Poikilospermum lanceolatum Pi1
Urera baccifera C4A
Urera alceifolia C11A
Urera lianoides 313A
Urera caracasana 23561
Coussapoa parvifolia 386A
Myrianthus preussii 23604
Cecropia ficifolia 23606
Cecropia obtusifolia 162A
Leucosyke quadrinervia Leu3
Leucosyke quadrinervia Leu4
Maoutia setosa M2
Phenax mexicanus 378A
Soleirolia soleirolii 312A
Gesnouinia arborea 177A
Parietaria judaica 11077
Parietaria micrantha Pa1
Forsskaolea angustifolia 16132
Forsskaolea angustifolia 6515
Droguetia ambigua 28892
Droguetia iner subsp urticoides Dr1
Droguetia iner subsp urticoides Dr4
Australina flaccida 23601
Didymodoxa caffra 23599
Oreocnide frutescens subsp frutescens O8
Oreocnide frutescens subsp frutescens O2
Oreocnide frutescens subsp occidentalis O12
Chamabainia cuspidata C1
Chamabainia cuspidata C2
Neraudia melastomifolia Ne1
Neraudia kauaiensis Ne2
Pouzolzia calophylla Po5
Indet 154A
Boehmeria rugulosa B45
Pouzolzia sanguinea var sanguinea Po6
Pouzolzia sanguinea var elegans Po2
Nothocnide mollissima 23585
Pipturus kauaiensis Pip5
Pipturus ruber Pip6
Pipturus arborescens Pip7
Pipturus arborescens Pip1
Gonostegia hirta Go3
Gonostegia pavifolia Go1
Gonostegia pavifolia Go4
Pouzolzia zeylanica var zeylanica Po7
Pouzolzia sp Po9
Pouzolzia zeylanica var zeylanica Po4
Pouzolzia guineensis 282A
Pouzolzia mixta 288A
Hemistylus macrostachya 23597
Rousselia humulis 23596
Neodistemon indicum 279A
Boehmeria glomerulifera B5
Boehmeria macrophylla var macrophylla B28
Boehmeria macrophylla var macrophylla B24
Boehmeria sp B46
Boehmeria spicata B9
Boehmeria japonica B47
Boehmeria tricuspis B39
Boehmeria japonica B20
Boehmeria densiflora B52
Boehmeria densiflora B53
Boehmeria clidemioides var clidemioides B2
Boehmeria macrophylla var rotundifolia B21
Boehmeria clidemioides var clidemioides B15
Boehmeria zollingeriana var blinii B1
Boehmeria penduliflora B33
Boehmeria umbrosa B40
Boehmeria umbrosa B12
Boehmeria clidemioides var diffusa B16
Boehmeria macrophylla var scabrella B26
Debregeasia saeneb De17
Debregeasia sp De25
Debregeasia orientalis De15
Debregeasia orientalis De13
Debregeasia squamata De5
Debregeasia longifolia De10
Debregeasia longifolia De9
Debregeasia elliptica De19
Debregeasia elliptica De7
Astrothalamus reticulatus 23592
Boehmeria nivea var niponinivea B32
Boehmeria nivea var nivea B6
Boehmeria tomentosa B38
Sarcochlamys pulcherrima S1
Archiboehmeria atrata A1
Archiboehmeria atrata A2
Character: palmate venation
Parsimony reconstruction (Unordered) [Steps: 12]
trinerved
semi triplinerved
triplinerved
more than three nerves
