# Supplementary material for: Ancestral State Reconstruction Reveals Rampant Homoplasy of Diagnostic Morphological Characters in Urticaceae, Conflicting with Current Classification Schemes
Source: PLoS One. 2015 Nov 3;10(11):e0141821. doi: 10.1371/journal.pone.0141821 (PMC4631448; doi:10.1371/journal.pone.0141821)
Supplement: S1 Table — (DOCX) [file pone.0141821.s024.docx]

| **Table S1. Minimum number of origins for characters states examined, within Urticaceae.** | | | | | | | | | | | | | | | | | | | | | | |
| --- | --- | --- | --- | --- | --- | --- | --- | --- | --- | --- | --- | --- | --- | --- | --- | --- | --- | --- | --- | --- | --- | --- |
|  |  |  |  |  |  |  |  |  |  |  |  |  |  |  |  |  |  |  |  |  |  |  |
|  |  | **TOTAL** | State 1 |  | State 2 |  | State 3 |  | State 4 |  | State 5 |  | State 6 |  | State 7 |  | State 8 |  | State 9 |  | State 10 |  |
| **1** | Habit | **12** | herbaceous** | 5 | woody | 7 |  |  |  |  |  |  |  |  |  |  |  |  |  |  |  |  |
| **2** | Cystolith presence | **1** | absent* | 0 | present* | 0 |  |  |  |  |  |  |  |  |  |  |  |  |  |  |  |  |
| **3** | Cystolith form | **16** | punctiform** | 2 | linear | 2 | virgate | 12 |  |  |  |  |  |  |  |  |  |  |  |  |  |  |
| **4** | Stigma form | **29** | penicillate* | 2 | filiform* | 1 | capitate | 3 | subulate | 3 | ligulate | 6 | peltate | 5 | circular |  | oblong | 1 | semilunar |  | spatulate | 1 |
| **5** | Phyllotaxis | **12** | alternate** | 3 | opposite | 9 |  |  |  |  |  |  |  |  |  |  |  |  |  |  |  |  |
| **6** | Stipule presence | **1** | absent | 1 | present** | 0 |  |  |  |  |  |  |  |  |  |  |  |  |  |  |  |  |
| **7** | Stipule form | **28** | lanceolate** | 5 | broad triangle | 7 | narrow triangle | 4 | linear | 3 | oblong | 5 | ovate | 4 |  |  |  |  |  |  |  |  |
| **8** | Stipule fusion | **15** | free** | 4 | fused (incl partly fused) | 11 |  |  |  |  |  |  |  |  |  |  |  |  |  |  |  |  |
| **9** | Stipule position | **12** | intrapetiolar | 9 | interpetiolar** | 3 |  |  |  |  |  |  |  |  |  |  |  |  |  |  |  |  |
| 10 | Pistillate perianth presence | **2** | absent | 2 | present** | 0 |  |  |  |  |  |  |  |  |  |  |  |  |  |  |  |  |
| 11 | Pistillate perianth lobes fusion | **7** | free* | 1 | connate (incl partly fused) * | 6 |  |  |  |  |  |  |  |  |  |  |  |  |  |  |  |  |
| 12 | Achene symmetry | **11** | straight** | 6 | oblique | 5 |  |  |  |  |  |  |  |  |  |  |  |  |  |  |  |  |
| 13 | External morphology of achene | **40** | smooth-and-dull* | 24 | ribbed | 1 | linolate | 3 | tubeculate* | 2 | reticular | 3 | S+shiny | 3 | verrucose |  |  |  |  |  |  |  |
| 14 | leaf venation apparentness | **1** | inapparent | 1 | apparent** | 0 |  |  |  |  |  |  |  |  |  |  |  |  |  |  |  |  |
| 15 | leaf venation-pinnate versus palmate | **9** | pinnate | 7 | palmate** | 2 |  |  |  |  |  |  |  |  |  |  |  |  |  |  |  |  |
| 16 | Types of Palmate vernation | **6** | trinerved** | 1 | semi triplinerved | 1 | triplinerved | 1 | >3 nerves | 3 |  |  |  |  |  |  |  |  |  |  |  |  |
| 17 | Number of stamens | **1** | more than one** | 0 | only one | 1 |  |  |  |  |  |  |  |  |  |  |  |  |  |  |  |  |
| 18 | Stinging hairs presence | **3** | present | 1 | absent** | 2 |  |  |  |  |  |  |  |  |  |  |  |  |  |  |  |  |
| 19 | Filament | **2** | inflexed** | 0 | straight | 2 |  |  |  |  |  |  |  |  |  |  |  |  |  |  |  |  |
| 20 | Hair apex angle | **1** | hooked (incongruent) | 1 | congruent with axis** | 0 |  |  |  |  |  |  |  |  |  |  |  |  |  |  |  |  |
|  |  |  |  |  |  |  |  |  |  |  |  |  |  |  |  |  |  |  |  |  |  |  |
| *NB where clades comprise several accessions with more than one state, this is treated as a maximum of one origin for each state concerned.* | | | | | | | | | | | | | | | | | | | | | | |
| ***Two Asterisks indicate unequivocal ancestral state; number of origins following is* ***reversals*** *to this state only.* | | | | | | | | | | | | | | | | | | | | | | |
| ** indicates possible ancestral state, one of two.* | | | | | | | | | | | | | | | | | | | | | | |
| "TOTAL" shows total number of changes to that character inferred. May be higher than sum of origins states, because it includes ambiguities where (e.g.) 2 changes are inferred but exact type of change is uncertain. | | | | | | | | | | | | | | | | | | | | | | |
