# Supplementary material for: Ancestral State Reconstruction Reveals Rampant Homoplasy of Diagnostic Morphological Characters in Urticaceae, Conflicting with Current Classification Schemes
Source: PLoS One. 2015 Nov 3;10(11):e0141821. doi: 10.1371/journal.pone.0141821 (PMC4631448; doi:10.1371/journal.pone.0141821)
Supplement: S3 Table — (DOCX) [file pone.0141821.s026.docx]

| **Table S2. The data matrixes of MP** | | | | | | | | | | | | | | | | | | | | |
| --- | --- | --- | --- | --- | --- | --- | --- | --- | --- | --- | --- | --- | --- | --- | --- | --- | --- | --- | --- | --- |
| species | habit | cystolith presence | cystolith form | stigma form | phyllotaxis | stipule presence | stipule form | stipule fusion | stipule position | pistillate perianth presence | pistillate perianth lobes fusion | achene symmetry | external morphology of achene | leaf vernation apparentness | leaf vernation – pinnate versus palmate | types of palmate venation | number of stamens | stinging hairs presence | filament | hair apex angle |
| Archiboehmeria_atrata_A1 | 0 | 1 | 0 | 4 | 0 | 1 | 0 | 1 | 0 | 1 | 1 | 0 | 6 | 1 | 1 | 0 | 0 | 1 | 0 | 1 |
| Astrothalamus_reticulatus_23592 | 1 | 1 | 0 | 2 | 0 | 1 | 0 | 0 | 1 | 1 | 1 | 0 | 0 | 1 | 1 | 0 | 0 | 1 | 0 | 1 |
| Debregeasia_saeneb_De17 | 1 | 1 | 0 | 2 | 0 | 1 | 0 | 1 | 0 | 1 | 1 | 0 | 0 | 1 | 1 | 0 | 0 | 1 | 0 | 1 |
| Debregeasia_sp_De25 | 1 | 1 | 0 | 2 | 0 | 1 | 0 | 1 | 0 | 1 | 1 | 0 | 0 | 1 | 1 | 0 | 0 | 1 | 0 | 1 |
| Debregeasia_orientalis_De15 | 1 | 1 | 0 | 2 | 0 | 1 | 0 | 1 | 0 | 1 | 1 | 0 | 0 | 1 | 1 | 0 | 0 | 1 | 0 | 1 |
| Debregeasia_orientalis_De13 | 1 | 1 | 0 | 2 | 0 | 1 | 0 | 1 | 0 | 1 | 1 | 0 | 0 | 1 | 1 | 0 | 0 | 1 | 0 | 1 |
| Debregeasia_squamata_De5 | 1 | 1 | 0 | 2 | 0 | 1 | 0 | 1 | 0 | 1 | 1 | 0 | 0 | 1 | 1 | 0 | 0 | 1 | 0 | 1 |
| Debregeasia_longifolia_De10 | 1 | 1 | 0 | 2 | 0 | 1 | 0 | 1 | 0 | 1 | 1 | 0 | 6 | 1 | 1 | 0 | 0 | 1 | 0 | 1 |
| Debregeasia_longifolia_De9 | 1 | 1 | 0 | 2 | 0 | 1 | 0 | 1 | 0 | 1 | 1 | 0 | 6 | 1 | 1 | 0 | 0 | 1 | 0 | 1 |
| Debregeasia_elliptica_De19 | 1 | 1 | 0 | 2 | 0 | 1 | 0 | 1 | 0 | 1 | 1 | 0 | 0 | 1 | 1 | 0 | 0 | 1 | 0 | 1 |
| Debregeasia_elliptica_De7 | 1 | 1 | 0 | 2 | 0 | 1 | 0 | 1 | 0 | 1 | 1 | 0 | 0 | 1 | 1 | 0 | 0 | 1 | 0 | 1 |
| Boehmeria_glomerulifera_B5 | 1 | 1 | 0 | 1 | 0 | 1 | 0 | 0 | 1 | 1 | 1 | 0 | 0 | 1 | 1 | 0 | 0 | 1 | 0 | 1 |
| Boehmeria_macrophylla_var_macrophylla_B28 | 0 | 1 | 0 | 1 | 1 | 1 | 0 | 0 | 1 | 1 | 1 | 0 | 0 | 1 | 1 | 0 | 0 | 1 | 0 | 1 |
| Boehmeria_macrophylla_var_macrophylla_B24 | 0 | 1 | 0 | 1 | 1 | 1 | 0 | 0 | 1 | 1 | 1 | 0 | 0 | 1 | 1 | 0 | 0 | 1 | 0 | 1 |
| Boehmeria_sp_B46 | 0 | 1 | 0 | 1 | 1 | 1 | 0 | 0 | 1 | 1 | 1 | 0 | 0 | 1 | 1 | 0 | 0 | 1 | 0 | 1 |
| Boehmeria_spicata_B9 | 0 | 1 | 0 | 1 | 1 | 1 | 0 | 0 | 1 | 1 | 1 | 0 | 0 | 1 | 1 | 0 | 0 | 1 | 0 | 1 |
| Boehmeria_japonica_B47 | 0 | 1 | 0 | 1 | 1 | 1 | 0 | 0 | 1 | 1 | 1 | 0 | 0 | 1 | 1 | 0 | 0 | 1 | 0 | 1 |
| Boehmeria_tricuspis_B39 | 0 | 1 | 0 | 1 | 1 | 1 | 0 | 0 | 1 | 1 | 1 | 0 | 0 | 1 | 1 | 0 | 0 | 1 | 0 | 1 |
| Boehmeria_longispica_B20 | 0 | 1 | 0 | 1 | 1 | 1 | 0 | 0 | 1 | 1 | 1 | 0 | 0 | 1 | 1 | 0 | 0 | 1 | 0 | 1 |
| Boehmeria_densiflora_B52 | (0 1) | 1 | 0 | 1 | 1 | 1 | 0 | 0 | 1 | 1 | 1 | 0 | 0 | 1 | 1 | 0 | 0 | 1 | 0 | 1 |
| Boehmeria_densiflora_B53 | (0 1) | 1 | 0 | 1 | 1 | 1 | 0 | 0 | 1 | 1 | 1 | 0 | 0 | 1 | 1 | 0 | 0 | 1 | 0 | 1 |
| Boehmeria_clidemioides_var_clidemioides_B2 | 0 | 1 | 0 | 1 | 1 | 1 | 0 | 0 | 1 | 1 | 1 | 0 | 0 | 1 | 1 | 0 | 0 | 1 | 0 | 1 |
| Boehmeria_macrophylla_var_rotundifolia_B21 | 0 | 1 | 0 | 1 | 1 | 1 | 0 | 0 | 1 | 1 | 1 | 0 | 0 | 1 | 1 | 0 | 0 | 1 | 0 | 1 |
| Boehmeria_clidemioides_var_clidemioides_B15 | 0 | 1 | 0 | 1 | 1 | 1 | 0 | 0 | 1 | 1 | 1 | 0 | 0 | 1 | 1 | 0 | 0 | 1 | 0 | 1 |
| Boehmeria_zollingeriana_var_blinii_B1 | 0 | 1 | 0 | 1 | (0 1) | 1 | 0 | 0 | 1 | 1 | 1 | 0 | 0 | 1 | 1 | 0 | 0 | 1 | 0 | 1 |
| Boehmeria_penduliflora_B33 | 0 | 1 | 0 | 1 | 0 | 1 | 0 | 0 | 1 | 1 | 1 | 0 | 0 | 1 | 1 | 0 | 0 | 1 | 0 | 1 |
| Boehmeria_umbrosa_B40 | 0 | 1 | 0 | 1 | 1 | 1 | 0 | 0 | 1 | 1 | 1 | 0 | 0 | 1 | 1 | 0 | 0 | 1 | 0 | 1 |
| Boehmeria_umbrosa_B12 | 0 | 1 | 0 | 1 | 1 | 1 | 0 | 0 | 1 | 1 | 1 | 0 | 0 | 1 | 1 | 0 | 0 | 1 | 0 | 1 |
| Boehmeria_clidemioides_var_diffusa_B16 | 0 | 1 | 0 | 1 | 0 | 1 | 0 | 0 | 1 | 1 | 1 | 0 | 0 | 1 | 1 | 0 | 0 | 1 | 0 | 1 |
| Boehmeria_macrophylla_var_scabrella_B26 | 0 | 1 | 0 | 1 | 1 | 1 | 0 | 0 | 1 | 1 | 1 | 0 | 0 | 1 | 1 | 0 | 0 | 1 | 0 | 1 |
| Chamabainia_cuspidata_C1 | 0 | 1 | 0 | 0 | 1 | 1 | 2 | 0 | 1 | 1 | 1 | 0 | 5 | 1 | 1 | 0 | 0 | 1 | 0 | 1 |
| Chamabainia_cuspidata_C2 | 0 | 1 | 0 | 0 | 1 | 1 | 2 | 0 | 1 | 1 | 1 | 0 | 5 | 1 | 1 | 0 | 0 | 1 | 0 | 1 |
| Neraudia_melastomifolia_Ne1 | 1 | 1 | 0 | 1 | 0 | 1 | 2 | 1 | 0 | 1 | 1 | 0 | 5 | 1 | 1 | 0 | 0 | 1 | 0 | 1 |
| Neraudia_kauaiensis_Ne2 | 1 | 1 | 0 | 1 | 0 | 1 | 2 | 1 | 0 | 1 | 1 | 0 | 5 | 1 | 1 | 0 | 0 | 1 | 0 | 1 |
| Pouzolzia_argenteonitida_Po5 | 1 | 1 | 0 | 1 | 0 | 1 | 2 | 0 | 1 | 1 | 1 | 0 | 5 | 1 | 1 | 0 | 0 | 1 | 0 | 1 |
| Meniscogyne_sp_154A | ? | ? | ? | ? | ? | ? | ? | ? | ? | ? | ? | ? | ? | ? | ? | ? | ? | ? | ? | ? |
| Boehmeria_rugulosa_B45 | 1 | 1 | 0 | 1 | 0 | 1 | 0 | 0 | 1 | 1 | 1 | 0 | 0 | 1 | 1 | 0 | 0 | 1 | 0 | 1 |
| Pouzolzia_sanguinea_var_sanguinea_Po6 | 1 | 1 | 0 | 1 | 0 | 1 | 2 | 0 | 1 | 1 | 1 | 0 | 5 | 1 | 1 | 0 | 0 | 1 | 0 | 1 |
| Pouzolzia_sanguinea_var_elegans_Po2 | 1 | 1 | 0 | 1 | 0 | 1 | 2 | 0 | 1 | 1 | 1 | 0 | 5 | 1 | 1 | 0 | 0 | 1 | 0 | 1 |
| Nothocnide_mollissima_23585 | 1 | 1 | 0 | 1 | 0 | 1 | 0 | 1 | 0 | 1 | 1 | 0 | 0 | 1 | 1 | 0 | 0 | 1 | 0 | 1 |
| Pipturus_kauaiensis_Pip5 | 1 | 1 | 0 | 1 | 0 | 1 | 0 | 1 | 0 | 1 | 1 | 0 | 5 | 1 | 1 | 0 | 0 | 1 | 0 | 1 |
| Pipturus_ruber_Pip6 | 1 | 1 | 0 | 1 | 0 | 1 | 0 | 1 | 0 | 1 | 1 | 0 | 5 | 1 | 1 | 0 | 0 | 1 | 0 | 1 |
| Pipturus_arborescens_Pip7 | 1 | 1 | 0 | 1 | 0 | 1 | 0 | 1 | 0 | 1 | 1 | 0 | 5 | 1 | 1 | 0 | 0 | 1 | 0 | 1 |
| Pipturus_arborescens_Pip1 | 1 | 1 | 0 | 1 | 0 | 1 | 0 | 1 | 0 | 1 | 1 | 0 | 5 | 1 | 1 | 0 | 0 | 1 | 0 | 1 |
| Gonostegia_hirta_Go3 | 0 | 1 | 0 | 1 | 1 | 1 | 1 | 1 | 0 | 1 | 1 | 0 | 5 | 1 | 1 | 0 | 0 | 1 | 0 | 1 |
| Gonostegia_pavifolia_Go1 | 0 | 1 | 0 | 1 | 1 | 1 | 1 | 1 | 0 | 1 | 1 | 0 | 5 | 1 | 1 | 0 | 0 | 1 | 0 | 1 |
| Gonostegia_pavifolia_Go4 | 0 | 1 | 0 | 1 | 1 | 1 | 1 | 1 | 0 | 1 | 1 | 0 | 5 | 1 | 1 | 0 | 0 | 1 | 0 | 1 |
| Pouzolzia_zeylanica_var_zeylanica_Po7 | 0 | 1 | 0 | 1 | 1 | 1 | 2 | 0 | 1 | 1 | 1 | 0 | 5 | 1 | 1 | 0 | 0 | 1 | 0 | 1 |
| Pouzolzia_sp_Po9 | 0 | 1 | 0 | 1 | 1 | 1 | 2 | 0 | 1 | 1 | 1 | 0 | 5 | 1 | 1 | 0 | 0 | 1 | 0 | 1 |
| Pouzolzia_zeylanica_var_zeylanica_Po4 | 0 | 1 | 0 | 1 | 1 | 1 | 2 | 0 | 1 | 1 | 1 | 0 | 5 | 1 | 1 | 0 | 0 | 1 | 0 | 1 |
| Pouzolzia_guineensis_282A | 0 | 1 | 0 | 1 | 0 | 1 | 2 | 0 | 1 | 1 | 1 | 0 | 5 | 1 | 1 | 0 | 0 | 1 | 0 | 1 |
| Pouzolzia_mixta_288A | 1 | 1 | 0 | 1 | 0 | 1 | 2 | 0 | 1 | 1 | 1 | 0 | 5 | 1 | 1 | 0 | 0 | 1 | 0 | 1 |
| Hemistylus_macrostachya_23597 | 1 | 1 | 0 | 1 | 0 | 1 | 1 | 0 | 1 | 1 | 1 | 0 | 5 | 1 | 1 | 0 | 0 | 1 | 0 | 1 |
| Rousselia_humulis_23596 | 0 | 1 | 0 | 1 | 0 | 1 | 0 | 0 | 1 | 1 | 1 | 0 | 5 | 1 | 1 | 0 | 0 | 1 | 0 | 1 |
| Neodistemon_indicum_279A | 0 | 1 | 0 | 1 | 0 | 1 | 2 | 0 | 1 | 1 | 1 | 0 | 5 | 1 | 1 | 0 | 0 | 1 | 0 | 1 |
| Oreocnide_frutescens_subsp_frutescens_O8 | 1 | 1 | 0 | 5 | 0 | 1 | 0 | 0 | 1 | 1 | 1 | 0 | 4 | 1 | 1 | 0 | 0 | 1 | 0 | 1 |
| Oreocnide_frutescens_subsp_frutescens_O2 | 1 | 1 | 0 | 5 | 0 | 1 | 0 | 0 | 1 | 1 | 1 | 0 | 4 | 1 | 1 | 0 | 0 | 1 | 0 | 1 |
| Oreocnide_frutescens_subsp_occidentalis_O12 | 1 | 1 | 0 | 5 | 0 | 1 | 0 | 0 | 1 | 1 | 1 | 0 | 4 | 1 | 1 | 0 | 0 | 1 | 0 | 1 |
| Soleirolia_soleirolii_312A | 0 | 1 | (0 2) | 0 | 0 | 0 | - | - | - | 1 | 1 | 1 | 5 | 1 | 1 | 0 | 0 | 1 | 0 | 1 |
| Gesnouinia_arborea_177A | 1 | 1 | 0 | 1 | 0 | 0 | - | - | - | 1 | 1 | 0 | 5 | 1 | 1 | 0 | 0 | 1 | 0 | 1 |
| Parietaria_judaica_11077 | 0 | 1 | 0 | 0 | 0 | 0 | - | - | - | 1 | 1 | 0 | 5 | 1 | 1 | 0 | 0 | 1 | 0 | 1 |
| Parietaria_micrantha_Pa1 | 0 | 1 | 0 | 0 | 0 | 0 | - | - | - | 1 | 1 | 0 | 5 | 1 | 1 | 0 | 0 | 1 | 0 | 1 |
| Forsskaolea_angustifolia_16132 | 0 | 1 | 0 | 1 | 0 | 1 | 1 | 0 | 1 | 0 | - | 0 | 0 | 1 | 1 | 0 | 1 | 1 | 0 | 0 |
| Forsskaolea_angustifolia_6515 | 0 | 1 | 0 | 1 | 0 | 1 | 1 | 0 | 1 | 0 | - | 0 | 0 | 1 | 1 | 0 | 1 | 1 | 0 | 0 |
| Droguetia_ambigua_28892 | 0 | 1 | 0 | 1 | 1 | 1 | 0 | 0 | 1 | 1 | 1 | 0 | 0 | 1 | 1 | 0 | 1 | 1 | 0 | 1 |
| Droguetia_iner_subsp_urticoides_Dr1 | 0 | 1 | 0 | 1 | 1 | 1 | 0 | 0 | 1 | 1 | 1 | 0 | 0 | 1 | 1 | 0 | 1 | 1 | 0 | 1 |
| Droguetia_iner_subsp_urticoides_Dr4 | 0 | 1 | 0 | 1 | 1 | 1 | 0 | 0 | 1 | 1 | 1 | 0 | 0 | 1 | 1 | 0 | 1 | 1 | 0 | 1 |
| Australina_flaccida_23601 | 0 | 1 | 0 | 1 | 1 | 1 | 0 | 0 | 1 | 1 | 1 | 0 | 3 | 1 | 1 | 0 | 1 | 1 | 0 | 1 |
| Didymodoxa_caffra_23599 | 0 | 1 | 0 | 1 | 0 | 1 | 0 | 0 | 1 | 1 | 1 | 0 | 5 | 1 | 1 | 0 | 1 | 1 | 0 | 1 |
| Phenax_mexicanus_378A | 1 | 1 | 0 | 1 | 0 | 1 | 0 | 0 | 1 | 0 | - | 0 | 3 | 1 | 1 | 0 | 0 | 1 | 0 | 1 |
| Coussapoa_parvifolia_386A | 1 | 0 | - | 0 | 0 | 1 | 0 | 1 | 0 | 1 | 1 | 0 | 4 | 1 | 0 | - | 0 | 1 | 1 | 1 |
| Myrianthus_preussii_23604 | 1 | 0 | - | 4 | 0 | 1 | 0 | 1 | 0 | 1 | 1 | 0 | ? | 1 | 1 | 3 | 0 | 1 | 1 | 1 |
| Cecropia_ficifolia_23606 | 1 | 0 | - | 5 | 0 | 1 | 0 | 1 | 0 | 1 | 1 | 0 | 3 | 1 | 1 | 3 | 0 | 1 | 1 | 1 |
| Cecropia_obtusifolia_162A | 1 | 0 | - | 5 | 0 | 1 | 0 | 1 | 0 | 1 | 1 | 0 | 0 | 1 | 1 | 3 | 0 | 1 | 1 | 1 |
| Leucosyke_quadrinervia_Leu3 | 1 | 0 | - | 0 | 0 | 1 | 0 | 1 | 0 | 1 | 1 | 1 | 3 | 1 | 1 | 3 | 0 | 1 | 0 | 1 |
| Leucosyke_quadrinervia_Leu4 | 1 | 0 | - | 0 | 0 | 1 | 0 | 1 | 0 | 1 | 1 | 1 | 3 | 1 | 1 | 3 | 0 | 1 | 0 | 1 |
| Maoutia_setosa_M2 | 1 | 0 | - | (0 5) | 0 | 1 | 0 | 1 | 0 | 1 | 1 | 0 | (0 3) | 1 | 1 | 0 | 0 | 1 | 0 | 1 |
| Fatoua_villosa_F2 | 0 | 0 | - | 1 | 0 | 1 | 3 | 0 | 1 | 1 | 0 | 1 | (3 6) | 1 | 1 | 0 | 0 | 1 | 0 | 1 |
| Fatoua_villosa_F1 | 0 | 0 | - | 1 | 0 | 1 | 3 | 0 | 1 | 1 | 0 | 1 | (3 6) | 1 | 1 | 0 | 0 | 1 | 0 | 1 |
| Humulus_scandense_H1 | 0 | 0 | - | 1 | 1 | 1 | 3 | 0 | 1 | 1 | 1 | 0 | 0 | 1 | 1 | 3 | 0 | 1 | 1 | 1 |
| Celtis_kunmingensis_Ulm2 | 1 | 0 | - | 1 | 0 | 1 | ? | ? | ? | 1 | ? | ? | ? | 1 | 1 | 0 | 0 | 1 | 1 | 1 |
| Pellionia_tsoongii_Pe5 | 0 | 1 | 1 | 0 | 0 | 1 | 1 | 0 | 1 | 1 | 0 | 0 | 3 | 1 | 1 | 1 | 0 | 1 | 0 | 1 |
| Pellionia_macrophylla_Pe1 | 0 | 1 | 1 | 0 | 0 | 1 | 0 | 0 | 1 | 1 | 0 | 0 | 2 | 1 | 0 | - | 0 | 1 | 0 | 1 |
| Pellionia_radicans_Pe3 | 0 | 1 | 1 | 0 | 0 | 1 | 3 | 0 | 1 | 1 | 0 | 0 | 3 | 1 | 1 | 1 | 0 | 1 | 0 | 1 |
| Pellionia_paucidentata_var_paucidentata_Pe2 | 0 | 1 | 1 | 0 | 0 | 1 | 3 | 0 | 1 | 1 | 0 | 0 | 3 | 1 | 1 | 1 | 0 | 1 | 0 | 1 |
| Elatostema_parvum_var_parvum_E7 | 0 | 1 | 1 | 0 | 0 | 1 | 3 | 0 | 1 | 1 | 0 | 0 | 1 | 1 | 1 | (0 1) | 0 | 1 | 0 | 1 |
| Elatostema_densistriolatum_E9 | 0 | 1 | 1 | 0 | 0 | 1 | 5 | 0 | 1 | 1 | 0 | 0 | 2 | 1 | 1 | 0 | 0 | 1 | 0 | 1 |
| Elatostema_petelotii_E8 | 0 | 1 | 1 | 0 | 0 | 1 | 3 | 0 | 1 | 1 | 0 | 0 | 0 | 1 | 1 | 0 | 0 | 1 | 0 | 1 |
| Elatostema_cuspidatum_var_cuspidatum_E4 | 0 | 1 | 1 | 0 | 0 | 1 | 3 | 0 | 1 | 1 | 0 | 0 | 1 | 1 | 1 | 1 | 0 | 1 | 0 | 1 |
| Elatostema_crytandrifolium_var_crytandrifolium_E3 | 0 | 1 | 1 | 0 | 0 | 1 | 2 | 0 | 1 | 1 | 0 | 0 | 1 | 1 | 1 | (0 1) | 0 | 1 | 0 | 1 |
| Elatostema_tenuicaudatum_var_tenuicaudatum_E12 | 0 | 1 | 1 | 0 | 0 | 1 | 3 | 0 | 1 | 1 | 0 | 0 | 1 | 1 | 1 | (0 2) | 0 | 1 | 0 | 1 |
| Elatostema_longibracteatum_E6 | 0 | 1 | 1 | 0 | 0 | 1 | 3 | 0 | 1 | 1 | 0 | 0 | 1 | 1 | 1 | 1 | 0 | 1 | 0 | 1 |
| Elatostema_stewardii_E10 | 0 | 1 | 1 | 0 | 0 | 1 | 2 | 0 | 1 | 1 | 0 | 0 | 1 | 1 | 0 | - | 0 | 1 | 0 | 1 |
| Elatostema_atropurpureum_E2 | 0 | 1 | 1 | 0 | 0 | 1 | 2 | 0 | 1 | 1 | 0 | 0 | 1 | 1 | 0 | - | 0 | 1 | 0 | 1 |
| Elatostema_albopilosum_E1 | 0 | 1 | 1 | 0 | 0 | 1 | 3 | 0 | 1 | 1 | 0 | 0 | 1 | 1 | 0 | - | 0 | 1 | 0 | 1 |
| Elatostema_subtrichotomum_var_subtrichotomum_E11 | 0 | 1 | 1 | 0 | 0 | 1 | 3 | 0 | 1 | 1 | 0 | 0 | 3 | 1 | ? | ? | 0 | 1 | 0 | 1 |
| Elatostema_sp_E13 | 0 | 1 | 1 | 0 | 0 | 1 | 3 | 0 | 1 | 1 | 0 | 0 | 1 | 1 | 1 | 1 | 0 | 1 | 0 | 1 |
| Pellionia_repens_Pe4 | 0 | 1 | 1 | 0 | 0 | 1 | 1 | 0 | 1 | 1 | 0 | 0 | 3 | 1 | 1 | 1 | 0 | 1 | 0 | 1 |
| Procris_wightiana_Pr2 | 0 | 1 | 1 | 0 | 0 | 1 | 5 | 1 | 0 | 1 | 0 | 0 | 2 | 1 | 0 | - | 0 | 1 | 0 | 1 |
| Procris_wightiana_Pr1 | 0 | 1 | 1 | 0 | 0 | 1 | 5 | 1 | 0 | 1 | 0 | 0 | 2 | 1 | 0 | - | 0 | 1 | 0 | 1 |
| Gyrotaenia_crassifolia_475A | 1 | 1 | 1 | 2 | 0 | 1 | 0 | 0 | 1 | 1 | 0 | 0 | 4 | 1 | 1 | 0 | 0 | 1 | 0 | 1 |
| Gyrotaenia_microcarpa_473A | 1 | 1 | 1 | 2 | 0 | 1 | 0 | 0 | 1 | 1 | 0 | 0 | 4 | 1 | 1 | 0 | 0 | 1 | 0 | 1 |
| Myriocarpa_obovata_370A | 1 | 1 | 2 | 8 | 0 | 1 | 0 | 1 | 0 | 1 | 1 | 0 | 0 | 1 | 1 | 0 | 0 | 1 | 0 | 1 |
| Gyrotaenia_spicata_23567 | 1 | 1 | 1 | 2 | 0 | 1 | 0 | 0 | 1 | 1 | 0 | 0 | 4 | 1 | 1 | 0 | 0 | 1 | 0 | 1 |
| Myriocarpa_cordata_C2A | 1 | 1 | 2 | 8 | 0 | 1 | 0 | 1 | 0 | 1 | 1 | 0 | 0 | 1 | 0 | - | 0 | 1 | 0 | 1 |
| Lecanthus_petelotii_var_corniculata_Le4 | 0 | 1 | 1 | 0 | 1 | 1 | 4 | 1 | 0 | 1 | 0 | 0 | 3 | 1 | 1 | 0 | 0 | 1 | 0 | 1 |
| Lecanthus_peduncularis_Le1 | 0 | 1 | 1 | 0 | 1 | 1 | 4 | 1 | 0 | 1 | 0 | 0 | 3 | 1 | 1 | 0 | 0 | 1 | 0 | 1 |
| Lecanthus_peduncularis_Le3 | 0 | 1 | 1 | 0 | 1 | 1 | 4 | 1 | 0 | 1 | 0 | 0 | 3 | 1 | 1 | 0 | 0 | 1 | 0 | 1 |
| Lecanthus_petelotii_var_corniculata_Le2 | 0 | 1 | 1 | 0 | 1 | 1 | 4 | 1 | 0 | 1 | 0 | 0 | 3 | 1 | 1 | 0 | 0 | 1 | 0 | 1 |
| Pilea_cavaleriei_subsp_cavaleriei_P3 | 0 | 1 | 0 | 0 | 1 | 1 | 2 | 1 | 0 | 1 | 0 | 1 | 0 | 1 | 1 | 0 | 0 | 1 | 0 | 1 |
| Pilea_pumila_var_pumila_P10 | 0 | 1 | 1 | 0 | 1 | 1 | 2 | 1 | 0 | 1 | 0 | 1 | 6 | 1 | 1 | 0 | 0 | 1 | 0 | 1 |
| Pilea_melastomoides_P20 | 0 | 1 | 1 | 0 | 1 | 1 | 2 | 1 | 0 | 1 | 0 | 0 | 0 | 1 | 1 | 0 | 0 | 1 | 0 | 1 |
| Pilea_angulata_subsp_petiplaris_P1 | 0 | 1 | 0 | 0 | 1 | 1 | 4 | 1 | 0 | 1 | 0 | 1 | 6 | 1 | 1 | 0 | 0 | 1 | 0 | 1 |
| Pilea_verrucossa_subsp_verrucossa_P29 | 0 | 1 | 1 | 0 | 1 | 1 | 2 | 1 | 0 | 1 | 0 | 1 | 6 | 1 | 1 | 0 | 0 | 1 | 0 | 1 |
| Pilea_martinii_P6 | 0 | 1 | 1 | 0 | 1 | 1 | 0 | 1 | 0 | 1 | 0 | 1 | 0 | 1 | 1 | 0 | 0 | 1 | 0 | 1 |
| Pilea_sp_P12 | 0 | 1 | 1 | 0 | 1 | 1 | 0 | 1 | 0 | 1 | 0 | 1 | 0 | 1 | 1 | 0 | 0 | 1 | 0 | 1 |
| Pilea_insolens_P11 | 0 | 1 | 2 | 0 | 1 | 1 | 5 | 1 | 0 | 1 | 0 | 1 | 0 | 1 | 1 | 0 | 0 | 1 | 0 | 1 |
| Pilea_oxyodon_P9 | 0 | 1 | 2 | 0 | 1 | 1 | 5 | 1 | 0 | 1 | 0 | 1 | 0 | 1 | 1 | 0 | 0 | 1 | 0 | 1 |
| Sarcopilea_domingensis_302A | 0 | 1 | 1 | 0 | 0 | 1 | 1 | 1 | 0 | 1 | 0 | 0 | 0 | 0 | - | - | 0 | 1 | 0 | 1 |
| Pilea_microphylla_P21 | 0 | 1 | 1 | 0 | 1 | 1 | 2 | 1 | 0 | 1 | 0 | 1 | 0 | 1 | 1 | 0 | 0 | 1 | 0 | 1 |
| Pilea_microphylla_P22 | 0 | 1 | 1 | 0 | 1 | 1 | 2 | 1 | 0 | 1 | 0 | 1 | 0 | 1 | 1 | 0 | 0 | 1 | 0 | 1 |
| Pilea_plantaniflora_P24 | 0 | 1 | 1 | 0 | 1 | 1 | 2 | 1 | 0 | 1 | 0 | 1 | 6 | 1 | 1 | 0 | 0 | 1 | 0 | 1 |
| Pilea_longipedunculata_P5 | 0 | 1 | 2 | 0 | 1 | 1 | 2 | 1 | 0 | 1 | 0 | 1 | 6 | 1 | 1 | 0 | 0 | 1 | 0 | 1 |
| Pilea_sinofasciata_P26 | 0 | 1 | 1 | 0 | 1 | 1 | 2 | 1 | 0 | 1 | 0 | 1 | 6 | 1 | 1 | 0 | 0 | 1 | 0 | 1 |
| Girardinia_diversifolia_subsp_triloba_G6 | 0 | 1 | 0 | 3 | 0 | 1 | 4 | 1 | 0 | 1 | 0 | 1 | 0 | 1 | 1 | 0 | 0 | 0 | 0 | 1 |
| Girardinia_diversifolia_subsp_triloba_G19 | 0 | 1 | 0 | 3 | 0 | 1 | 4 | 1 | 0 | 1 | 0 | 1 | 0 | 1 | 1 | 0 | 0 | 0 | 0 | 1 |
| Girardinia_diversifolia_subsp_diversifolia_G9 | 0 | 1 | 0 | 3 | 0 | 1 | 4 | 1 | 0 | 1 | 0 | 1 | (0 6) | 1 | 1 | 0 | 0 | 0 | 0 | 1 |
| Girardinia_diversifolia_subsp_diversifolia_G31 | 0 | 1 | 0 | 3 | 0 | 1 | 4 | 1 | 0 | 1 | 0 | 1 | (0 6) | 1 | 1 | 0 | 0 | 0 | 0 | 1 |
| Girardinia_diversifolia_subsp_suborbiculata_G17 | 0 | 1 | 0 | 3 | 0 | 1 | 4 | 1 | 0 | 1 | 0 | 1 | 0 | 1 | 1 | 0 | 0 | 0 | 0 | 1 |
| Girardinia_diversifolia_subsp_suborbiculata_G16 | 0 | 1 | 0 | 3 | 0 | 1 | 4 | 1 | 0 | 1 | 0 | 1 | 0 | 1 | 1 | 0 | 0 | 0 | 0 | 1 |
| Nanocnide_lobata_N6 | 0 | 1 | 2 | 0 | 0 | 1 | 1 | 0 | 1 | 1 | 0 | 0 | 6 | 1 | 1 | 0 | 0 | 0 | 0 | 1 |
| Nanocnide_lobata_N5 | 0 | 1 | 2 | 0 | 0 | 1 | 1 | 0 | 1 | 1 | 0 | 0 | 6 | 1 | 1 | 0 | 0 | 0 | 0 | 1 |
| Nanocnide_japonica_N1 | 0 | 1 | 2 | 0 | 0 | 1 | 1 | 0 | 1 | 1 | 0 | 0 | 0 | 1 | 1 | 0 | 0 | 0 | 0 | 1 |
| Nanocnide_japonica_N4 | 0 | 1 | 2 | 0 | 0 | 1 | 1 | 0 | 1 | 1 | 0 | 0 | 0 | 1 | 1 | 0 | 0 | 0 | 0 | 1 |
| Urtica_sp_U19 | 0 | 1 | 0 | 0 | 1 | 1 | 3 | 0 | 1 | 1 | 0 | 0 | 6 | 1 | 1 | 0 | 0 | 0 | 0 | 1 |
| Urtica_triangularis_subsp_pinnatifida_U10 | 0 | 1 | 0 | 0 | 1 | 1 | 3 | 0 | 1 | 1 | 0 | 0 | 6 | 1 | 1 | 0 | 0 | 0 | 0 | 1 |
| Urtica_hyperborea_U5 | 0 | 1 | 0 | 0 | 1 | 1 | 4 | 0 | 1 | 1 | 0 | 0 | 0 | 1 | 1 | 0 | 0 | 0 | 0 | 1 |
| Urtica_hyperborea_U14 | 0 | 1 | 0 | 0 | 1 | 1 | 4 | 0 | 1 | 1 | 0 | 0 | 0 | 1 | 1 | 0 | 0 | 0 | 0 | 1 |
| Urtica_atrichocaulis_U3 | 0 | 1 | 0 | 0 | 1 | 1 | 3 | 0 | 1 | 1 | 0 | 0 | 0 | 1 | 1 | 0 | 0 | 0 | 0 | 1 |
| Urtica_dioica_U21 | 0 | 1 | 0 | 0 | 1 | 1 | 3 | 0 | 1 | 1 | 0 | 0 | 0 | 1 | 1 | 0 | 0 | 0 | 0 | 1 |
| Urtica_angustifolia_U1 | 0 | 1 | 0 | 0 | 1 | 1 | 3 | 0 | 1 | 1 | 0 | 0 | 6 | 1 | 1 | 0 | 0 | 0 | 0 | 1 |
| Urtica_sp_U18 | 0 | 1 | 0 | 0 | 1 | 1 | 3 | 0 | 1 | 1 | 0 | 0 | 0 | 1 | 1 | 0 | 0 | 0 | 0 | 1 |
| Hesperocnide_tenella_331A | 0 | 1 | 0 | 0 | 1 | 1 | 3 | 0 | 1 | 1 | 1 | 0 | 0 | 1 | 1 | 0 | 0 | 0 | 0 | 1 |
| Urtica_ardens_U2 | 0 | 1 | 0 | 0 | 1 | 1 | 4 | 1 | 1 | 1 | 0 | 0 | 6 | 1 | 1 | 0 | 0 | 0 | 0 | 1 |
| Urtica_fissa_U4 | 0 | 1 | (0 2) | 0 | 1 | 1 | 4 | 1 | 1 | 1 | 0 | 0 | 6 | 1 | 1 | 3 | 0 | 0 | 0 | 1 |
| Urtica_zayuensis_U17 | 0 | 1 | 0 | 0 | 1 | 1 | 1 | 1 | 1 | 1 | 0 | 0 | 0 | 1 | 1 | 3 | 0 | 0 | 0 | 1 |
| Urtica_zayuensis_U11 | 0 | 1 | 0 | 0 | 1 | 1 | 1 | 1 | 1 | 1 | 0 | 0 | 0 | 1 | 1 | 3 | 0 | 0 | 0 | 1 |
| Urtica_mairei_U7 | 0 | 1 | (0 2) | 0 | 1 | 1 | 4 | 1 | 1 | 1 | 0 | 0 | 6 | 1 | 1 | 3 | 0 | 0 | 0 | 1 |
| Discocnide_mexicana_167A | 1 | 1 | 0 | (3 4) | 0 | 1 | 0 | 1 | 0 | 1 | 0 | 1 | 0 | 1 | 1 | 0 | 0 | 0 | 0 | 1 |
| Dendrocnide_sinuata_D1 | 1 | 1 | 0 | 4 | 0 | 1 | 5 | 1 | 0 | 1 | 0 | 1 | 6 | 1 | 0 | - | 0 | 0 | 0 | 1 |
| Dendrocnide_meyeniana_D2 | 1 | 1 | 0 | 4 | 0 | 1 | 5 | 1 | 0 | 1 | 0 | 1 | 3 | 1 | 0 | - | 0 | 0 | 0 | 1 |
| Dendrocnide_urentissima_D5 | 1 | 1 | 0 | 4 | 0 | 1 | 5 | 1 | 0 | 1 | 0 | 1 | 6 | 1 | 0 | - | 0 | 0 | 0 | 1 |
| Dendrocnide_sp_W1 | 1 | 1 | 0 | 4 | 0 | 1 | 5 | 1 | 0 | 1 | 0 | 1 | 6 | 1 | 0 | - | 0 | 0 | 0 | 1 |
| Laportea_bulbifera_L5 | 0 | 1 | 0 | 3 | 0 | 1 | 0 | 1 | 0 | 1 | 0 | 1 | 6 | 1 | 1 | 0 | 0 | 0 | 0 | 1 |
| Laportea_bulbifera_L3 | 0 | 1 | 0 | 3 | 0 | 1 | 0 | 1 | 0 | 1 | 0 | 1 | 6 | 1 | 1 | 0 | 0 | 0 | 0 | 1 |
| Touchardia_latifolia_T1 | 1 | 1 | 0 | 9 | 0 | 1 | 0 | 1 | 0 | 1 | 0 | 0 | 5 | 1 | 0 | - | 0 | 1 | 0 | 1 |
| Urera_glabra_Ur1 | 1 | 1 | (0 2) | 0 | 0 | 1 | 0 | 1 | 0 | 1 | 0 | 1 | 3 | 1 | 0 | - | 0 | 0 | 0 | 1 |
| Obetia_tenax_28719 | 1 | 1 | 0 | (2 7) | 0 | 1 | 3 | 0 | 1 | 1 | 0 | 1 | 6 | 1 | 1 | 3 | 0 | 0 | 0 | 1 |
| Urera_sp_L2 | 1 | 1 | (0 2) | 4 | 0 | 1 | 0 | 1 | 0 | 1 | 0 | 1 | 6 | 1 | 1 | 0 | 0 | 0 | 0 | 1 |
| Urera_trinervis_374A | 1 | 1 | 2 | (2 5) | 0 | 1 | 0 | 1 | 0 | 1 | 1 | 1 | 0 | 1 | 1 | 0 | 0 | 0 | 0 | 1 |
| Urera_hypselodendron_377A | 1 | 1 | 2 | 2 | 0 | 1 | 0 | 1 | 0 | 1 | 0 | 1 | 3 | 1 | 1 | 0 | 0 | 0 | 0 | 1 |
| Poikilospermum_suaveolens_Pi2 | 1 | 1 | (0 2) | 4 | 0 | 1 | 0 | 1 | 0 | 1 | 1 | 0 | 6 | 1 | 0 | - | 0 | 1 | 1 | 1 |
| Poikilospermum_suaveolens_Pi3 | 1 | 1 | (0 2) | 4 | 0 | 1 | 0 | 1 | 0 | 1 | 1 | 0 | 6 | 1 | 0 | - | 0 | 1 | 1 | 1 |
| Poikilospermum_lanceolatum_Pi1 | 1 | 1 | (0 2) | 4 | 0 | 1 | 0 | 1 | 0 | 1 | 1 | 0 | 6 | 1 | 0 | - | 0 | 1 | 1 | 1 |
| Urera_baccifera_C4A | 1 | 1 | (0 2) | 0 | 0 | 1 | 0 | 1 | 0 | 1 | 0 | 1 | 3 | 1 | 0 | - | 0 | 0 | 0 | 1 |
| Urera_alceifolia_C11A | 1 | 1 | 1 | (2 5) | 0 | 1 | 0 | 1 | 0 | 1 | 1 | 1 | 0 | 1 | 1 | 0 | 0 | 0 | 0 | 1 |
| Urera_lianoides_313A | 1 | 1 | (0 2) | 4 | 0 | 1 | 0 | 1 | 0 | 1 | 0 | 1 | 6 | 1 | 1 | 0 | 0 | 0 | 0 | 1 |
| Urera_caracasana_23561 | 1 | 1 | (0 2) | (0 2) | 0 | 1 | 0 | 1 | 0 | 1 | 0 | 0 | 3 | 1 | 0 | - | 0 | 0 | 0 | 1 |
| Boehmeria_nivea_var_nipononivea_B32 | 1 | 1 | 0 | 1 | 0 | 1 | 0 | 0 | 1 | 1 | 1 | 0 | 0 | 1 | 1 | 0 | 0 | 1 | 0 | 1 |
| Boehmeria_nivea_var_nivea_B6 | 1 | 1 | 0 | 1 | 0 | 1 | 0 | 0 | 1 | 1 | 1 | 0 | 0 | 1 | 1 | 0 | 0 | 1 | 0 | 1 |
| Boehmeria_tomentosa_B38 | 0 | 1 | 0 | 1 | 1 | 1 | 0 | 0 | 1 | 1 | 1 | 0 | 0 | 1 | 1 | 0 | 0 | 1 | 0 | 1 |
| Sarcochlamys_pulcherrima_S1 | 1 | 1 | 0 | 6 | 0 | 1 | 0 | 0 | 1 | 1 | 0 | 1 | 0 | 1 | 1 | 0 | 0 | 1 | 0 | 1 |
| Archiboehmeria_atrata_A2 | 0 | 1 | 0 | 4 | 0 | 1 | 0 | 1 | 0 | 1 | 1 | 0 | 6 | 1 | 1 | 0 | 0 | 1 | 0 | 1 |
|  |  |  |  |  |  |  |  |  |  |  |  |  |  |  |  |  |  |  |  |  |
