# Supplementary material for: Ancestral State Reconstruction Reveals Rampant Homoplasy of Diagnostic Morphological Characters in Urticaceae, Conflicting with Current Classification Schemes
Source: PLoS One. 2015 Nov 3;10(11):e0141821. doi: 10.1371/journal.pone.0141821 (PMC4631448; doi:10.1371/journal.pone.0141821)
Supplement: S4 Table — (DOCX) [file pone.0141821.s027.docx]

| **Table S3. The data matrixes of ML** | | | | | | | | | | | | | | | | | | | | | |
| --- | --- | --- | --- | --- | --- | --- | --- | --- | --- | --- | --- | --- | --- | --- | --- | --- | --- | --- | --- | --- | --- |
| Number | species | habit | cystolith presence | cystolith form | stigma form | phyllotaxis | stipule presence | stipule form | stipule fusion | stipule position | pistillate perianth presence | pistillate perianth lobes fusion | achene symmetry | external morphology of achene | leaf vernation apparentness | leaf vernation – pinnate versus palmate | types of palmate venation | number of stamens | stinging hairs presence | filament | hair apex angle |
| 1 | Archiboehmeria_atrata_A1 | 0 | 1 | 0 | 4 | 0 | 1 | 0 | 1 | 0 | 1 | 1 | 0 | 6 | 1 | 1 | 0 | 0 | 1 | 0 | 1 |
| 2 | Astrothalamus_reticulatus_23592 | 1 | 1 | 0 | 2 | 0 | 1 | 0 | 0 | 1 | 1 | 1 | 0 | 0 | 1 | 1 | 0 | 0 | 1 | 0 | 1 |
| 3 | Debregeasia_saeneb_De17 | 1 | 1 | 0 | 2 | 0 | 1 | 0 | 1 | 0 | 1 | 1 | 0 | 0 | 1 | 1 | 0 | 0 | 1 | 0 | 1 |
| 4 | Debregeasia_sp_De25 | 1 | 1 | 0 | 2 | 0 | 1 | 0 | 1 | 0 | 1 | 1 | 0 | 0 | 1 | 1 | 0 | 0 | 1 | 0 | 1 |
| 5 | Debregeasia_orientalis_De15 | 1 | 1 | 0 | 2 | 0 | 1 | 0 | 1 | 0 | 1 | 1 | 0 | 0 | 1 | 1 | 0 | 0 | 1 | 0 | 1 |
| 6 | Debregeasia_orientalis_De13 | 1 | 1 | 0 | 2 | 0 | 1 | 0 | 1 | 0 | 1 | 1 | 0 | 0 | 1 | 1 | 0 | 0 | 1 | 0 | 1 |
| 7 | Debregeasia_squamata_De5 | 1 | 1 | 0 | 2 | 0 | 1 | 0 | 1 | 0 | 1 | 1 | 0 | 0 | 1 | 1 | 0 | 0 | 1 | 0 | 1 |
| 8 | Debregeasia_longifolia_De10 | 1 | 1 | 0 | 2 | 0 | 1 | 0 | 1 | 0 | 1 | 1 | 0 | 6 | 1 | 1 | 0 | 0 | 1 | 0 | 1 |
| 9 | Debregeasia_longifolia_De9 | 1 | 1 | 0 | 2 | 0 | 1 | 0 | 1 | 0 | 1 | 1 | 0 | 6 | 1 | 1 | 0 | 0 | 1 | 0 | 1 |
| 10 | Debregeasia_elliptica_De19 | 1 | 1 | 0 | 2 | 0 | 1 | 0 | 1 | 0 | 1 | 1 | 0 | 0 | 1 | 1 | 0 | 0 | 1 | 0 | 1 |
| 11 | Debregeasia_elliptica_De7 | 1 | 1 | 0 | 2 | 0 | 1 | 0 | 1 | 0 | 1 | 1 | 0 | 0 | 1 | 1 | 0 | 0 | 1 | 0 | 1 |
| 12 | Boehmeria_glomerulifera_B5 | 1 | 1 | 0 | 1 | 0 | 1 | 0 | 0 | 1 | 1 | 1 | 0 | 0 | 1 | 1 | 0 | 0 | 1 | 0 | 1 |
| 13 | Boehmeria_macrophylla_var_macrophylla_B28 | 0 | 1 | 0 | 1 | 1 | 1 | 0 | 0 | 1 | 1 | 1 | 0 | 0 | 1 | 1 | 0 | 0 | 1 | 0 | 1 |
| 14 | Boehmeria_macrophylla_var_macrophylla_B24 | 0 | 1 | 0 | 1 | 1 | 1 | 0 | 0 | 1 | 1 | 1 | 0 | 0 | 1 | 1 | 0 | 0 | 1 | 0 | 1 |
| 15 | Boehmeria_sp_B46 | 0 | 1 | 0 | 1 | 1 | 1 | 0 | 0 | 1 | 1 | 1 | 0 | 0 | 1 | 1 | 0 | 0 | 1 | 0 | 1 |
| 16 | Boehmeria_spicata_B9 | 0 | 1 | 0 | 1 | 1 | 1 | 0 | 0 | 1 | 1 | 1 | 0 | 0 | 1 | 1 | 0 | 0 | 1 | 0 | 1 |
| 17 | Boehmeria_japonica_B47 | 0 | 1 | 0 | 1 | 1 | 1 | 0 | 0 | 1 | 1 | 1 | 0 | 0 | 1 | 1 | 0 | 0 | 1 | 0 | 1 |
| 18 | Boehmeria_tricuspis_B39 | 0 | 1 | 0 | 1 | 1 | 1 | 0 | 0 | 1 | 1 | 1 | 0 | 0 | 1 | 1 | 0 | 0 | 1 | 0 | 1 |
| 19 | Boehmeria_longispica_B20 | 0 | 1 | 0 | 1 | 1 | 1 | 0 | 0 | 1 | 1 | 1 | 0 | 0 | 1 | 1 | 0 | 0 | 1 | 0 | 1 |
| 20 | Boehmeria_densiflora_B52 | 1 | 1 | 0 | 1 | 1 | 1 | 0 | 0 | 1 | 1 | 1 | 0 | 0 | 1 | 1 | 0 | 0 | 1 | 0 | 1 |
| 21 | Boehmeria_densiflora_B53 | 1 | 1 | 0 | 1 | 1 | 1 | 0 | 0 | 1 | 1 | 1 | 0 | 0 | 1 | 1 | 0 | 0 | 1 | 0 | 1 |
| 22 | Boehmeria_clidemioides_var_clidemioides_B2 | 0 | 1 | 0 | 1 | 1 | 1 | 0 | 0 | 1 | 1 | 1 | 0 | 0 | 1 | 1 | 0 | 0 | 1 | 0 | 1 |
| 23 | Boehmeria_macrophylla_var_rotundifolia_B21 | 0 | 1 | 0 | 1 | 1 | 1 | 0 | 0 | 1 | 1 | 1 | 0 | 0 | 1 | 1 | 0 | 0 | 1 | 0 | 1 |
| 24 | Boehmeria_clidemioides_var_clidemioides_B15 | 0 | 1 | 0 | 1 | 1 | 1 | 0 | 0 | 1 | 1 | 1 | 0 | 0 | 1 | 1 | 0 | 0 | 1 | 0 | 1 |
| 25 | Boehmeria_zollingeriana_var_blinii_B1 | 0 | 1 | 0 | 1 | 1 | 1 | 0 | 0 | 1 | 1 | 1 | 0 | 0 | 1 | 1 | 0 | 0 | 1 | 0 | 1 |
| 26 | Boehmeria_penduliflora_B33 | 0 | 1 | 0 | 1 | 0 | 1 | 0 | 0 | 1 | 1 | 1 | 0 | 0 | 1 | 1 | 0 | 0 | 1 | 0 | 1 |
| 27 | Boehmeria_umbrosa_B40 | 0 | 1 | 0 | 1 | 1 | 1 | 0 | 0 | 1 | 1 | 1 | 0 | 0 | 1 | 1 | 0 | 0 | 1 | 0 | 1 |
| 28 | Boehmeria_umbrosa_B12 | 0 | 1 | 0 | 1 | 1 | 1 | 0 | 0 | 1 | 1 | 1 | 0 | 0 | 1 | 1 | 0 | 0 | 1 | 0 | 1 |
| 29 | Boehmeria_clidemioides_var_diffusa_B16 | 0 | 1 | 0 | 1 | 0 | 1 | 0 | 0 | 1 | 1 | 1 | 0 | 0 | 1 | 1 | 0 | 0 | 1 | 0 | 1 |
| 30 | Boehmeria_macrophylla_var_scabrella_B26 | 0 | 1 | 0 | 1 | 1 | 1 | 0 | 0 | 1 | 1 | 1 | 0 | 0 | 1 | 1 | 0 | 0 | 1 | 0 | 1 |
| 31 | Chamabainia_cuspidata_C1 | 0 | 1 | 0 | 0 | 1 | 1 | 2 | 0 | 1 | 1 | 1 | 0 | 5 | 1 | 1 | 0 | 0 | 1 | 0 | 1 |
| 32 | Chamabainia_cuspidata_C2 | 0 | 1 | 0 | 0 | 1 | 1 | 2 | 0 | 1 | 1 | 1 | 0 | 5 | 1 | 1 | 0 | 0 | 1 | 0 | 1 |
| 33 | Neraudia_melastomifolia_Ne1 | 1 | 1 | 0 | 1 | 0 | 1 | 2 | 1 | 0 | 1 | 1 | 0 | 5 | 1 | 1 | 0 | 0 | 1 | 0 | 1 |
| 34 | Neraudia_kauaiensis_Ne2 | 1 | 1 | 0 | 1 | 0 | 1 | 2 | 1 | 0 | 1 | 1 | 0 | 5 | 1 | 1 | 0 | 0 | 1 | 0 | 1 |
| 35 | Pouzolzia_argenteonitida_Po5 | 1 | 1 | 0 | 1 | 0 | 1 | 2 | 0 | 1 | 1 | 1 | 0 | 5 | 1 | 1 | 0 | 0 | 1 | 0 | 1 |
| 36 | Meniscogyne_sp_154A | ? | ? | ? | ? | ? | ? | ? | ? | ? | ? | ? | ? | ? | ? | ? | ? | ? | ? | ? | ? |
| 37 | Boehmeria_rugulosa_B45 | 1 | 1 | 0 | 1 | 0 | 1 | 0 | 0 | 1 | 1 | 1 | 0 | 0 | 1 | 1 | 0 | 0 | 1 | 0 | 1 |
| 38 | Pouzolzia_sanguinea_var_sanguinea_Po6 | 1 | 1 | 0 | 1 | 0 | 1 | 2 | 0 | 1 | 1 | 1 | 0 | 5 | 1 | 1 | 0 | 0 | 1 | 0 | 1 |
| 39 | Pouzolzia_sanguinea_var_elegans_Po2 | 1 | 1 | 0 | 1 | 0 | 1 | 2 | 0 | 1 | 1 | 1 | 0 | 5 | 1 | 1 | 0 | 0 | 1 | 0 | 1 |
| 40 | Nothocnide_mollissima_23585 | 1 | 1 | 0 | 1 | 0 | 1 | 0 | 1 | 0 | 1 | 1 | 0 | 0 | 1 | 1 | 0 | 0 | 1 | 0 | 1 |
| 41 | Pipturus_kauaiensis_Pip5 | 1 | 1 | 0 | 1 | 0 | 1 | 0 | 1 | 0 | 1 | 1 | 0 | 5 | 1 | 1 | 0 | 0 | 1 | 0 | 1 |
| 42 | Pipturus_ruber_Pip6 | 1 | 1 | 0 | 1 | 0 | 1 | 0 | 1 | 0 | 1 | 1 | 0 | 5 | 1 | 1 | 0 | 0 | 1 | 0 | 1 |
| 43 | Pipturus_arborescens_Pip7 | 1 | 1 | 0 | 1 | 0 | 1 | 0 | 1 | 0 | 1 | 1 | 0 | 5 | 1 | 1 | 0 | 0 | 1 | 0 | 1 |
| 44 | Pipturus_arborescens_Pip1 | 1 | 1 | 0 | 1 | 0 | 1 | 0 | 1 | 0 | 1 | 1 | 0 | 5 | 1 | 1 | 0 | 0 | 1 | 0 | 1 |
| 45 | Gonostegia_hirta_Go3 | 0 | 1 | 0 | 1 | 1 | 1 | 1 | 1 | 0 | 1 | 1 | 0 | 5 | 1 | 1 | 0 | 0 | 1 | 0 | 1 |
| 46 | Gonostegia_pavifolia_Go1 | 0 | 1 | 0 | 1 | 1 | 1 | 1 | 1 | 0 | 1 | 1 | 0 | 5 | 1 | 1 | 0 | 0 | 1 | 0 | 1 |
| 46 | Gonostegia_pavifolia_Go4 | 0 | 1 | 0 | 1 | 1 | 1 | 1 | 1 | 0 | 1 | 1 | 0 | 5 | 1 | 1 | 0 | 0 | 1 | 0 | 1 |
| 47 | Pouzolzia_zeylanica_var_zeylanica_Po7 | 0 | 1 | 0 | 1 | 1 | 1 | 2 | 0 | 1 | 1 | 1 | 0 | 5 | 1 | 1 | 0 | 0 | 1 | 0 | 1 |
| 48 | Pouzolzia_sp_Po9 | 0 | 1 | 0 | 1 | 1 | 1 | 2 | 0 | 1 | 1 | 1 | 0 | 5 | 1 | 1 | 0 | 0 | 1 | 0 | 1 |
| 50 | Pouzolzia_zeylanica_var_zeylanica_Po4 | 0 | 1 | 0 | 1 | 1 | 1 | 2 | 0 | 1 | 1 | 1 | 0 | 5 | 1 | 1 | 0 | 0 | 1 | 0 | 1 |
| 51 | Pouzolzia_guineensis_282A | 0 | 1 | 0 | 1 | 0 | 1 | 2 | 0 | 1 | 1 | 1 | 0 | 5 | 1 | 1 | 0 | 0 | 1 | 0 | 1 |
| 52 | Pouzolzia_mixta_288A | 1 | 1 | 0 | 1 | 0 | 1 | 2 | 0 | 1 | 1 | 1 | 0 | 5 | 1 | 1 | 0 | 0 | 1 | 0 | 1 |
| 53 | Hemistylus_macrostachya_23597 | 1 | 1 | 0 | 1 | 0 | 1 | 1 | 0 | 1 | 1 | 1 | 0 | 5 | 1 | 1 | 0 | 0 | 1 | 0 | 1 |
| 54 | Rousselia_humulis_23596 | 0 | 1 | 0 | 1 | 0 | 1 | 0 | 0 | 1 | 1 | 1 | 0 | 5 | 1 | 1 | 0 | 0 | 1 | 0 | 1 |
| 55 | Neodistemon_indicum_279A | 0 | 1 | 0 | 1 | 0 | 1 | 2 | 0 | 1 | 1 | 1 | 0 | 5 | 1 | 1 | 0 | 0 | 1 | 0 | 1 |
| 56 | Oreocnide_frutescens_subsp_frutescens_O8 | 1 | 1 | 0 | 5 | 0 | 1 | 0 | 0 | 1 | 1 | 1 | 0 | 4 | 1 | 1 | 0 | 0 | 1 | 0 | 1 |
| 57 | Oreocnide_frutescens_subsp_frutescens_O2 | 1 | 1 | 0 | 5 | 0 | 1 | 0 | 0 | 1 | 1 | 1 | 0 | 4 | 1 | 1 | 0 | 0 | 1 | 0 | 1 |
| 58 | Oreocnide_frutescens_subsp_occidentalis_O12 | 1 | 1 | 0 | 5 | 0 | 1 | 0 | 0 | 1 | 1 | 1 | 0 | 4 | 1 | 1 | 0 | 0 | 1 | 0 | 1 |
| 59 | Soleirolia_soleirolii_312A | 0 | 1 | 0 | 0 | 0 | 0 | - | - | - | 1 | 1 | 1 | 5 | 1 | 1 | 0 | 0 | 1 | 0 | 1 |
| 60 | Gesnouinia_arborea_177A | 1 | 1 | 0 | 1 | 0 | 0 | - | - | - | 1 | 1 | 0 | 5 | 1 | 1 | 0 | 0 | 1 | 0 | 1 |
| 61 | Parietaria_judaica_11077 | 0 | 1 | 0 | 0 | 0 | 0 | - | - | - | 1 | 1 | 0 | 5 | 1 | 1 | 0 | 0 | 1 | 0 | 1 |
| 62 | Parietaria_micrantha_Pa1 | 0 | 1 | 0 | 0 | 0 | 0 | - | - | - | 1 | 1 | 0 | 5 | 1 | 1 | 0 | 0 | 1 | 0 | 1 |
| 63 | Forsskaolea_angustifolia_16132 | 0 | 1 | 0 | 1 | 0 | 1 | 1 | 0 | 1 | 0 | - | 0 | 0 | 1 | 1 | 0 | 1 | 1 | 0 | 0 |
| 64 | Forsskaolea_angustifolia_6515 | 0 | 1 | 0 | 1 | 0 | 1 | 1 | 0 | 1 | 0 | - | 0 | 0 | 1 | 1 | 0 | 1 | 1 | 0 | 0 |
| 65 | Droguetia_ambigua_28892 | 0 | 1 | 0 | 1 | 1 | 1 | 0 | 0 | 1 | 1 | 1 | 0 | 0 | 1 | 1 | 0 | 1 | 1 | 0 | 1 |
| 66 | Droguetia_iner_subsp_urticoides_Dr1 | 0 | 1 | 0 | 1 | 1 | 1 | 0 | 0 | 1 | 1 | 1 | 0 | 0 | 1 | 1 | 0 | 1 | 1 | 0 | 1 |
| 67 | Droguetia_iner_subsp_urticoides_Dr4 | 0 | 1 | 0 | 1 | 1 | 1 | 0 | 0 | 1 | 1 | 1 | 0 | 0 | 1 | 1 | 0 | 1 | 1 | 0 | 1 |
| 68 | Australina_flaccida_23601 | 0 | 1 | 0 | 1 | 1 | 1 | 0 | 0 | 1 | 1 | 1 | 0 | 3 | 1 | 1 | 0 | 1 | 1 | 0 | 1 |
| 69 | Didymodoxa_caffra_23599 | 0 | 1 | 0 | 1 | 0 | 1 | 0 | 0 | 1 | 1 | 1 | 0 | 5 | 1 | 1 | 0 | 1 | 1 | 0 | 1 |
| 70 | Phenax_mexicanus_378A | 1 | 1 | 0 | 1 | 0 | 1 | 0 | 0 | 1 | 0 | - | 0 | 3 | 1 | 1 | 0 | 0 | 1 | 0 | 1 |
| 71 | Coussapoa_parvifolia_386A | 1 | 0 | - | 0 | 0 | 1 | 0 | 1 | 0 | 1 | 1 | 0 | 4 | 1 | 0 | - | 0 | 1 | 1 | 1 |
| 72 | Myrianthus_preussii_23604 | 1 | 0 | - | 4 | 0 | 1 | 0 | 1 | 0 | 1 | 1 | 0 | ? | 1 | 1 | 3 | 0 | 1 | 1 | 1 |
| 73 | Cecropia_ficifolia_23606 | 1 | 0 | - | 5 | 0 | 1 | 0 | 1 | 0 | 1 | 1 | 0 | 3 | 1 | 1 | 3 | 0 | 1 | 1 | 1 |
| 74 | Cecropia_obtusifolia_162A | 1 | 0 | - | 5 | 0 | 1 | 0 | 1 | 0 | 1 | 1 | 0 | 0 | 1 | 1 | 3 | 0 | 1 | 1 | 1 |
| 75 | Leucosyke_quadrinervia_Leu3 | 1 | 0 | - | 0 | 0 | 1 | 0 | 1 | 0 | 1 | 1 | 1 | 3 | 1 | 1 | 3 | 0 | 1 | 0 | 1 |
| 76 | Leucosyke_quadrinervia_Leu4 | 1 | 0 | - | 0 | 0 | 1 | 0 | 1 | 0 | 1 | 1 | 1 | 3 | 1 | 1 | 3 | 0 | 1 | 0 | 1 |
| 77 | Maoutia_setosa_M2 | 1 | 0 | - | 0 | 0 | 1 | 0 | 1 | 0 | 1 | 1 | 0 | 3 | 1 | 1 | 0 | 0 | 1 | 0 | 1 |
| 78 | Fatoua_villosa_F2 | 0 | 0 | - | 1 | 0 | 1 | 3 | 0 | 1 | 1 | 0 | 1 | 6 | 1 | 1 | 0 | 0 | 1 | 0 | 1 |
| 79 | Fatoua_villosa_F1 | 0 | 0 | - | 1 | 0 | 1 | 3 | 0 | 1 | 1 | 0 | 1 | 6 | 1 | 1 | 0 | 0 | 1 | 0 | 1 |
| 80 | Humulus_scandense_H1 | 0 | 0 | - | 1 | 1 | 1 | 3 | 0 | 1 | 1 | 1 | 0 | 0 | 1 | 1 | 3 | 0 | 1 | 1 | 1 |
| 81 | Celtis_kunmingensis_Ulm2 | 1 | 0 | - | 1 | 0 | 1 | ? | ? | ? | 1 | ? | ? | ? | 1 | 1 | 0 | 0 | 1 | 1 | 1 |
| 82 | Pellionia_tsoongii_Pe5 | 0 | 1 | 1 | 0 | 0 | 1 | 1 | 0 | 1 | 1 | 0 | 0 | 3 | 1 | 1 | 1 | 0 | 1 | 0 | 1 |
| 83 | Pellionia_macrophylla_Pe1 | 0 | 1 | 1 | 0 | 0 | 1 | 0 | 0 | 1 | 1 | 0 | 0 | 2 | 1 | 0 | - | 0 | 1 | 0 | 1 |
| 84 | Pellionia_radicans_Pe3 | 0 | 1 | 1 | 0 | 0 | 1 | 3 | 0 | 1 | 1 | 0 | 0 | 3 | 1 | 1 | 1 | 0 | 1 | 0 | 1 |
| 85 | Pellionia_paucidentata_var_paucidentata_Pe2 | 0 | 1 | 1 | 0 | 0 | 1 | 3 | 0 | 1 | 1 | 0 | 0 | 3 | 1 | 1 | 1 | 0 | 1 | 0 | 1 |
| 86 | Elatostema_parvum_var_parvum_E7 | 0 | 1 | 1 | 0 | 0 | 1 | 3 | 0 | 1 | 1 | 0 | 0 | 1 | 1 | 1 | 1 | 0 | 1 | 0 | 1 |
| 87 | Elatostema_densistriolatum_E9 | 0 | 1 | 1 | 0 | 0 | 1 | 5 | 0 | 1 | 1 | 0 | 0 | 2 | 1 | 1 | 0 | 0 | 1 | 0 | 1 |
| 88 | Elatostema_petelotii_E8 | 0 | 1 | 1 | 0 | 0 | 1 | 3 | 0 | 1 | 1 | 0 | 0 | 0 | 1 | 1 | 0 | 0 | 1 | 0 | 1 |
| 89 | Elatostema_cuspidatum_var_cuspidatum_E4 | 0 | 1 | 1 | 0 | 0 | 1 | 3 | 0 | 1 | 1 | 0 | 0 | 1 | 1 | 1 | 1 | 0 | 1 | 0 | 1 |
| 90 | Elatostema_crytandrifolium_var_crytandrifolium_E3 | 0 | 1 | 1 | 0 | 0 | 1 | 2 | 0 | 1 | 1 | 0 | 0 | 1 | 1 | 1 | 1 | 0 | 1 | 0 | 1 |
| 91 | Elatostema_tenuicaudatum_var_tenuicaudatum_E12 | 0 | 1 | 1 | 0 | 0 | 1 | 3 | 0 | 1 | 1 | 0 | 0 | 1 | 1 | 1 | 2 | 0 | 1 | 0 | 1 |
| 92 | Elatostema_longibracteatum_E6 | 0 | 1 | 1 | 0 | 0 | 1 | 3 | 0 | 1 | 1 | 0 | 0 | 1 | 1 | 1 | 1 | 0 | 1 | 0 | 1 |
| 93 | Elatostema_stewardii_E10 | 0 | 1 | 1 | 0 | 0 | 1 | 2 | 0 | 1 | 1 | 0 | 0 | 1 | 1 | 0 | - | 0 | 1 | 0 | 1 |
| 94 | Elatostema_atropurpureum_E2 | 0 | 1 | 1 | 0 | 0 | 1 | 2 | 0 | 1 | 1 | 0 | 0 | 1 | 1 | 0 | - | 0 | 1 | 0 | 1 |
| 95 | Elatostema_albopilosum_E1 | 0 | 1 | 1 | 0 | 0 | 1 | 3 | 0 | 1 | 1 | 0 | 0 | 1 | 1 | 0 | - | 0 | 1 | 0 | 1 |
| 96 | Elatostema_subtrichotomum_var_subtrichotomum_E11 | 0 | 1 | 1 | 0 | 0 | 1 | 3 | 0 | 1 | 1 | 0 | 0 | 3 | 1 | ? | ? | 0 | 1 | 0 | 1 |
| 97 | Elatostema_sp_E13 | 0 | 1 | 1 | 0 | 0 | 1 | 3 | 0 | 1 | 1 | 0 | 0 | 1 | 1 | 1 | 1 | 0 | 1 | 0 | 1 |
| 98 | Pellionia_repens_Pe4 | 0 | 1 | 1 | 0 | 0 | 1 | 1 | 0 | 1 | 1 | 0 | 0 | 3 | 1 | 1 | 1 | 0 | 1 | 0 | 1 |
| 99 | Procris_wightiana_Pr2 | 0 | 1 | 1 | 0 | 0 | 1 | 5 | 1 | 0 | 1 | 0 | 0 | 2 | 1 | 0 | - | 0 | 1 | 0 | 1 |
| 100 | Procris_wightiana_Pr1 | 0 | 1 | 1 | 0 | 0 | 1 | 5 | 1 | 0 | 1 | 0 | 0 | 2 | 1 | 0 | - | 0 | 1 | 0 | 1 |
| 101 | Gyrotaenia_crassifolia_475A | 1 | 1 | 1 | 2 | 0 | 1 | 0 | 0 | 1 | 1 | 0 | 0 | 4 | 1 | 1 | 0 | 0 | 1 | 0 | 1 |
| 102 | Gyrotaenia_microcarpa_473A | 1 | 1 | 1 | 2 | 0 | 1 | 0 | 0 | 1 | 1 | 0 | 0 | 4 | 1 | 1 | 0 | 0 | 1 | 0 | 1 |
| 103 | Myriocarpa_obovata_370A | 1 | 1 | 2 | 8 | 0 | 1 | 0 | 1 | 0 | 1 | 1 | 0 | 0 | 1 | 1 | 0 | 0 | 1 | 0 | 1 |
| 104 | Gyrotaenia_spicata_23567 | 1 | 1 | 1 | 2 | 0 | 1 | 0 | 0 | 1 | 1 | 0 | 0 | 4 | 1 | 1 | 0 | 0 | 1 | 0 | 1 |
| 105 | Myriocarpa_cordata_C2A | 1 | 1 | 2 | 8 | 0 | 1 | 0 | 1 | 0 | 1 | 1 | 0 | 0 | 1 | 0 | - | 0 | 1 | 0 | 1 |
| 106 | Lecanthus_petelotii_var_corniculata_Le4 | 0 | 1 | 1 | 0 | 1 | 1 | 4 | 1 | 0 | 1 | 0 | 0 | 3 | 1 | 1 | 0 | 0 | 1 | 0 | 1 |
| 107 | Lecanthus_peduncularis_Le1 | 0 | 1 | 1 | 0 | 1 | 1 | 4 | 1 | 0 | 1 | 0 | 0 | 3 | 1 | 1 | 0 | 0 | 1 | 0 | 1 |
| 108 | Lecanthus_peduncularis_Le3 | 0 | 1 | 1 | 0 | 1 | 1 | 4 | 1 | 0 | 1 | 0 | 0 | 3 | 1 | 1 | 0 | 0 | 1 | 0 | 1 |
| 109 | Lecanthus_petelotii_var_corniculata_Le2 | 0 | 1 | 1 | 0 | 1 | 1 | 4 | 1 | 0 | 1 | 0 | 0 | 3 | 1 | 1 | 0 | 0 | 1 | 0 | 1 |
| 110 | Pilea_cavaleriei_subsp_cavaleriei_P3 | 0 | 1 | 0 | 0 | 1 | 1 | 2 | 1 | 0 | 1 | 0 | 1 | 0 | 1 | 1 | 0 | 0 | 1 | 0 | 1 |
| 111 | Pilea_pumila_var_pumila_P10 | 0 | 1 | 1 | 0 | 1 | 1 | 2 | 1 | 0 | 1 | 0 | 1 | 6 | 1 | 1 | 0 | 0 | 1 | 0 | 1 |
| 112 | Pilea_melastomoides_P20 | 0 | 1 | 1 | 0 | 1 | 1 | 2 | 1 | 0 | 1 | 0 | 0 | 0 | 1 | 1 | 0 | 0 | 1 | 0 | 1 |
| 113 | Pilea_angulata_subsp_petiplaris_P1 | 0 | 1 | 0 | 0 | 1 | 1 | 4 | 1 | 0 | 1 | 0 | 1 | 6 | 1 | 1 | 0 | 0 | 1 | 0 | 1 |
| 114 | Pilea_verrucossa_subsp_verrucossa_P29 | 0 | 1 | 1 | 0 | 1 | 1 | 2 | 1 | 0 | 1 | 0 | 1 | 6 | 1 | 1 | 0 | 0 | 1 | 0 | 1 |
| 115 | Pilea_martinii_P6 | 0 | 1 | 1 | 0 | 1 | 1 | 0 | 1 | 0 | 1 | 0 | 1 | 0 | 1 | 1 | 0 | 0 | 1 | 0 | 1 |
| 116 | Pilea_sp_P12 | 0 | 1 | 1 | 0 | 1 | 1 | 0 | 1 | 0 | 1 | 0 | 1 | 0 | 1 | 1 | 0 | 0 | 1 | 0 | 1 |
| 117 | Pilea_insolens_P11 | 0 | 1 | 2 | 0 | 1 | 1 | 5 | 1 | 0 | 1 | 0 | 1 | 0 | 1 | 1 | 0 | 0 | 1 | 0 | 1 |
| 118 | Pilea_oxyodon_P9 | 0 | 1 | 2 | 0 | 1 | 1 | 5 | 1 | 0 | 1 | 0 | 1 | 0 | 1 | 1 | 0 | 0 | 1 | 0 | 1 |
| 119 | Sarcopilea_domingensis_302A | 0 | 1 | 1 | 0 | 0 | 1 | 1 | 1 | 0 | 1 | 0 | 0 | 0 | 0 | - | - | 0 | 1 | 0 | 1 |
| 120 | Pilea_microphylla_P21 | 0 | 1 | 1 | 0 | 1 | 1 | 2 | 1 | 0 | 1 | 0 | 1 | 0 | 1 | 1 | 0 | 0 | 1 | 0 | 1 |
| 121 | Pilea_microphylla_P22 | 0 | 1 | 1 | 0 | 1 | 1 | 2 | 1 | 0 | 1 | 0 | 1 | 0 | 1 | 1 | 0 | 0 | 1 | 0 | 1 |
| 122 | Pilea_plantaniflora_P24 | 0 | 1 | 1 | 0 | 1 | 1 | 2 | 1 | 0 | 1 | 0 | 1 | 6 | 1 | 1 | 0 | 0 | 1 | 0 | 1 |
| 123 | Pilea_longipedunculata_P5 | 0 | 1 | 2 | 0 | 1 | 1 | 2 | 1 | 0 | 1 | 0 | 1 | 6 | 1 | 1 | 0 | 0 | 1 | 0 | 1 |
| 124 | Pilea_sinofasciata_P26 | 0 | 1 | 1 | 0 | 1 | 1 | 2 | 1 | 0 | 1 | 0 | 1 | 6 | 1 | 1 | 0 | 0 | 1 | 0 | 1 |
| 125 | Girardinia_diversifolia_subsp_triloba_G6 | 0 | 1 | 0 | 3 | 0 | 1 | 4 | 1 | 0 | 1 | 0 | 1 | 0 | 1 | 1 | 0 | 0 | 0 | 0 | 1 |
| 126 | Girardinia_diversifolia_subsp_triloba_G19 | 0 | 1 | 0 | 3 | 0 | 1 | 4 | 1 | 0 | 1 | 0 | 1 | 0 | 1 | 1 | 0 | 0 | 0 | 0 | 1 |
| 127 | Girardinia_diversifolia_subsp_diversifolia_G9 | 0 | 1 | 0 | 3 | 0 | 1 | 4 | 1 | 0 | 1 | 0 | 1 | 6 | 1 | 1 | 0 | 0 | 0 | 0 | 1 |
| 128 | Girardinia_diversifolia_subsp_diversifolia_G31 | 0 | 1 | 0 | 3 | 0 | 1 | 4 | 1 | 0 | 1 | 0 | 1 | 6 | 1 | 1 | 0 | 0 | 0 | 0 | 1 |
| 129 | Girardinia_diversifolia_subsp_suborbiculata_G17 | 0 | 1 | 0 | 3 | 0 | 1 | 4 | 1 | 0 | 1 | 0 | 1 | 0 | 1 | 1 | 0 | 0 | 0 | 0 | 1 |
| 130 | Girardinia_diversifolia_subsp_suborbiculata_G16 | 0 | 1 | 0 | 3 | 0 | 1 | 4 | 1 | 0 | 1 | 0 | 1 | 0 | 1 | 1 | 0 | 0 | 0 | 0 | 1 |
| 131 | Nanocnide_lobata_N6 | 0 | 1 | 2 | 0 | 0 | 1 | 1 | 0 | 1 | 1 | 0 | 0 | 6 | 1 | 1 | 0 | 0 | 0 | 0 | 1 |
| 132 | Nanocnide_lobata_N5 | 0 | 1 | 2 | 0 | 0 | 1 | 1 | 0 | 1 | 1 | 0 | 0 | 6 | 1 | 1 | 0 | 0 | 0 | 0 | 1 |
| 133 | Nanocnide_japonica_N1 | 0 | 1 | 2 | 0 | 0 | 1 | 1 | 0 | 1 | 1 | 0 | 0 | 0 | 1 | 1 | 0 | 0 | 0 | 0 | 1 |
| 134 | Nanocnide_japonica_N4 | 0 | 1 | 2 | 0 | 0 | 1 | 1 | 0 | 1 | 1 | 0 | 0 | 0 | 1 | 1 | 0 | 0 | 0 | 0 | 1 |
| 135 | Urtica_sp_U19 | 0 | 1 | 0 | 0 | 1 | 1 | 3 | 0 | 1 | 1 | 0 | 0 | 6 | 1 | 1 | 0 | 0 | 0 | 0 | 1 |
| 136 | Urtica_triangularis_subsp_pinnatifida_U10 | 0 | 1 | 0 | 0 | 1 | 1 | 3 | 0 | 1 | 1 | 0 | 0 | 6 | 1 | 1 | 0 | 0 | 0 | 0 | 1 |
| 137 | Urtica_hyperborea_U5 | 0 | 1 | 0 | 0 | 1 | 1 | 4 | 0 | 1 | 1 | 0 | 0 | 0 | 1 | 1 | 0 | 0 | 0 | 0 | 1 |
| 138 | Urtica_hyperborea_U14 | 0 | 1 | 0 | 0 | 1 | 1 | 4 | 0 | 1 | 1 | 0 | 0 | 0 | 1 | 1 | 0 | 0 | 0 | 0 | 1 |
| 139 | Urtica_atrichocaulis_U3 | 0 | 1 | 0 | 0 | 1 | 1 | 3 | 0 | 1 | 1 | 0 | 0 | 0 | 1 | 1 | 0 | 0 | 0 | 0 | 1 |
| 140 | Urtica_dioica_U21 | 0 | 1 | 0 | 0 | 1 | 1 | 3 | 0 | 1 | 1 | 0 | 0 | 0 | 1 | 1 | 0 | 0 | 0 | 0 | 1 |
| 141 | Urtica_angustifolia_U1 | 0 | 1 | 0 | 0 | 1 | 1 | 3 | 0 | 1 | 1 | 0 | 0 | 6 | 1 | 1 | 0 | 0 | 0 | 0 | 1 |
| 142 | Urtica_sp_U18 | 0 | 1 | 0 | 0 | 1 | 1 | 3 | 0 | 1 | 1 | 0 | 0 | 0 | 1 | 1 | 0 | 0 | 0 | 0 | 1 |
| 143 | Hesperocnide_tenella_331A | 0 | 1 | 0 | 0 | 1 | 1 | 3 | 0 | 1 | 1 | 1 | 0 | 0 | 1 | 1 | 0 | 0 | 0 | 0 | 1 |
| 144 | Urtica_ardens_U2 | 0 | 1 | 0 | 0 | 1 | 1 | 4 | 1 | 1 | 1 | 0 | 0 | 6 | 1 | 1 | 0 | 0 | 0 | 0 | 1 |
| 145 | Urtica_fissa_U4 | 0 | 1 | 0 | 0 | 1 | 1 | 4 | 1 | 1 | 1 | 0 | 0 | 6 | 1 | 1 | 3 | 0 | 0 | 0 | 1 |
| 146 | Urtica_zayuensis_U17 | 0 | 1 | 0 | 0 | 1 | 1 | 1 | 1 | 1 | 1 | 0 | 0 | 0 | 1 | 1 | 3 | 0 | 0 | 0 | 1 |
| 147 | Urtica_zayuensis_U11 | 0 | 1 | 0 | 0 | 1 | 1 | 1 | 1 | 1 | 1 | 0 | 0 | 0 | 1 | 1 | 3 | 0 | 0 | 0 | 1 |
| 148 | Urtica_mairei_U7 | 0 | 1 | 0 | 0 | 1 | 1 | 4 | 1 | 1 | 1 | 0 | 0 | 6 | 1 | 1 | 3 | 0 | 0 | 0 | 1 |
| 149 | Discocnide_mexicana_167A | 1 | 1 | 0 | 3 | 0 | 1 | 0 | 1 | 0 | 1 | 0 | 1 | 0 | 1 | 1 | 0 | 0 | 0 | 0 | 1 |
| 150 | Dendrocnide_sinuata_D1 | 1 | 1 | 0 | 4 | 0 | 1 | 5 | 1 | 0 | 1 | 0 | 1 | 6 | 1 | 0 | - | 0 | 0 | 0 | 1 |
| 151 | Dendrocnide_meyeniana_D2 | 1 | 1 | 0 | 4 | 0 | 1 | 5 | 1 | 0 | 1 | 0 | 1 | 3 | 1 | 0 | - | 0 | 0 | 0 | 1 |
| 152 | Dendrocnide_urentissima_D5 | 1 | 1 | 0 | 4 | 0 | 1 | 5 | 1 | 0 | 1 | 0 | 1 | 6 | 1 | 0 | - | 0 | 0 | 0 | 1 |
| 153 | Dendrocnide_sp_W1 | 1 | 1 | 0 | 4 | 0 | 1 | 5 | 1 | 0 | 1 | 0 | 1 | 6 | 1 | 0 | - | 0 | 0 | 0 | 1 |
| 154 | Laportea_bulbifera_L5 | 0 | 1 | 0 | 3 | 0 | 1 | 0 | 1 | 0 | 1 | 0 | 1 | 6 | 1 | 1 | 0 | 0 | 0 | 0 | 1 |
| 155 | Laportea_bulbifera_L3 | 0 | 1 | 0 | 3 | 0 | 1 | 0 | 1 | 0 | 1 | 0 | 1 | 6 | 1 | 1 | 0 | 0 | 0 | 0 | 1 |
| 156 | Touchardia_latifolia_T1 | 1 | 1 | 0 | 9 | 0 | 1 | 0 | 1 | 0 | 1 | 0 | 0 | 5 | 1 | 0 | - | 0 | 1 | 0 | 1 |
| 157 | Urera_glabra_Ur1 | 1 | 1 | 0 | 0 | 0 | 1 | 0 | 1 | 0 | 1 | 0 | 1 | 3 | 1 | 0 | - | 0 | 0 | 0 | 1 |
| 158 | Obetia_tenax_28719 | 1 | 1 | 0 | 7 | 0 | 1 | 3 | 0 | 1 | 1 | 0 | 1 | 6 | 1 | 1 | 3 | 0 | 0 | 0 | 1 |
| 159 | Urera_sp_L2 | 1 | 1 | 0 | 4 | 0 | 1 | 0 | 1 | 0 | 1 | 0 | 1 | 6 | 1 | 1 | 0 | 0 | 0 | 0 | 1 |
| 160 | Urera_trinervis_374A | 1 | 1 | 2 | 2 | 0 | 1 | 0 | 1 | 0 | 1 | 1 | 1 | 0 | 1 | 1 | 0 | 0 | 0 | 0 | 1 |
| 161 | Urera_hypselodendron_377A | 1 | 1 | 2 | 2 | 0 | 1 | 0 | 1 | 0 | 1 | 0 | 1 | 3 | 1 | 1 | 0 | 0 | 0 | 0 | 1 |
| 162 | Poikilospermum_suaveolens_Pi2 | 1 | 1 | 0 | 4 | 0 | 1 | 0 | 1 | 0 | 1 | 1 | 0 | 6 | 1 | 0 | - | 0 | 1 | 1 | 1 |
| 163 | Poikilospermum_suaveolens_Pi3 | 1 | 1 | 0 | 4 | 0 | 1 | 0 | 1 | 0 | 1 | 1 | 0 | 6 | 1 | 0 | - | 0 | 1 | 1 | 1 |
| 164 | Poikilospermum_lanceolatum_Pi1 | 1 | 1 | 0 | 4 | 0 | 1 | 0 | 1 | 0 | 1 | 1 | 0 | 6 | 1 | 0 | - | 0 | 1 | 1 | 1 |
| 165 | Urera_baccifera_C4A | 1 | 1 | 0 | 0 | 0 | 1 | 0 | 1 | 0 | 1 | 0 | 1 | 3 | 1 | 0 | - | 0 | 0 | 0 | 1 |
| 166 | Urera_alceifolia_C11A | 1 | 1 | 1 | 2 | 0 | 1 | 0 | 1 | 0 | 1 | 1 | 1 | 0 | 1 | 1 | 0 | 0 | 0 | 0 | 1 |
| 167 | Urera_lianoides_313A | 1 | 1 | 0 | 4 | 0 | 1 | 0 | 1 | 0 | 1 | 0 | 1 | 6 | 1 | 1 | 0 | 0 | 0 | 0 | 1 |
| 168 | Urera_caracasana_23561 | 1 | 1 | 0 | 2 | 0 | 1 | 0 | 1 | 0 | 1 | 0 | 0 | 3 | 1 | 0 | - | 0 | 0 | 0 | 1 |
| 169 | Boehmeria_nivea_var_nipononivea_B32 | 1 | 1 | 0 | 1 | 0 | 1 | 0 | 0 | 1 | 1 | 1 | 0 | 0 | 1 | 1 | 0 | 0 | 1 | 0 | 1 |
| 170 | Boehmeria_nivea_var_nivea_B6 | 1 | 1 | 0 | 1 | 0 | 1 | 0 | 0 | 1 | 1 | 1 | 0 | 0 | 1 | 1 | 0 | 0 | 1 | 0 | 1 |
| 171 | Boehmeria_tomentosa_B38 | 0 | 1 | 0 | 1 | 1 | 1 | 0 | 0 | 1 | 1 | 1 | 0 | 0 | 1 | 1 | 0 | 0 | 1 | 0 | 1 |
| 172 | Sarcochlamys_pulcherrima_S1 | 1 | 1 | 0 | 6 | 0 | 1 | 0 | 0 | 1 | 1 | 0 | 1 | 0 | 1 | 1 | 0 | 0 | 1 | 0 | 1 |
| 173 | Archiboehmeria_atrata_A2 | 0 | 1 | 0 | 4 | 0 | 1 | 0 | 1 | 0 | 1 | 1 | 0 | 6 | 1 | 1 | 0 | 0 | 1 | 0 | 1 |
